# Supplementary material for: The phase behavior of skin-barrier lipids: A combined approach of experiments and simulations
Source: Biophys J. 2024 Jul 18;123(18):3188–204. doi: 10.1016/j.bpj.2024.07.018 (PMC11447553; doi:10.1016/j.bpj.2024.07.018)
Supplement: Document S1. Figures S1–S12 and Tables S1–S7 [file mmc1.pdf]

Biophysical Journal, Volume *123*

Supplemental information

The phase behavior of skin-barrier lipids: A combined approach of experiments and simulations

Parashara Shamaprasad, Andreea Nădăban, Christopher R. Iacovella, Gerrit S. Gooris, Annette L. Bunge, Joke A. Bouwstra, and Clare M^c^Cabe

Supplemental Material

The phase behavior of skin-barrier lipids: a combined approach of experiments and simulations

Parashara Shamaprasad[a], Andreea Nădăban[b], Christopher R. Iacovella[a], Gerrit S. Gooris[b], Annette L. Bunge[c], Joke A. Bouwstra[b], Clare McCabe[a,d].

[a] Department of Chemical and Biomolecular Engineering, Vanderbilt University, Nashville, TN 37235-1604, United States of America
[b] Division of BioTherapeutics, Leiden Academic Centre for Drug Research, Leiden University, 2333 CC Leiden, the Netherlands
[c] Department of Chemical and Biological Engineering, Colorado School of Mines, Golden, CO 80401, United States of America
[d] School of Engineering and Physical Science, Heriot-Watt University, Edinburgh, United Kingdom

Corresponding Author: Clare McCabe
Corresponding Author Email: c.mccabe@hw.ac.uk

Contents:

Description of coarse-grained simulation methodology

Figure S1. Atomic schematics (carbons in green, oxygens in red, nitrogen in blue, and hydrogens in gray) of A) FFA C24 and B) CER NS C24 molecules showing the 12-carbon section in each chain (circled) that were used in the S2 calculations (Figures 8A and S5).

Figure S2. Snapshots from CG simulations of the 3 bilayer (6 leaflet) membranes of CER NS C24:CHOL:FFA C24 with molar ratios of (A) 1:0:1, (B) 1:0.2:1, (C) 1:0.5:1, (D) and 1:1:1.

Figure S3. Scatter plots of the local bilayer thickness versus local composition of each lipid chain in the central bilayer of 3-bilayer membranes containing a 1:0.2:1 molar ratio mixture of CER NS C24:CHOL:FFA C24.

Figure S4. The δCH_2 vibration at 10°C (A), and the thermotropic behavior (B) of the CER NS:CHOL: FFA24 (1:0.5:1) protiated model.

Figure S5. The density of the 3-bilayer membrane and the nematic order parameter (S2) for FFA C24, and the sphingosine and acyl chains of CER NS C24 in the central bilayer of a 3-bilayer membrane plotted as a function of temperature to show the hexagonal-to-fluid phase transition between 65°C and 80°C and the fluid-to-isotropic phase transition between 95°C and 105°C.

Figure S6. Average change in the S_{CH} order parameters for all carbons on the FFA C24 chain, and the sphingosine and acyl chains of CER NS C24 in the four inner leaflets of the 3-bilayer membrane at the specified temperature (T) relative to the lowest temperature simulated (285 K, 11.85ºC).

Figure S7. S_{CH} order parameter calculated for the FFA molecules in the four inner leaflets of the 3-bilayer membrane (i.e., all leaflets that are not in direct contact with bulk water).

Figure S8. S_{CH} order parameter calculated for the sphingosine chain of CER NS molecules in the four inner leaflets of the 3-bilayer membrane.

Figure S9. S_{CH} order parameter calculated for the acyl chain of CER NS molecules in the four inner leaflets of the 3-bilayer membrane.

Figure S10. Temperature variation of structural properties calculated from atomistic simulations of the reverse-mapped 3-bilayer membrane containing CER NS C24:CHOL:FFA C24 with a 1:0.5:1 molar ratio

Figure S11. Explanation of the FTIR CD_2 scissoring vibrations and a schematic representation of the CERs conformation.

Figure S12. Spearman correlation coefficient between the local fraction of each tail type and local bilayer thickness of the central bilayer in a 3-bilayer system

Table S1. Systems studied by composition and method used.

Table S2. Comparison of the molar ratios for each lipid tail type (normalized by the average number of CER NS sphingosine tails) and the total number of lipid tails in the central bilayer and two outer bilayers of a 3-bilayer membrane containing a 1:0.5:1 molar ratio mixture of CER NS C24:CHOL:FFA C24.

Table S3. Raw count of neighboring pairs of lipid tails for the central bilayer of a 3-bilayer membrane containing a 1:0.5:1 molar ratio mixture of CER NS C24:CHOL:FFA C24.

Table S4. Number of each lipid tail type in the central bilayer of a 3-bilayer membrane containing a 1:0.5:1 molar ratio mixture of CER NS C24:CHOL:FFA C24.

Table S5. Raw values of the coordination number representing the number of each lipid tail type (listed by column) that neighbor the reference lipid tail type (listed in the first column) in the central bilayer of a 3-bilayer membrane containing a 1:0.5:1 molar ratio mixture of CER NS C24:CHOL:FFA C24.

Table S6. Normalized coordination numbers representing the number of each of the six lipid tail types (listed by column) that neighbor the reference lipid tail type (listed in the first column) in the central bilayer of a 3-bilayer membrane containing a 1:0.5:1 molar ratio mixture of CER NS C24:CHOL:FFA C24.

Table S7. Normalized lipid tail pair count ratio from the nearest neighbor analysis (column 3), listed in descending order, compared with the fraction of each lipid tail type that neighbors the reference lipid tail calculated from the normalized coordination numbers.

Description of the coarse-grained simulation methodology

The coarse-grained simulation trajectories of the 3-bilayer simulations of CER NS C24:CHOL:FFA C24 in 1:0:1, 1:0.2:1, 1:0.5:1, and 1:1:1 molar ratios analyzed in this work were generated as described in Shamaprasad et al. (1). Simulations were initialized with a box of 2000 lipids with a density of 0.8 g/mL surrounded in the positive and negative z-dimensions by boxes of 10000 water beads with a density of 1.0 g/mL (20000 water beads in total). This is equivalent to 40 water molecules per lipid since the CG model maps 4 water molecules to each water bead.

All coarse-grained simulations used a 10 fs timestep and were run using the HOOMD-Blue 2.9.7 simulation engine (2). The coarse-grained force field was optimized using the multistate iterative Boltzmann inversion (MS IBI) method (3), in which both bulk fluid and lamellar states were used as targets for optimization (1, 4–6). The systems were first relaxed with a brief 20 ps simulation using the microcanonical (NVE) ensemble with a 0.1 Å limit on the displacement of each bead at every timestep to remove high energy overlaps. Next, a 10 ns simulation is performed in the isothermal-isobaric (NPT) ensemble at 305 K and 1 atm with an isotropic barostat to equilibrate the system density while keeping the aspect ratio of the box constant. The systems then undergo the shape annealing procedure at 305 K in the canonical (NVT) ensemble, in which the lateral area is expanded to double the expected final lateral area over 200 ns, compressed to the expected final lateral area over another 200 ns, and held constant for an additional 200 ns. The expected final lateral area for each system is set based on the expected average area per lipid, which is a function of the lipid composition. The simulations are then equilibrated using the NPT ensemble for 300 ns. To eliminate any defects in the structures, an additional temperature annealing step in the NVT ensemble is conducted, where the box dimensions are held constant and the temperature is increased from 305 K to 375 K over 50 ns, held at 375 K for 100 ns, and cooled to 305 K over 50 ns. The system is then equilibrated in the NPT ensemble at 305 K and 1 atm for 500 ns. In the last 200 ns of this final simulation, the trajectory is recorded every 0.1 ns to produce 2000 frames that were used in the data analyses of this paper. All simulations in the NVT ensemble use the Nosé-Hoover thermostat (7, 8) with a temperature coupling constant of 1 ps, and simulations in the NPT ensemble use the Martyna-Tobias-Klein barostat-thermostat (9–12) with temperature and pressure coupling constants of 1 ps and 10 ps, respectively.

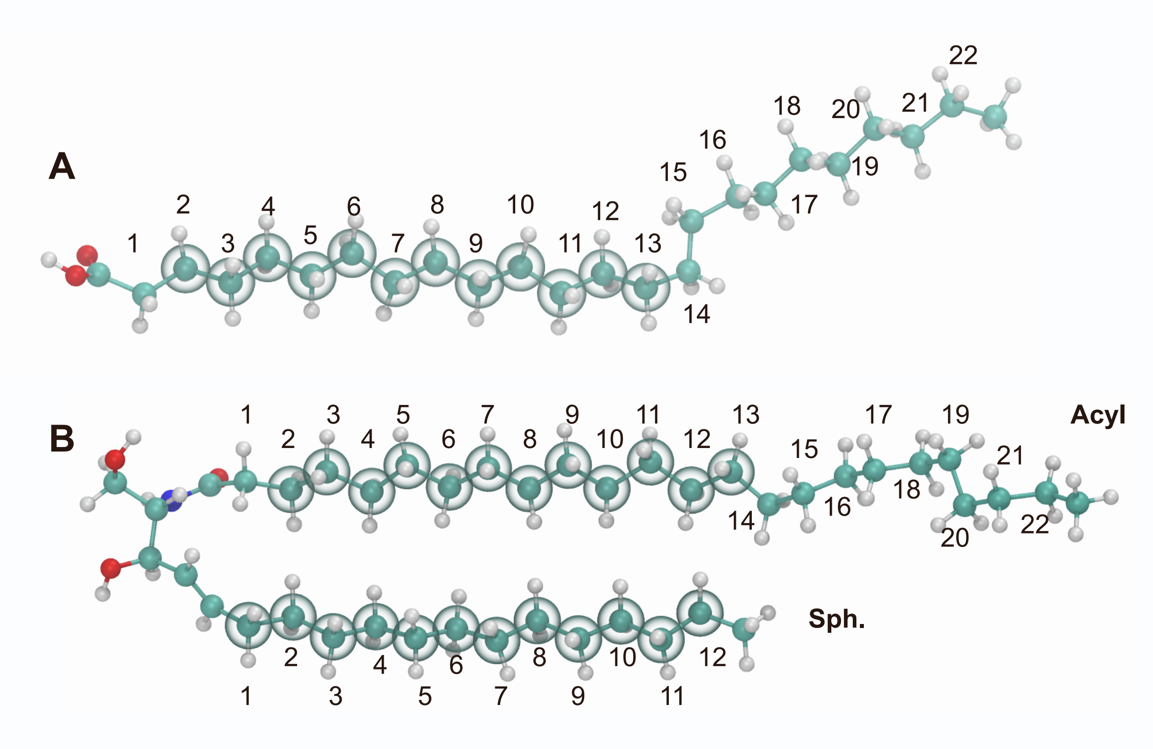

Figure S1. Atomic schematics (carbons in green, oxygens in red, nitrogen in blue, and hydrogens in gray) of A) FFA C24 and B) CER NS C24 molecules showing the 12-carbon section in each chain (circled) that were used in the S2 calculations (Figures 8A and S5) so that the director is calculated within regions that are well-ordered at low temperature (i.e., the ends of the chains, which tend to be disordered in the interdigitated regime, even at low temperature, are excluded). Numbers listed on each molecule correspond to the carbon numbers in plots of the S_{CH} shown in Figures S7 – S9.

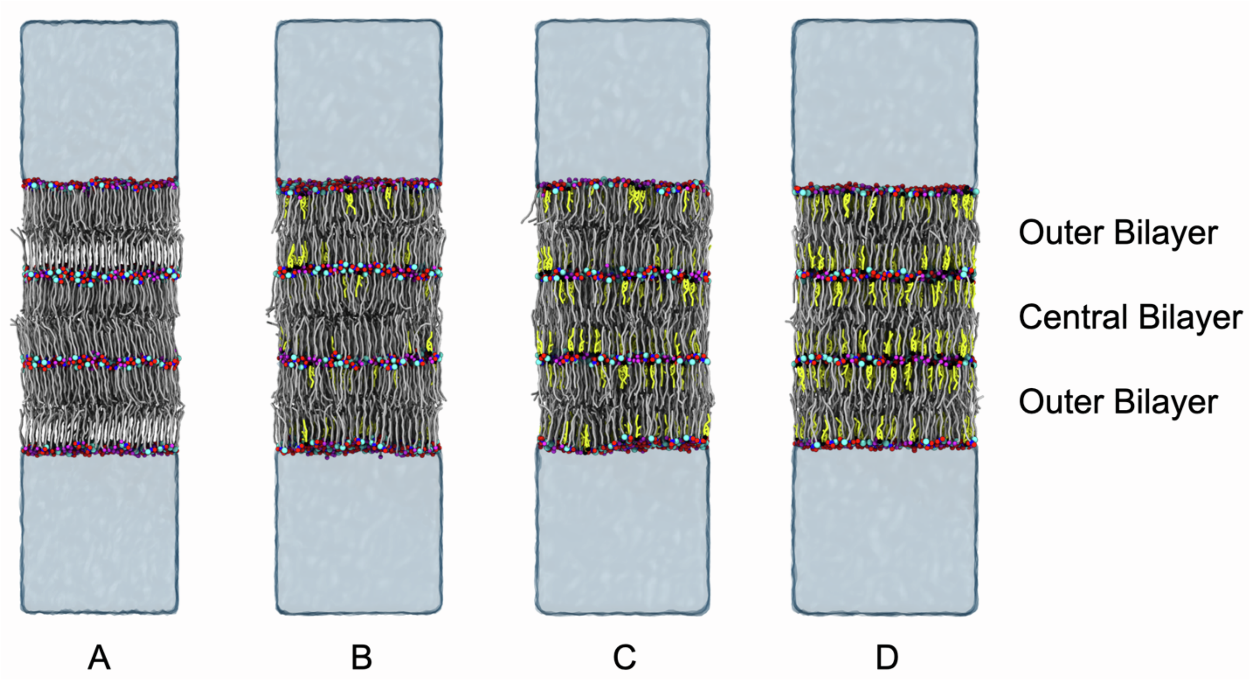

Figure S2. Snapshots from CG simulations of the 3 bilayer (6 leaflet) membranes of CER NS C24:CHOL:FFA C24 with molar ratios of (A) 1:0:1, (B) 1:0.2:1, (C) 1:0.5:1, (D) and 1:1:1. The central bilayer (consisting of the two innermost leaflets) is sandwiched between the two outer bilayers. Lipid backbones are represented as a stick model (CER and FFA tails in gray, and CHOL in yellow), with headgroup CG beads (as depicted in Figure 1 of the paper) rendered as spheres (MHEAD2, cyan; AMIDE, blue; OH1 and OH2, red; FHEAD, purple; and CHEAD, black). Reprinted with permission from Shamaprasad, et al. (1).

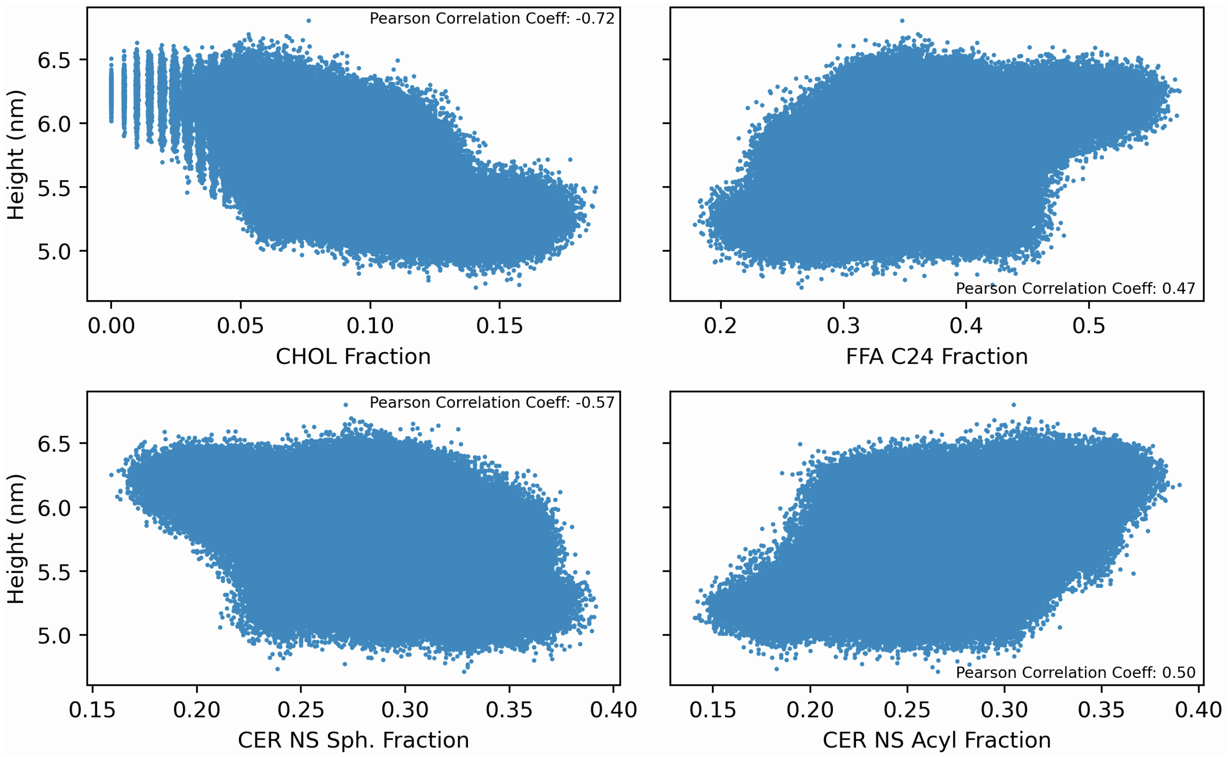

Figure S3. Scatter plots of the local bilayer thickness versus local composition of each lipid tail in the central bilayer of 3-bilayer membranes containing a 1:0.2:1 molar ratio mixture of CER NS C24:CHOL:FFA C24.

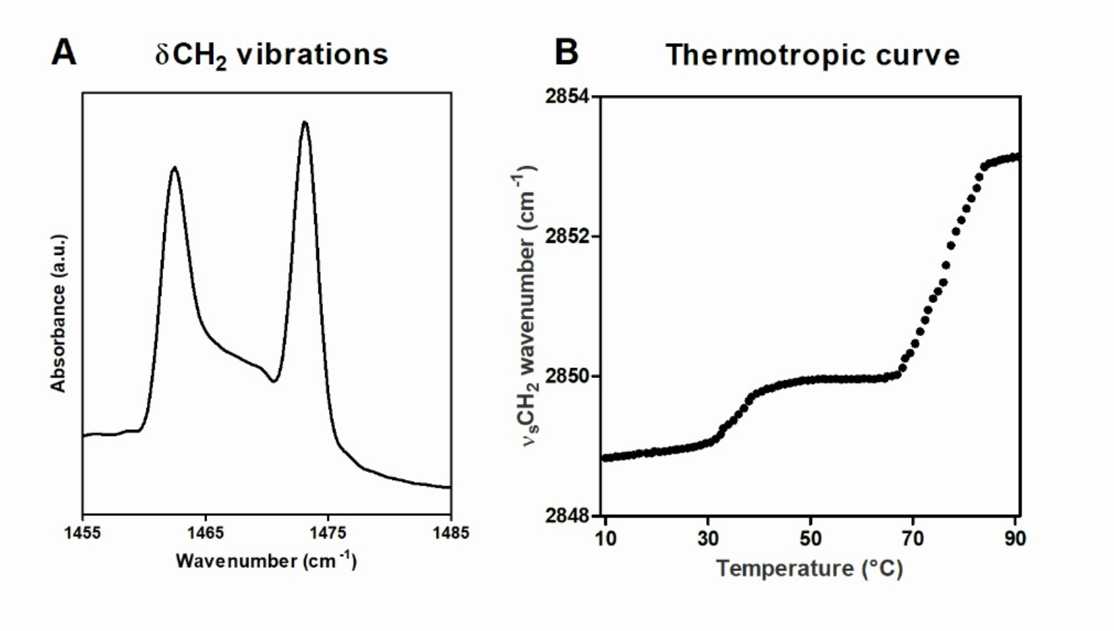

Figure S4. The δCH_2 vibration at 10°C (A), and the thermotropic behavior (B) of the CER NS:CHOL: FFA24 (1:0.5:1) protiated model. The thermotropic curve shows the $\nu_s CH_2$ wavenumbers as a function of temperature in the range 10-90°C.

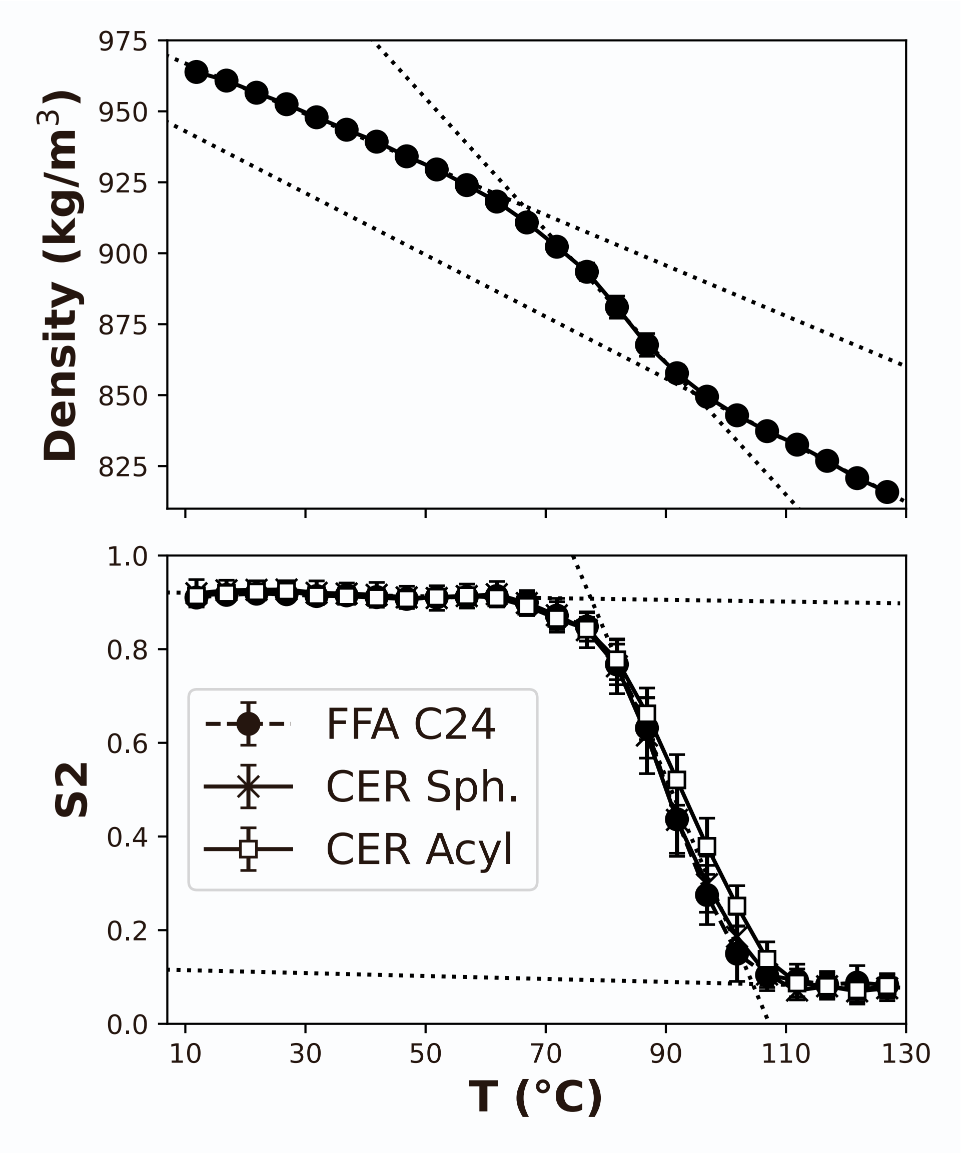

Figure S5. The density of the 3-bilayer membrane and the nematic order parameter (S2) for FFA C24, and the sphingosine and acyl chains of CER NS C24 in the central bilayer of a 3-bilayer membrane plotted as a function of temperature to show the hexagonal-to-fluid phase transition between 65°C and 80°C and the fluid-to-isotropic phase transition between 95°C and 105°C. Results presented here were calculated as described for those presented in Figure 8. Best-fit lines are included for the three regimes representing the hexagonal, fluid and isotropic phases. S2 values were calculated for the 12 carbon atoms designated in Figure S1 and reported as the mean and standard deviation of the average over the final 200 ns (2000 frames) of one simulation.

Descriptions of Figures S6 – S9

Figure S6 shows the average change in the S_{CH} order parameters (calculated as described in the Methods section) for all carbons on the FFA C24 chain and for sphingosine and acyl chains of CER NS C24 in the four central leaflets of the 3-bilayer membrane (i.e., all leaflets that are not in direct contact with bulk water) at the specified temperature relative to the lowest temperature simulated (285 K, 11.85ºC). Plots of the S_{CH} order parameters for each carbon in the FFA chain and in the sphingosine and acyl chains of the four central leaflets are presented in Figures S7, S8 and S9, respectively. Carbon numbers in Figures S7-S9 correspond to those shown in Figure S1. Values of S_{CH} range from –0.5 representing a lipid chain with the C-H bonds oriented perpendicular to the bilayer normal and a value of S_{CH} = 1 indicating C-H bonds are parallel to the bilayer normal (13).

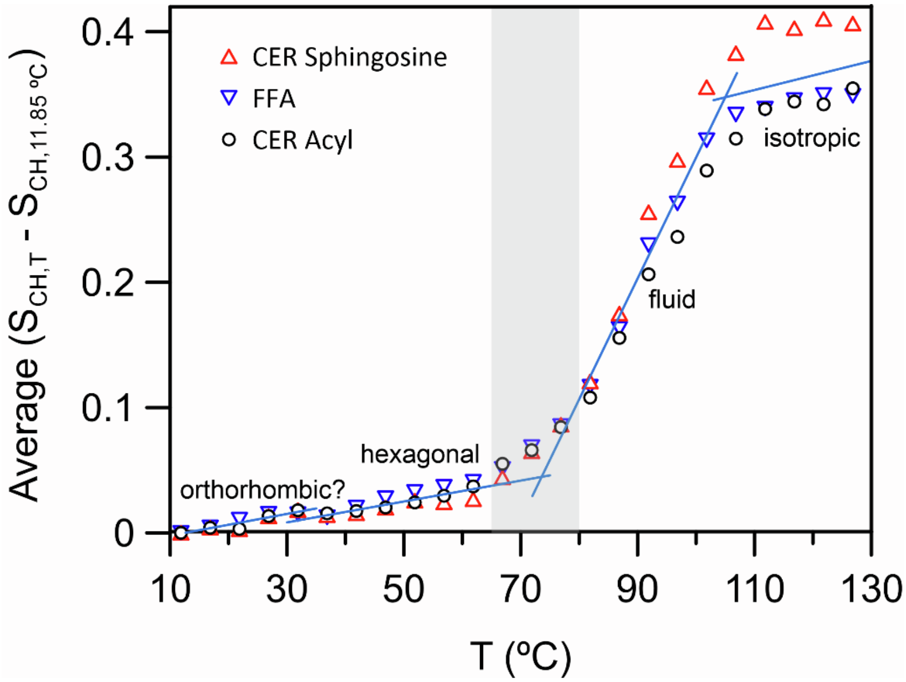

Figure S6. Average change in the S_{CH} order parameters for all carbons on the FFA C24 chain, and the sphingosine and acyl chains of CER NS C24 in the four inner leaflets of the 3-bilayer membrane at the specified temperature (T) relative to the lowest temperature simulated (285 K, 11.85ºC). The gray shading designates the estimated 65°C to 80°C range of the hexagonal-fluid phase transition.

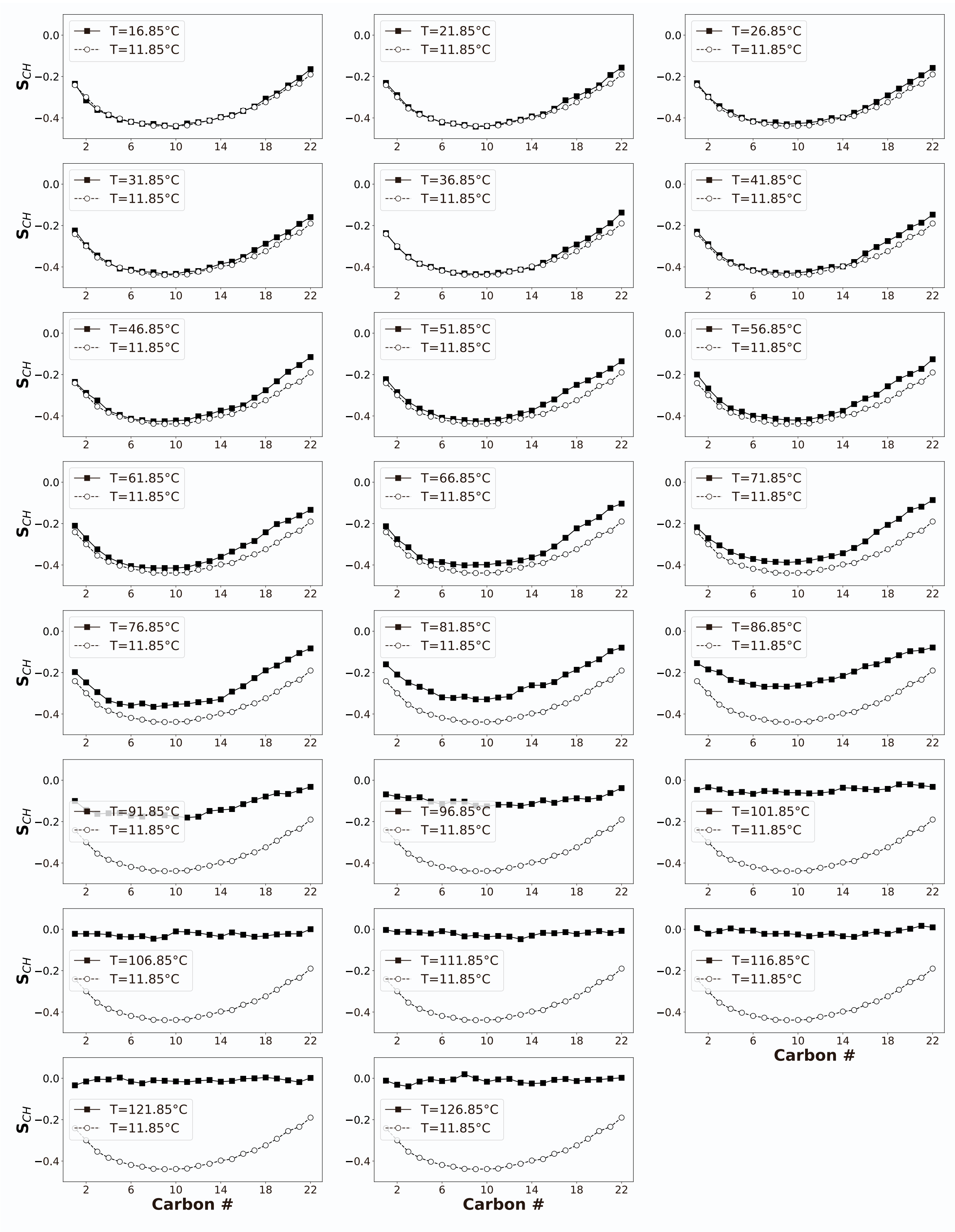

Figure S7. S_{CH} order parameter calculated for the FFA molecules in the four inner leaflets of the 3-bilayer membrane (i.e., all leaflets that are not in direct contact with bulk water) calculated at the designated temperatures as described in the Methods section. Carbon numbers correspond to those shown in Figure S1.

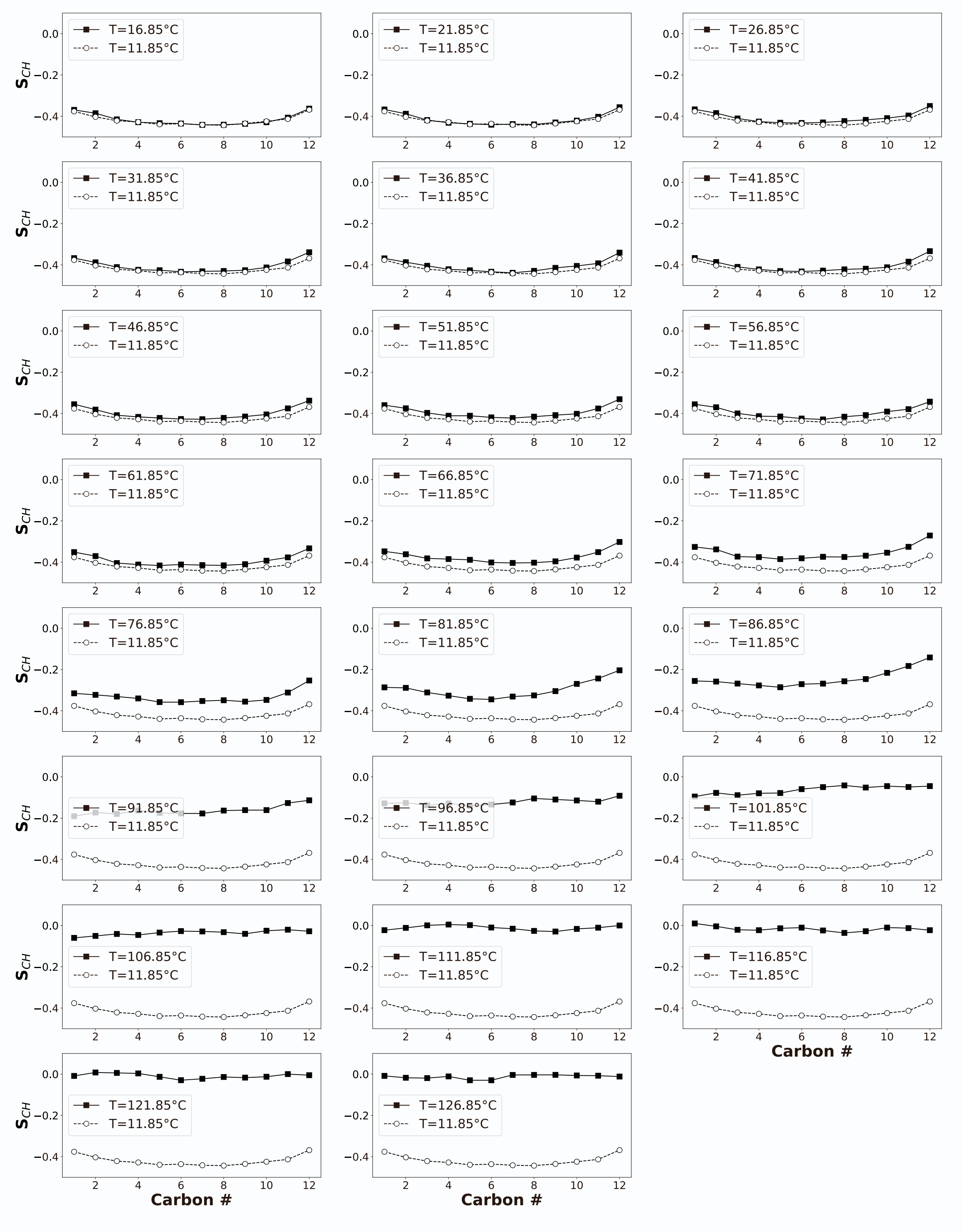

Figure S8. S_{CH} order parameter calculated for the sphingosine chain of CER NS molecules in the four inner leaflets of the 3-bilayer membrane (i.e., all leaflets that are not in direct contact with bulk water) calculated at the designated temperatures as described in the Methods section. Carbon numbers correspond to those shown in Figure S1.

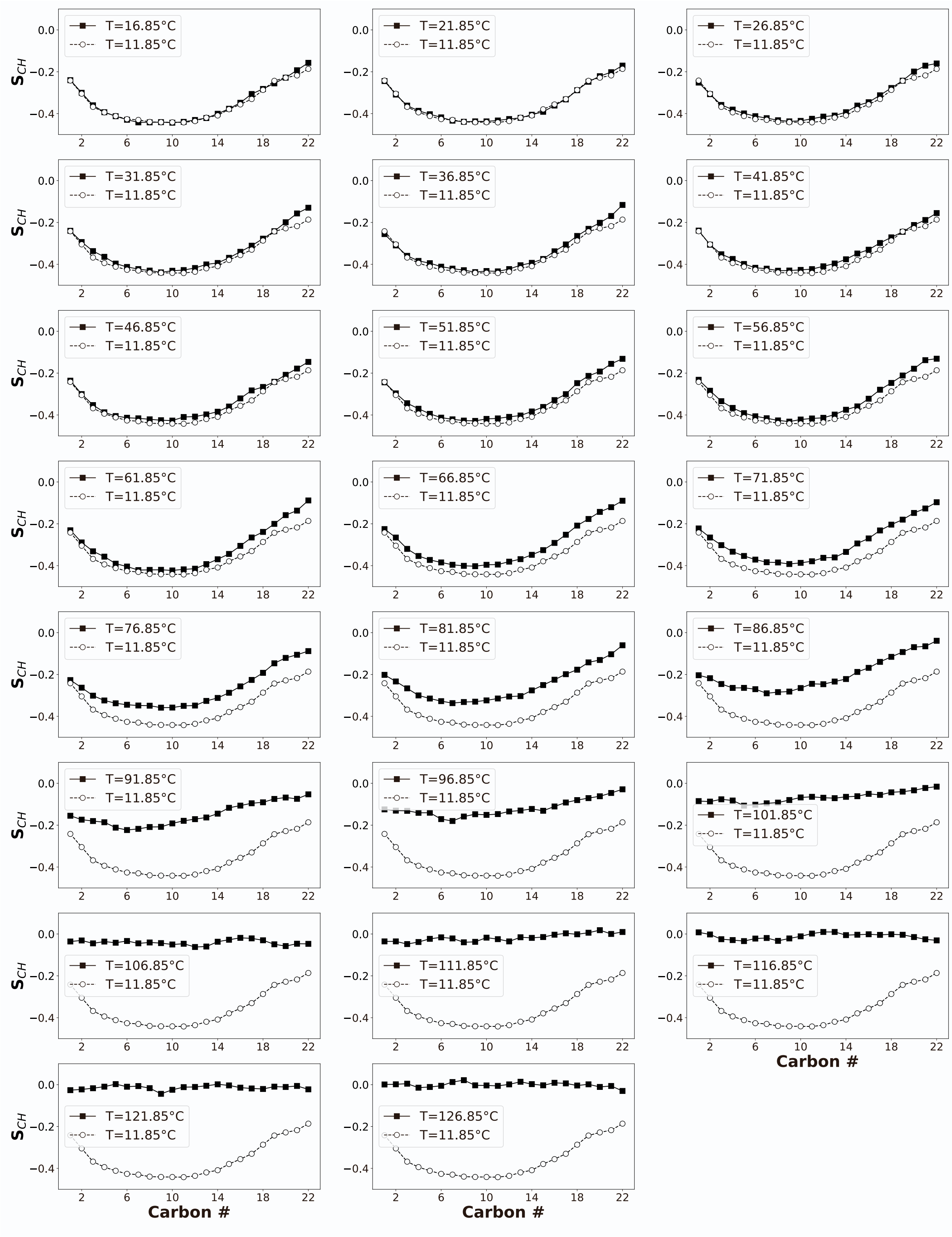

Figure S9. S_{CH} order parameter calculated for the acyl chain of CER NS molecules in the four inner leaflets of the 3-bilayer membrane (i.e., all leaflets that are not in direct contact with bulk water) calculated at the designated temperatures as described in the Methods section. Carbon numbers correspond to those shown in Figure S1.

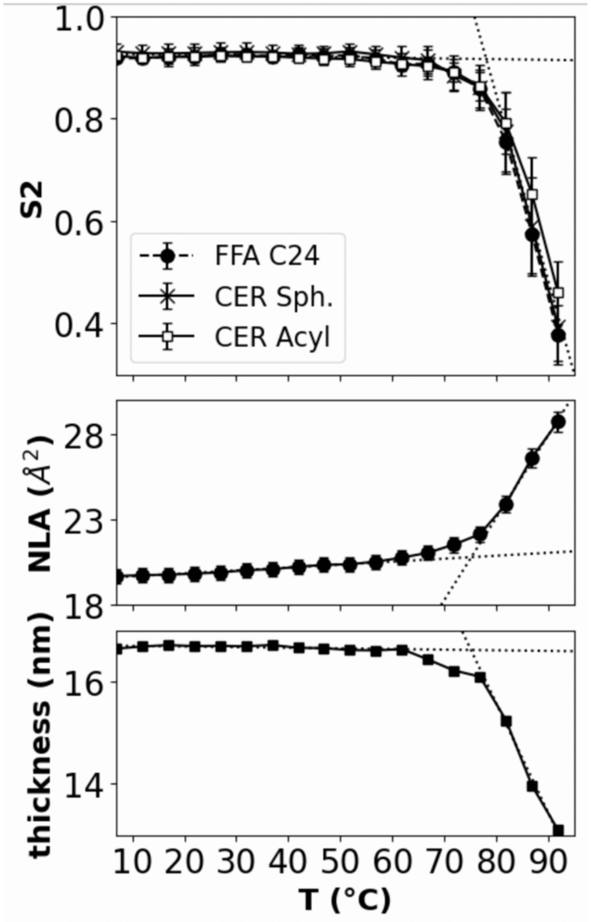

Figure S10. Temperature variation of structural properties calculated from atomistic simulations of the reverse-mapped 3-bilayer membrane containing CER NS C24:CHOL:FFA C24 with a 1:0.5:1 molar ratio heated at a rate of 2.5 °C per ns from 280K. Structural properties obtained using this procedure have very similar transition temperatures and overall behavior as is observed in Figure 8 in the main text, which combines independent heating (32-127ºC) and cooling (32-7ºC) simulations.

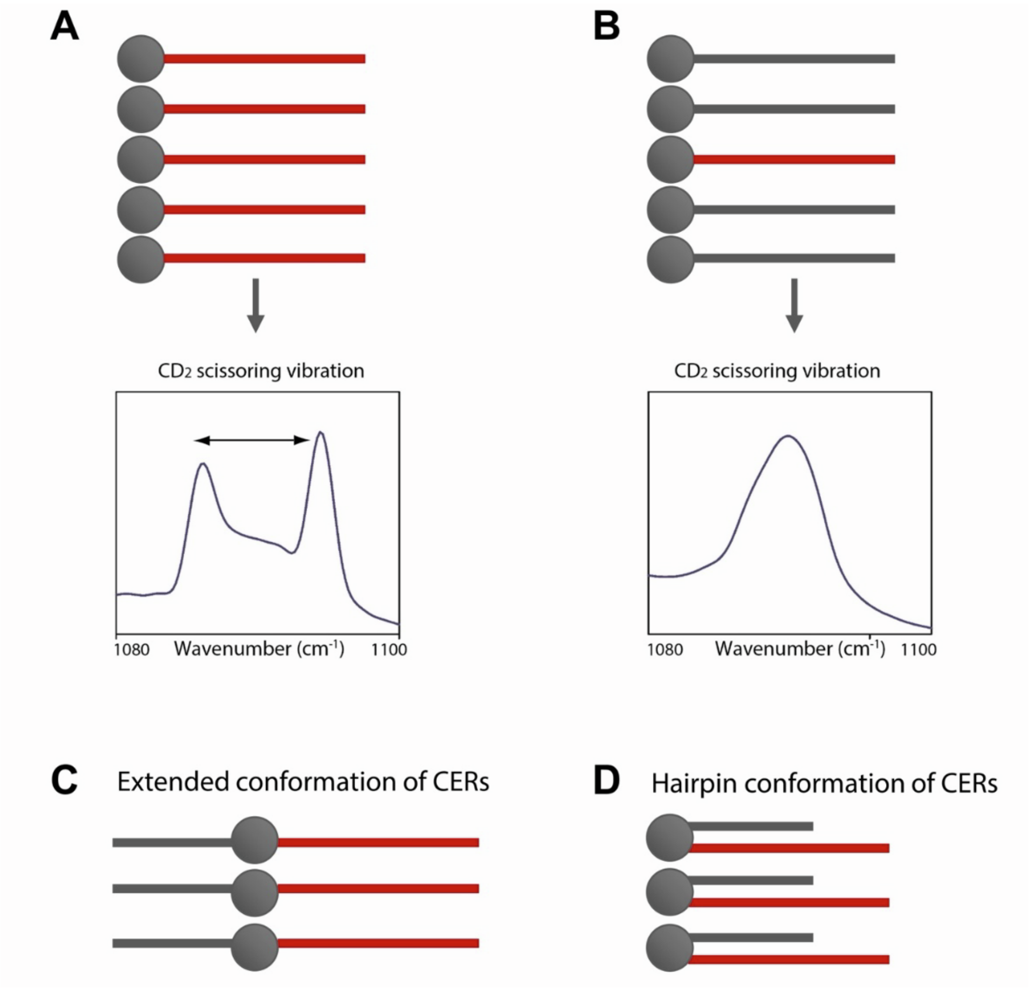

Figure S11. (A) Schematic drawing of the deuterated lipid chains (red) neighboring each other forming large deuterated domains; the large number of CD_2-CD_2 interactions causes two peaks in the FTIR spectrum for the δCD_2 vibrations. (B) When an isolated deuterated lipid chain (red) is present in a protiated lipid chain environment (grey), a single peak occurs in the FTIR spectrum for the δCD_2 vibrations, as there are no CD_2-CD_2 chain interactions. (C) A schematic representation of the extended conformation of CERs, with the acyl chain deuterated (red) and the sphingoid base protiated (grey) located on either side of the CER headgroup. (D) A schematic representation of the CER hairpin conformation, with the two chains on the same side of the headgroup.

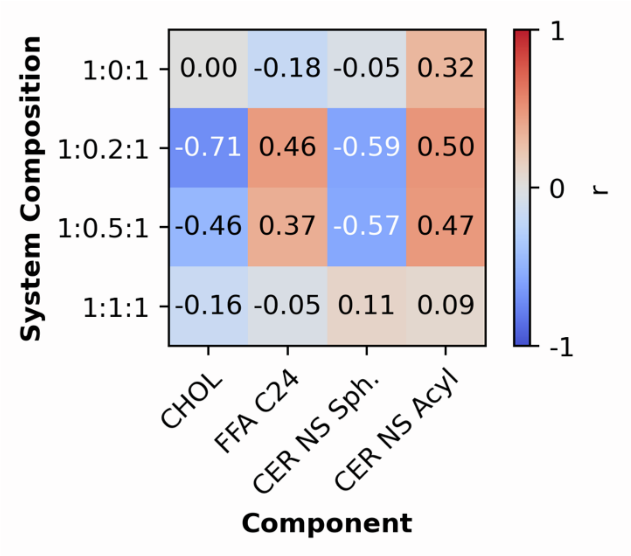

Figure S12. Spearman correlation coefficient between the local fraction of each tail type and local bilayer thickness of the central bilayer in a 3-bilayer system tabulated separately for CHOL, FFA C24, and the sphingosine and acyl tails of CER NS (x axis) for four mixtures at the indicated CER NS:CHOL:FFA molar ratios (y axis). Results are aggregated over four independent simulations at each composition. White text designates |r| > 0.5.

Table S1. Systems studied by composition and method used.

System Composition (CER NS C24: CHOL: FFA C24)	Experiment		Simulation			
	SAXD	FTIR	CG			Reverse-mapped all atom
	Repeat distances and presence of lamellar phases	Phase transition temperatures and tail interactions	Local bilayer thickness and composition	Domain composition using Gaussian mixture clustering	Nearest neighbor analysis	Phase transition temperature
1:0:1	X		X			
1:0.05:1	X					
1:0.1:1	X					
1:0.2:1	X		X	X		
1:0.5:1	X	X	X		X	X
1:1:1	X		X			

Table S2. Comparison of the molar ratios for each lipid tail type (normalized by the average number of CER NS sphingosine tails) and the total number of lipid tails in the central bilayer and two outer bilayers of a 3-bilayer membrane containing a 1:0.5:1 molar ratio mixture of CER NS C24:CHOL:FFA C24. Acyl and sphingosine chains of CER NS are treated separately. Values listed are the mean and standard deviation of the average over the final 200 ns (2000 frames) of 4 replicate simulations.

CER:CHOL:FFA molar ratio	Bilayer(s)	CER NS Acyl	CER NS Sphingosine	CHOL	FFA C24	Total Number of Lipid Tails
1:0:1	Central	0.96 ± 0.07	1.00 ± 0.04		0.85 ± 0.11	942 ± 23
	Outer	1.02 ± 0.07	1.00 ± 0.08		1.05 ± 0.16	2037 ± 26
1:0.2:1	Central	0.99 ± 0.09	1.00 ± 0.10	0.28 ± 0.05	1.23 ± 0.15	941 ± 10
	Outer	1.00 ± 0.18	1.00 ± 0.19	0.20 ± 0.05	0.89 ± 0.23	1954 ± 9
1:0.5:1	Central	0.96 ± 0.04	1.00 ± 0.07	0.49 ± 0.05	0.91 ± 0.05	923 ± 4
	Outer	1.02 ± 0.04	1.00 ± 0.04	0.50 ± 0.03	1.05 ± 0.07	1874 ± 6
1:1:1	Central	1.05 ± 0.06	1.00 ± 0.05	1.14 ± 0.03	1.11 ± 0.05	878 ± 10
	Outer	0.98 ± 0.10	1.00 ± 0.10	0.94 ± 0.05	0.95 ± 0.13	1789 ± 10

Table S3. Raw count of neighboring pairs of lipid tails for the central bilayer of a 3-bilayer membrane containing a 1:0.5:1 molar ratio mixture of CER NS C24:CHOL:FFA C24. Acyl and sphingosine chains of CER NS in the hairpin and extended conformations are treated separately. Values listed are the mean and standard deviation of the average over the final 200 ns (2000 frames) of 4 replicate simulations.

	CER NS Acyl Hairpin	CER NS Acyl Extended	CER NS Acyl All	CER NS Sph Hairpin	CER NS Sph Extended	CER NS Sph All	CHOL	FFA C24
**CER NS Acyl Hairpin**	77.8 ± 4.8	104.3 ± 7.4		328.6 ± 8.1	118.3 ± 6.9		159.8 ± 10.3	223.2 ± 24.7
**CER NS Acyl Extended**	104.3 ± 7.4	35.6 ± 8.4		89.5 ± 8.9	60.7 ± 5.7		85.5 ± 12.8	144.5 ± 16.7
**CER NS Acyl All**			217.7 ± 18.4			597.0 ± 10.7	245.3 ± 13.8	367.7 ± 19.4
**CER NS Sph Hairpin**	328.6 ± 8.1	89.5 ± 8.9		81.8 ± 4.7	109.2 ± 10.3		176.4 ± 10.4	222.4 ± 14.8
**CER NS Sph Extended**	118.3 ± 6.9	60.7 ± 5.7		109.2 ± 10.3	38.0 ± 5.2		107.5 ± 18.4	152.2 ± 17.0
**CER NS Sph All**			597.0 ± 10.7			229.0 ± 14.2	283.9 ± 19.2	374.7 ± 27.3
**CHOL**	159.8 ± 10.3	85.5 ± 12.8	245.3 ± 13.8	176.4 ± 10.4	107.5 ± 18.4	283.9 ± 19.2	66.8 ± 4.0	299.4 ± 8.5
**FFA C24**	223.2 ± 24.7	144.5 ± 16.7	367.7 ± 19.4	222.4 ± 14.8	152.2 ± 17.0	374.7 ± 27.3	299.4 ± 8.5	259.4 ± 21.0

Table S4. Number of each lipid tail type in the central bilayer of a 3-bilayer membrane containing a 1:0.5:1 molar ratio mixture of CER NS C24:CHOL:FFA C24. Acyl and sphingosine tails of CER NS in the hairpin and extended conformations are treated separately. Values listed are the mean and standard deviation of the averages over the final 200 ns (2000 frames) of 4 replicate simulations. These values were used in the calculation of the normalized lipid counts in Table 3 of the paper.

Tail	Count
CER NS Acyl Hairpin	174.50 ± 3.57
CER NS Acyl Extended	89.00 ± 9.51
CER NS Acyl	263.50 ± 7.16
CER NS Sph. Hairpin	174.50 ± 3.57
CER NS Sph. Extended	99.75 ± 6.94
CER NS Sph.	274.25 ± 7.63
CHOL	135.25 ± 5.07
FFA C24	250.00 ± 10.89

Table S5. Raw values of the coordination number representing the number of each lipid tail type (listed by column) that neighbor the reference lipid tail type (listed in the first column) in the central bilayer of a 3-bilayer membrane containing a 1:0.5:1 molar ratio mixture of CER NS C24:CHOL:FFA C24.[a] Acyl and sphingosine chains of CER NS in the hairpin and extended conformations are treated separately. The sum of each row is equal to the total number of neighbors: 7 for CHOL and 6 for all other lipid tails. Values listed are the mean of the average over the final 200 ns (2000 frames) of 4 replicate simulations.

	**Raw Mean Number of Neighbors (Coordination Number)**						
**Reference Lipid Tail**	**CER NS Acyl Extended**	**CER NS Acyl Hairpin**	**CER NS Sph. Extended**	**CER NS Sph. Hairpin**	**CHOL**	**FFA**	**Sum**
**CER NS Acyl Extended**	0.812	1.073	0.679	0.976	0.861	1.600	6
**CER NS Acyl Hairpin**	0.610	0.950	0.635	1.855	0.709	1.241	6
**CER NS sph. Extended**	0.676	1.059	0.888	1.000	0.947	1.429	6
**CER NS sph. Hairpin**	0.553	1.767	0.567	0.817	0.953	1.343	6
**CHOL**	0.671	1.039	0.789	1.355	0.917	2.228	7
**FFA**	0.607	0.814	0.555	0.92	1.059	2.045	6

[a] Coordination number is calculated by averaging the number of each type of neighboring lipid tail for each reference lipid tail, where a neighbor is defined as one of the 6 nearest neighbors (7 for CHOL), by distance, to the reference tail. This analysis was done using MDTraj (14), Freud (15), and the Numpy (16) Python packages.

Table S6. Normalized coordination numbers representing the number of each of the six lipid tail types (listed by column) that neighbor the reference lipid tail type (listed in the first column) in the central bilayer of a 3-bilayer membrane containing a 1:0.5:1 molar ratio mixture of CER NS C24:CHOL:FFA C24. Acyl and sphingosine chains of CER NS in the hairpin and extended conformations are treated separately. The values listed account for differences in the amounts of each lipid tail type. These were calculated by dividing the raw mean coordination numbers listed in Table S5 by the mole fraction of that lipid tail type (listed in the last row of this table), and then normalizing the resulting numbers so that the sum in each row is equal to the total number of neighbors: 7 for CHOL and 6 for all other lipid tails. For a given row, columns with larger normalized values are more preferred as neighbors to the reference lipid tail listed in the first column. Values listed are the mean of the average over the final 200 ns (2000 frames) of 4 replicate simulations.

	**Normalized Mean Number of Neighbors (Coordination Number)**						
**Reference Lipid Tail**	**CER NS Acyl Extended**	**CER NS Acyl Hairpin**	**CER NS Sph. Extended**	**CER NS Sph. Hairpin**	**CHOL**	**FFA**	**Sum**
**CER NS Acyl Extended**	1.297	0.923	1.084	0.839	0.962	0.894	6
**CER NS Acyl Hairpin**	0.993	0.833	1.033	1.626	0.808	0.707	6
**CER NS Sph. Extended**	1.058	0.892	1.389	0.842	1.037	0.782	6
**CER NS Sph. Hairpin**	0.910	1.564	0.932	0.723	1.097	0.773	6
**CHOL**	1.126	0.939	1.325	1.225	1.077	1.309	7
**FFA**	1.025	0.740	0.937	0.837	1.252	1.209	6
**Composition (mole fraction)**	0.599	1.115	0.599	1.115	0.857	1.714	6

Table S7. Normalized lipid tail pair count ratio from the nearest neighbor analysis (column 3), listed in descending order, compared with the fraction of each lipid tail type that neighbors the reference lipid tail calculated from the normalized coordination numbers. [a,b] Results are for the central bilayer of a 3-bilayer membrane containing a 1:0.5:1 molar ratio mixture of CER NS C24:CHOL:FFA C24. Acyl and sphingosine chains of CER NS in the hairpin (HP) and extended (Ext) conformations are treated separately. Values are for the central bilayer in the 3-bilayer stack presented as the mean of the average over the final 200 ns (2000 frames) of 4 replicate simulations. As expected, the normalized pair count ratio from the nearest neighbor analysis provides similar results to the normalized coordination number fraction.

**Lipid Tail 1:Lipid Tail 2 normalized pair count ratio**			**Normalized coordination number fraction of the 6 lipid tail types (listed as either lipid tail 1 or lipid tail 2) that neighbor the reference lipid tail type [b,c]**					
			**Reference lipid tail type**					
**Lipid Tail 1**	**Lipid Tail 2**	**Normalized pair count ratio [a]**	**CER NS Acyl (HP)**	**CER Acyl NS (Ext)**	**CER NS Sph. (HP)**	**CER NS Sph. (Ext)**	**CHOL**	**FFA**
**CER NS Sph. (HP)**	**FFA**	0.738			0.129			0.140
**CER NS Acyl (HP)**	**FFA**	0.740	0.118					0.123
**CER NS Acyl (HP)**	**CER NS Acyl (HP)**	0.744	0.139					
**CER NS Sph. (HP)**	**CER NS Sph. (HP)**	0.782			0.121			
**CER NS Acyl (Ext)**	**CER NS Sph. (HP)**	0.834		0.140	0.152			
**CER NS Sph. (Ext)**	**FFA**	0.883				0.130		0.156
**CER NS Sph. (HP)**	**CER NS Sph. (Ext)**	0.908			0.155	0.140		
**CER NS Acyl (Ext)**	**FFA**	0.940		0.149				***0.171***
**CER NS Acyl (HP)**	**CER NS Acyl (Ext)**	0.972	0.166	0.154				
**CER NS Acyl (HP)**	**CHOL**	0.980	0.135				0.134	
**CER NS Acyl (HP)**	**CER NS Sph. (Ext)**	0.983	***0.172***			0.149		
**CER NS Acyl (Ext)**	**CER NS Sph. (Ext)**	0.989		***0.181***		***0.176***		
**CER NS Acyl (Ext)**	**CHOL**	***1.028***		0.160			0.161	
**CHOL**	**CHOL**	***1.065***					0.154	

Lipid Tail 1:Lipid Tail 2 normalized pair count ratio			Normalized coordination number fraction of the 6 lipid tail types (listed as either lipid tail 1 or lipid tail 2) that neighbor the reference lipid tail type [b,c]					
			Reference lipid tail type					
Lipid Tail 1	Lipid Tail 2	Normalized pair count ratio [a]	CER NS Acyl (HP)	CER Acyl NS (Ext)	CER NS Sph. (HP)	CER NS Sph. (Ext)	CHOL	FFA
CER NS Sph. (HP)	CHOL	***1.081***			***0.183***		***0.175***	
CER NS Sph. (Ext)	CER NS Sph. (Ext)	***1.116***				***0.232***		
CER NS Sph. (Ext)	CHOL	***1.153***				***0.173***	***0.189***	
FFA	FFA	***1.206***						***0.202***
CHOL	FFA	***1.281***					***0.187***	***0.209***
CER NS Acyl (Ext)	CER NS Acyl (Ext)	***1.315***		***0.216***				
CER NS Acyl (HP)	CER NS Sph. (HP)	***1.561***	***0.271***		***0.261***			

[a] The normalized pair count ratio (column 3) indicates the observed occurrence of a lipid tail pair relative to the probability that the lipid tail pair forms based on the numbers of the two lipid tails that form the pair. The numbers listed are the same as those listed in Table 3 of the paper. Values < 1 indicate the lipid chain pair formed less frequently than expected (the lipid tail pair was preferred less); values > 1 (denoted in bold italics) indicate the lipid tail pair formed more frequently than expected (the lipid chain pair was preferred). For example, the most preferred chain pair is the sphingosine and acyl chains of CER NS in the hairpin conformation (normalized pair count ratio = 1.561). The least preferred chain pairs were FFA with either the sphingosine chain or acyl chain of CER NS in the hairpin conformation (normalized pair ratio = 0.738 and 0.740, respectively).

[b] The normalized coordination number fraction of the six lipid tail types that neighbor the reference lipid tail indicates whether a lipid tail type is a neighbor to the reference tail type less often (values < 1/6 = 0.167) or more often (values > 0.167) than would be expected based on the numbers of the lipid tail types. For example, for the acyl chain of CER NS in the hairpin conformation, the sphingosine chain of CER NS in the hairpin conformation is the most preferred neighbor (normalized coordination number fraction = 0.271) and FFA is the least preferred neighbor (normalized coordination number fraction = 0.118). For the FFA chain, CHOL is the most preferred neighbor followed closely by FFA (normalized coordination number fraction = 0.209 and 0.202, respectively), and the acyl chain of CER NS is the least preferred neighbor (normalized coordination number fraction = 0.123)

[c] The normalized mean coordination number fractions of the six lipid tail types that neighbor the reference lipid tail are calculated from the normalized coordination numbers listed in Table S6 divided by the total number of neighbors (7 for CHOL and 6 for all other lipid tails) for (a) lipid tail 2 when tail 1 is the reference lipid tail type (listed under the column for lipid tail 1), and (b) lipid tail 1 when lipid tail 2 is the reference lipid tail type (listed under the column for lipid tail 2). For example, for CHOL as lipid tail 1 and FFA as lipid tail 2, the normalized coordination number fraction listed for CHOL is the normalized coordination number for FFA when CHOL is the reference lipid tail (1.309) divided by 7 (= 0.187); the normalized coordination number listed for FFA is the normalized coordination number for CHOL when FFA is the reference lipid tail (1.252) divided by 6 (= 0.209). When CHOL is both lipid tail 1 and lipid tail 2, then the number listed for CHOL is 1.077 divided 7 (0.154).

References

1. Shamaprasad, P., T.C. Moore, D. Xia, C.R. Iacovella, A.L. Bunge, and C. McCabe. 2022. Multiscale simulation of ternary stratum corneum lipid mixtures: effects of cholesterol composition. *Langmuir*. 38:7496–7511.

2. Glaser, J., T.D. Nguyen, J.A. Anderson, P. Lui, F. Spiga, J.A. Millan, D.C. Morse, and S.C. Glotzer. 2015. Strong scaling of general-purpose molecular dynamics simulations on GPUs. *Comput. Phys. Commun.* 192:97–107.

3. Moore, T.C., C.R. Iacovella, and C. McCabe. 2014. Derivation of coarse-grained potentials via multistate iterative Boltzmann inversion. *J. Chem. Phys.* 140:224104.

4. Moore, T.C., C.R. Iacovella, and C. McCabe. 2016. Development of a coarse-grained water forcefield via multistate iterative Boltzmann inversion. In: Snurr R, C Adjiman, D Kofke, editors. Foundations of Molecular Modeling and Simulation. Molecular Modeling and Simulation. Singapore: Springer. pp. 37–52.

5. Moore, T.C., C.R. Iacovella, R. Hartkamp, A.L. Bunge, and C. McCabe. 2016. A coarse-grained model of stratum corneum lipids: free fatty acids and ceramide NS. *J. Phys. Chem. B*. 120:9944–9958.

6. Moore, T.C., C.R. Iacovella, A.C. Leonhard, A.L. Bunge, and C. McCabe. 2018. Molecular dynamics simulations of stratum corneum lipid mixtures: A multiscale perspective. *Biochem. Biophys. Res. Commun.* 498:313–318.

7. Hoover, W.G. 1985. Canonical dynamics: Equilibrium phase-space distributions. *Phys. Rev. A*. 31:1695–1697.

8. Parrinello, M., and A. Rahman. 1981. Polymorphic transitions in single crystals: A new molecular dynamics method. *J. Appl. Phys.* 52:7182–7190.

9. Martyna, G.J., D.J. Tobias, and M.L. Klein. 1994. Constant pressure molecular dynamics algorithms. *J. Chem. Phys.* 101:4177–4189.

10. Cao, J., and G.J. Martyna. 1996. Adiabatic path integral molecular dynamics methods. II. Algorithms. *J. Chem. Phys.* 104:2028–2035.

11. Tuckerman, M.E., J. Alejandre, R. López-Rendón, A.L. Jochim, and G.J. Martyna. 2006. A Liouville-operator derived measure-preserving integrator for molecular dynamics simulations in the isothermal–isobaric ensemble. *J. Phys. Math. Gen.* 39:5629–5651.

12. Yu, T.-Q., J. Alejandre, R. López-Rendón, G.J. Martyna, and M.E. Tuckerman. 2010. Measure-preserving integrators for molecular dynamics in the isothermal–isobaric ensemble derived from the Liouville operator. *Chem. Phys.* 370:294–305.

13. Vermeer, L.S., B.L. de Groot, V. Réat, A. Milon, and J. Czaplicki. 2007. Acyl chain order parameter profiles in phospholipid bilayers: computation from molecular dynamics simulations and comparison with 2H NMR experiments. *Eur. Biophys. J.* 36:919–931.

14. McGibbon, R.T., K.A. Beauchamp, M.P. Harrigan, C. Klein, J.M. Swails, C.X. Hernández, C.R. Schwantes, L.-P. Wang, T.J. Lane, and V.S. Pande. 2015. MDTraj: a modern open library for the analysis of molecular dynamics trajectories. *Biophys. J.* 109:1528–1532.

15. Ramasubramani, V., B.D. Dice, E.S. Harper, M.P. Spellings, J.A. Anderson, and S.C. Glotzer. 2020. freud: A software suite for high throughput analysis of particle simulation data. *Comput. Phys. Commun.* 254:107275.

16. Harris, C.R., K.J. Millman, S.J. van der Walt, R. Gommers, P. Virtanen, D. Cournapeau, E. Wieser, J. Taylor, S. Berg, N.J. Smith, R. Kern, M. Picus, S. Hoyer, M.H. van Kerkwijk, M. Brett, A. Haldane, J.F. del Río, M. Wiebe, P. Peterson, P. Gérard-Marchant, K. Sheppard, T. Reddy, W. Weckesser, H. Abbasi, C. Gohlke, and T.E. Oliphant. 2020. Array programming with NumPy. *Nature*. 585:357–362.
