# Supplementary material for: The phase behavior of skin-barrier lipids: A combined approach of experiments and simulations
Source: Biophys J. 2024 Jul 18;123(18):3188–204. doi: 10.1016/j.bpj.2024.07.018 (PMC11447553; doi:10.1016/j.bpj.2024.07.018)
Supplement: Document S2. Article plus supporting material [file mmc3.pdf]

Biophysical Journal
Article

The phase behavior of skin-barrier lipids: A combined approach of experiments and simulations

Parashara Shamaprasad,[1] Andreea Nădăban,[2] Christopher R. Iacovella,[1] Gerrit S. Gooris,[2] Annette L. Bunge,[3] Joke A. Bouwstra,[2] and Clare McCabe[1,4,*]
[1]Department of Chemical and Biomolecular Engineering, Vanderbilt University, Nashville, Tennessee; [2]Division of BioTherapeutics, Leiden Academic Centre for Drug Research, Leiden University, Leiden, the Netherlands; [3]Department of Chemical and Biological Engineering, Colorado School of Mines, Golden, Colorado; and [4]School of Engineering and Physical Science, Heriot-Watt University, Edinburgh, United Kingdom

ABSTRACT Skin barrier function is localized in its outermost layer, the stratum corneum (SC), which is comprised of corneocyte cells embedded in an extracellular lipid matrix containing ceramides (CERs), cholesterol (CHOL), and free fatty acids (FFAs). The unique structure and composition of this lipid matrix are important for skin barrier function. In this study, experiments and molecular dynamics simulation were combined to investigate the structural properties and phase behavior of mixtures containing nonhydroxy sphingosine CER (CER NS), CHOL, and FFA. X-ray scattering for mixtures with varying CHOL levels revealed the presence of the 5.4 nm short periodicity phase in the presence of CHOL. Bilayers in coarse-grained multilayer simulations of the same compositions contained domains with thicknesses of approximately 5.3 and 5.8 nm that are associated with elevated levels, respectively, of CER sphingosine chains with CHOL, and CER acyl chains with FFA chains. The prevalence of the thicker domain increased with decreasing CHOL content. This might correspond to a phase with ~5.8 nm spacing observed by x-rays (other details unknown) in mixtures with lower CHOL content. Scissoring and stretching frequencies from Fourier transform infrared spectroscopy (FTIR) also indicate interaction between FFA and CER acyl chains and little interaction between CER acyl and CER sphingosine chains, which requires CER molecules to adopt a predominantly extended conformation. In the simulated systems, neighbor preferences of extended CER chains align more closely with the FTIR observations than those of CERs with hairpin ceramide chains. Both FTIR and atomistic simulations of reverse mapped multilayer membranes detect a hexagonal to fluid phase transition between 65 and 80°C. These results demonstrate the utility of a collaborative experimental and simulation effort in gaining a more comprehensive understanding of SC lipid membranes.

SIGNIFICANCE Skin's ability to act as a barrier to water and other chemicals lies primarily in the unique lipid structure in the stratum corneum. This study combined experiments and molecular dynamics (MD) simulations to explore the effect of cholesterol content on the lipid structure and arrangement of a model membrane containing equimolar nonhydroxy sphingosine ceramide (CER NS) and lignoceric acid. Lamellar phases, lipid arrangement, and thermotropic phase behavior were characterized by x-ray diffraction and Fourier transform infrared spectroscopy. Simulations of multilayer membranes with the same compositions as the experiments exhibit domain formations and phase transition temperatures that align with experimental observations. The study demonstrates the advantages of combining experiments and MD to gain a more comprehensive understanding of lipid membranes.

INTRODUCTION

The stratum corneum (SC), which is the outermost layer of the epidermis, plays a crucial role as the primary barrier preventing the entry of exogenous agents and loss of water through the skin. The SC is composed of corneocytes surrounded by an extracellular matrix of lipids. The lipids are primarily ceramides (CERs), which contain an acyl chain linked by an amide bond to a sphingoid base chain, cholesterol (CHOL), and free fatty acids (FFAs) in an approximately equimolar ratio (1). Within these lipid classes, over 20 CER subclasses with slightly altered headgroup and acyl tail structures have been observed in human SC (2). This diversity, combined with the chain length variations seen in the FFAs, yields a complex lipid mixture. X-ray diffraction studies of isolated SC from healthy volunteers, as well as membranes constructed from lipid extracts of isolated SC, reveal an ~13 nm long periodicity phase (LPP)

Submitted January 13, 2024, and accepted for publication July 15, 2024.
*Correspondence: c.mccabe@hw.ac.uk
Editor: Emma Sparr.
https://doi.org/10.1016/j.bpj.2024.07.018
© 2024 The Author(s). Published by Elsevier Inc. on behalf of Biophysical Society.
This is an open access article under the CC BY license (http://creativecommons.org/licenses/by/4.0/).

and an ~6 nm short periodicity phase (SPP) that frequently coexist with crystalline CHOL (3–7). Preparation of isolated SC does not affect the lipid organization in the SC (5).

There has been some controversy in the literature regarding the presence of the SPP and LPP in healthy SC stimulated by the work of Yagi et al. (8) reporting that the LPP alone could account for profiles obtained from x-ray diffraction. However, we note that Yagi et al. do not claim that the SPP does not exist. Indeed, a previous paper coauthored by Yagi (9) reports that the SPP and LPP disappear at different elevated temperatures and, therefore, do not belong to the same structure. Furthermore, in a recent paper from Narangifard et al. (10), both lamellar phases (repeat distances 5–6 and 11–12 nm for the SPP and LPP, respectively) have been reported in human SC based on cryoelectron microscopy. Finally, in x-ray diffraction studies of atopic eczema, and also dry skin, the missing third-order diffraction peak cannot be explained by a single lamellar phase (11), again supporting the presence of both the SPP and LPP.

Within the lamellae of the SPP and the LPP, the lipids can be organized in an orthorhombic (dense packing of lipids), hexagonal (ordered phase, but less densely packed), or liquid phase (disordered lipid chains). At physiological temperature, the human SC lipids primarily adopt an orthorhombic lateral packing, with a small proportion of the lipids adopting a hexagonal packing (12–14). Inflammatory skin diseases associated with a weakened skin barrier, such as atopic dermatitis and psoriasis, are also associated with changes in the lipid composition of the SC, resulting in altered lipid organization (15). To fully understand the role of lipids in the impaired skin barrier, the relationship between lipid composition, structure, and barrier properties must be investigated. In this paper we focus on the role of lipid composition on the structure of the lipid lamellae.

The SC lipid composition is complex and various changes in lipid composition often occur simultaneously. For this reason, studies of model lipid systems are important to systematically examine the effects of changes in composition of the SC lipids on lipid structure and barrier function. SC lipid model membranes with similar structure and permeability to native SC can be prepared using mixtures of synthetic or isolated CERs, along with CHOL and FFA (16–20). Studies using model membranes have shown that CHOL plays an important role in dictating the lamellar arrangement as well as the lateral packing in the SC (21–23). Specifically, in lipid models containing CER and FFA in an equimolar ratio, a small fraction of CHOL (1:0.2:1 molar ratio of CER/CHOL/FFA) is necessary for the formation of the SPP (21). Likewise, a small fraction of CHOL is required for the formation of the LPP. In addition, an increase in CHOL content promotes an orthorhombic lateral packing, like that observed in native SC. CHOL has also been found to impact the barrier properties in model lipid membranes. For example, the flux of water and two other small molecules, theophylline and indomethacin, decreased when the CHOL content decreased in an equimolar mixture of FFAs and CERs extracted from human SC (i.e., the CER/CHOL/FFA molar ratio changed from 1:1:1 to 1:0.4:1) (23).

While experimental studies have been the basis for several proposed molecular arrangements of the SC lipids, these arrangements are difficult to confirm using experimental methods alone. Molecular simulations can be a useful tool for validating experimental hypotheses and investigating structural characteristics inaccessible to experimental methods (24). However, until recently most molecular simulations of SC lipids have used atomistic models to examine single bilayers in water (25–27), which differ significantly from the experiments due to the presence of excess water on either side of the bilayer. Furthermore, atomistic models, where each atom is represented explicitly, lack the computational efficiency to feasibly simulate long-timescale events such as the self-assembly of SC lipid membranes (26). As a result atomistic simulations of multicomponent systems are typically initialized from preassembled configurations and cannot be assumed to be equilibrated structures (24).

More efficient coarse-grained (CG) models, in which several atoms are treated as a single interaction site (or bead), can be used to simulate the longer timescales required for self-assembly (28–32). In previous work, we describe CG models for nonhydroxy sphingosine CER (CER NS), FFAs (29,33), CHOL (34), and water (35) using the multistate iterative Boltzmann inversion method (MS-IBI) (36). We subsequently used these models to investigate the impact of CHOL content on self-assembled multilayer SPP structures of equimolar CER NS with an acyl chain of 24 carbon atoms (CER NS C24) and lignoceric acid (FFA C24) systems with varying levels of CHOL (34). In comparison with simulations of a single bilayer, the inner layers of the multilayer structures exhibited properties, such as bilayer thickness, that were more aligned with experimental results than those of the outer layers, presumably because the lipid-water interaction at the headgroup-water interface of the outer layers affects lipid packing. In addition, the absence of the headgroup-water interface in the inner layers allows the CERs to adopt an extended (linear) conformation with its two tails located in adjacent leaflets.

Although CG simulations are more efficient than atomistic simulations, atomistic-level precision is necessary to determine certain properties or behaviors, such as hydrogen bonding statistics and phase transition temperatures. When details from atomistic simulations are needed, reverse mapping can be used to convert CG configurations to atomistic resolution, enabling atomistic study of equilibrated systems that could not otherwise be reliably studied using atomistic simulation alone (34). Such a multiscale approach, where multilayer structures are self-assembled via CG simulations and reverse mapped to the atomistic level, utilizes the advantages of both the CG and atomistic resolutions.

In this paper, we adopted a combined experimental-simulation approach to gain a deeper understanding of the role of

CHOL in the structure of the SPP. Several articles have previously drawn parallels between experimental and simulated properties. For example, repeat distances from small-angle x-ray diffraction (SAXD) have been compared with simulated bilayer thicknesses (37); neutron scattering length density and electron density plots have been calculated from simulations and compared with their experimental analog (26,38,39); and lipid cross-sectional areas measured from simulations have been compared with those obtained using wide-angle x-ray diffraction and monolayer tension-area isotherms (26,34,40,41). However, publications in the simulation literature often describe systems that are unrealistic or impossible to translate to the experimental setting (24). For example, simulations often model 1) a single hydrated bilayer, in which the lipid headgroups contact essentially bulk water, rather than multilayers, in which the headgroups contact only a small amount of water (as observed in experiments), or 2) homogeneous (single phase) structures with lipid compositions that experimentally separate into two or more phases. Similarly, many experimental publications are ignored by simulation scientists because the systems are too complex to simulate, or the results cannot be readily validated via simulations (e.g., orthorhombic-hexagonal phase transitions, or measurements, such as Fourier-transform infrared spectroscopy [FTIR] or NMR spectra, which cannot be easily compared with simulations). A collaborative approach ensures that the interpretation and discussion of the results are relevant to both experimental and simulation audiences (42–44).

In this work the effect of CHOL content on the structure and arrangement of a simple SPP model system containing CER NS C24, CHOL, and FFA C24 has been investigated in both experiments and simulations. The CER/CHOL/FFA molar ratios used in this study (1:0:1, 1:0.05:1, 1:0.1:1, 1:0.2:1, 1:0.5:1, and 1:1:1) match those used by Mojumdar et al. in their investigations of a 13-component lipid mixture containing five CERs, CHOL, and a mixture of seven FFAs (21). As concluded by Mojumdar et al., crystallization of CHOL is initiated at a CER/CHOL/FFA molar ratio of 1:0.5:1, and at CHOL compositions between a 1:0.5:1 and 1:1:1 molar ratio, the amount of phase-separated CHOL increases without any change in the lamellar phases (21). Thus, CHOL compositions between 1:0.5:1 and 1:1:1 CER/CHOL/FFA are not included in this study. The three-component SPP model used here was chosen to reduce complexity for simulations of self-assembled systems, and to enable direct comparisons of the experimental results with the simulations and with those from Mojumdar et al. (21). First, the changes in lamellar phase behavior (i.e., phase separation) were characterized experimentally at various levels of CHOL content using SAXD. For comparison, the CG simulations with compositions matching the experiments were analyzed for separate domains exhibiting distinctive local bilayer thicknesses and compositions. Next, in experiments, the conformational ordering, lipid chain interactions, and thermotropic behavior of the CER NS C24/CHOL/FFA C24 mixture that forms only the SPP without phase-separated CHOL (1:0.5:1 molar ratio) were examined using FTIR. Complementary simulation results were derived for the same lipid composition from thermotropic studies using atomistic simulations of reverse mapped CG multilayers and a nearest neighbor analysis of the simulated CG multilayer structures performed to quantify the in-plane lipid morphology.

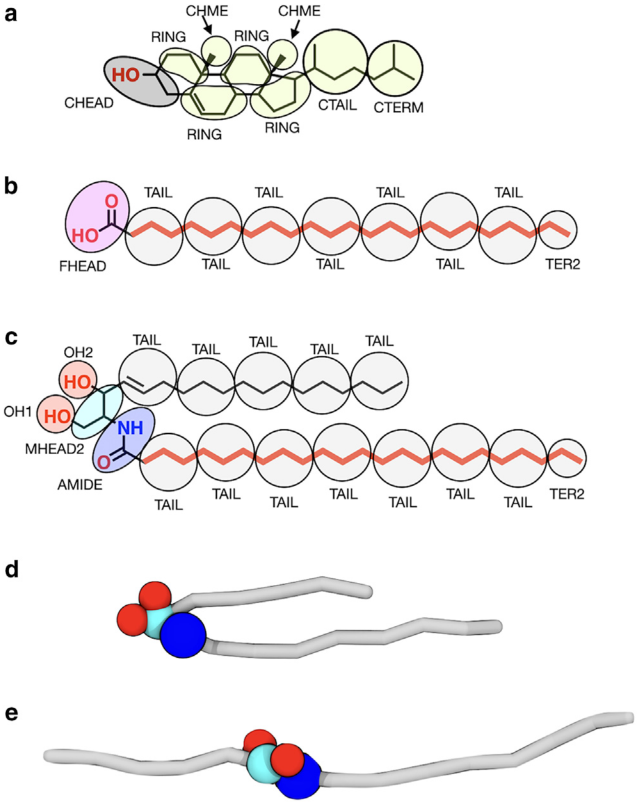

FIGURE 1 Molecular structures and CG mapping schemes for (*a*) CHOL, (*b*) FFA C24, and (*c*) CER NS C24. The carbon atoms bound to the perdeuterated FFA C24 (DFFA C24) and CER NS C24 (CER NSd47) are highlighted in red in the respective skeletal structures. CG representations of CER NS C24 in the (*d*) hairpin conformation and (*e*) extended (linear) conformation. Adapted with permission from Shamaprasad et al. (34) Copyright 2022 American Chemical Society.

MATERIALS AND METHODS

The experimental and simulations methods and analyses performed for each system is summarized in Table S1. Structures of the molecules studied, and the CG mapping schemes used in this study are depicted in Fig. 1.

Experimental materials and methods

Materials

CER NS with an acyl chain of C24 and a sphingosine chain of C18 (CER NS C24) and CER NS C24 with the perdeuterated acyl chain (d47), referred to as CER NSd47, were donated by Evonik (Essen, Germany). CHOL, lignoceric acid (FFA C24), acetate buffer salts, and deuterium oxide (D_2O) were purchased from Sigma-Aldrich Chemie GmbH (Schnelldorf, Germany). Perdeuterated FFA C24 (DFFA C24) was supplied by Arc Laboratories BV

(Apeldoorn, the Netherlands). All organic solvents were HPLC grade, supplied by Biosolve BV (Valkenswaard, the Netherlands). The Nuclepore polycarbonate filter disks (0.05 μm pore size) were purchased from Whatman (Kent, United Kingdom).

Lipid models preparation

One milligram of lipids was dissolved at a concentration of 4.5 mg/mL in a chloroform/methanol solution (2:1, v/v) for the FTIR studies and hexane/ethanol (2:1, v/v) for the SAXD measurements. The lipids were sprayed on the sample support (silver bromide window for FTIR, Nuclepore membrane for SAXD) using a Camag Linomat IV device (Muttenz, Switzerland) under a gentle stream of nitrogen with a spraying time of 14 s/μL. The lipid models were equilibrated using an automatic temperature equilibrator with a heating/cooling rate of 4°C/min, from 25 to 85°C, maintained at this temperature for 50 min, then cooled to 25°C. For the FTIR studies, the samples were hydrated $\geq$ 12 h using acetate buffer (pH 5) in D_2O, while for SAXD studies, the samples were maintained at 80% relative humidity (at least 24 h before the measurement).

FTIR measurements

A Varian 670 IR spectrometer (Agilent Technologies, Santa Clara, CA), with a mercury cadmium telluride detector, cooled by liquid nitrogen, was used. The FTIR spectra collection was performed in transmission mode by the co-addition of 256 scans collected over 4 min, with an instrument resolution of 1 cm^{-1}, in the wavenumber range 600–4000 cm^{-1}. Before each measurement, the samples were maintained for 30 min in a dry-air environment. The thermotropic behavior was measured between 10 and 90°C, with a 1°C temperature increase per recorded spectrum. The Resolution Pro (Agilent Technologies) software was used for both data collection and analysis, and the spectra were deconvoluted using a 1.4 enhancement factor and a 4 cm^{-1} halfwidth. The samples were prepared and measured in triplicate.

The CH_2 symmetric stretching vibration (ν_sCH_2, wavenumber range: 2845–2855 cm^{-1}) and CD_2 symmetric stretching vibration (ν_sCD_2, 2080–2100 cm^{-1}) were investigated to determine the phase transitions and the conformational ordering of the lipid chains. The interaction of the lipid chains was analyzed using the CH_2 and CD_2 scissoring vibration (δCH_2, 1462–1473 cm^{-1}; δCD_2, 1085–1095 cm^{-1}). The δCH_2 and δCD_2 peak positions were determined using in-house developed Python scripts.

SAXD measurements

The SAXD measurements were performed at the NCD-SWEET beamline, at ALBA Synchrotron (Barcelona, Spain). A Pilatus 1M detector with a pixel array of 981 × 1043 (each pixel of 172 × 172 μm^2) was used for these measurements and the x-ray beam wavelength was 0.9999 Å. The lipid models were measured for 20 s (at 23°C) and the distance between the sample and the detector was 2.148 m. Before the measurements, the set-up was calibrated with silver behenate. Each composition was measured in duplicate.

The one-dimensional x-ray intensity profiles of the scattering intensity (I) as a function of the scattering vector (q) were obtained after the integration of the two-dimensional scattering plot over a 90° segment from the beam center. The peak position (q_n) of each diffraction peak was determined by peak fitting using a Pearson VII function in the Fityk software. The repeat distances (d) of the lamellar phases were calculated as $d = 2n\pi / q_n$, where n represents the order number of the diffraction peaks of a lamellar phase. For the peaks that are not part of a lamellar structure and represent unknown phases, the spacing at position q was calculated as spacing $= 2\pi / q$.

Simulation methods

Simulation procedure

The CG simulations used the multistate iterative Boltzmann inversion-derived (MS-IBI) force fields for CER NS, CHOL, and FFA (29,33–36). Trajectories of self-assembled three-bilayer (six leaflets) CER NS C24/CHOL/FFA C24 multilayers at 31.85°C (305 K) were taken from Shamaprasad et al. (34). All systems contained 2000 lipid molecules and 20,000 CG water beads (i.e., 40 water molecules per lipid). As described in Shamaprasad et al. (34), these CG configurations were obtained by placing lipids with randomized positions between two water layers, each containing half of the water beads, and self-assembling multilayer structures during 1.6 μs of simulation using the shape-annealing protocol described in previous work and implemented in HOOMD-Blue 2.9.7 (45).

Atomistic temperature sweep simulations were performed on configurations derived from reverse mapping the self-assembled CG three-bilayer configuration for the CER NS C24/CHOL/FFA C24 mixture with a 1:0.5:1 molar ratio. The initial atomistic configuration, taken from Shamaprasad et al. (34), was generated using a reverse mapping approach that relies on the CER conformation (i.e., extended or hairpin; see Fig. 1, *d* and *e*), lipid in-plane structure, and average lipid tilt angle from the self-assembled CG configuration. Two water layers containing 13,333 water molecules were added adjacent to each of the outer leaflets (26,666 water molecules in total). This is approximately equivalent to 40 water molecules per lipid in the outer leaflets, given that roughly 1/6 of the 2000 lipids in the system are located in each of the outer leaflets. The water molecules located in the central four leaflets of the CG structure were not included in the reverse mapped structures to avoid high energy atomic overlaps, as in previous work (34). The reverse mapped structures were relaxed at 31.85°C (305 K) and 1 atm using the GROMACS simulation engine version 2018 (46) implementing the CHARMM36 force field (47) with head group parameters from the work of Guo et al. (41). Thermotropic behavior was examined by linearly heating from 31.85 to 126.85°C (305–400 K) or cooling from 31.85 to 6.85°C (305–280 K) the system at 2.5°C per ns (simulations were also performed by heating at the same rate from the lowest temperature configuration (280 K) to 400 K, which avoids discontinuous simulation results; see Fig. S10). Simulations were performed in the NPT ensemble with a constant pressure of 1 atm and a 1 fs timestep using the Nose-Hoover thermostat (48) and Parrinello-Rahman barostat (49) with semi-isotropic pressure coupling (x-y dimensions representing the lipid membrane plane are coupled).

Analyses

All analyses were performed on the CG systems, except for the thermotropic analyses, which were performed on the reverse mapped atomistic systems because CG models are unreliable at temperatures outside of those used in their optimization (24,50). For the CG systems, the final 200 ns (2000 frames collected every 0.1 ns) of each simulation were analyzed (where a frame refers to a single snapshot in time). For the atomistic system, the temperature-dependent time series was analyzed with block averaging (blocks of size 5°C). Analyses of CG and atomistic systems were performed making use of the MDTraj (51), Freud (52), NumPy (53), and Pandas (54) python packages.

Local bilayer thickness and composition. The final configurations of the self-assembled structures span 10–11 nm in the x and y dimensions. Local values of the bilayer thickness and composition were determined at points on a two-dimensional 50 × 50 grid drawn across the bilayer plane (i.e., the x-y plane). At each grid point, the lipids with a center of mass within 0.8 nm around the grid point in the x-y plane were isolated. The thickness of the isolated lipids around each grid point was calculated by creating a mass density histogram along the bilayer normal axis (z axis) and measuring the distance between the two innermost peaks, which correspond to the headgroups of the central bilayer (i.e., the two innermost leaflets). The composition of the lipids in the isolated portions of the central bilayer was determined by counting the number of each lipid tail type and dividing by the total number of lipid tails. Histograms of the thickness measurements and composition measurements were plotted by aggregating data for all of the grid points for all frames of the simulation. Values reported are pooled averages over the final 200 ns from these four simulations. In addition, local bilayer thicknesses and compositions for individual frames of the simulations were plotted as two-dimensional heatmaps using the *imshow* function of the *matplotlib* Python package (55).

Pearson and Spearman correlation coefficients between the local bilayer thickness and composition were calculated using the *scipy* package in Python (56). The Gaussian mixture clustering algorithm implemented in the *scipy* package was used to identify clusters in local composition versus thickness plots.

Nearest neighbor analysis. Lipid neighbor preferences were quantified by counting the number of neighboring lipid pairs of each type for the 1:0.5:1 CER NS C24/CHOL/FFA C24 system in the central bilayer of the three-bilayer stack. The number of neighboring pairs was calculated by first obtaining the centers of mass of each tail, where the CER NS acyl and sphingosine tails are counted separately (designated as CER NS acyl and CER NS sph, respectively) and identified as extended or hairpin depending on the CER conformation (Fig. 1, *d* and *e*). Tails are defined as the CG beads that are not headgroup beads (i.e., excluding the MHEAD2, AMIDE, OH1, and OH2 beads of CER NS, the HEAD bead of FFA, and the CHEAD bead of CHOL). Nearest neighbors are defined for each lipid tail as the 6 nearest chains by center of mass distance (7 for CHOL, as it has ~7 neighbors on average based on a Voronoi analysis (34)), which are identified using the Freud software package (52) in Python. Altogether, 6 types of lipid tails are considered (the acyl and sphingosine chains of extended and hairpin CER NS, CHOL, and FFA), which form 21 different lipid pairs (Table S4). The total number of each type of lipid tail pair in the central bilayer were counted for each frame and presented as the mean and standard deviation of the average over the final 200 ns (2000 frames) of 4 replicate simulations.

Thermotropic behavior. The thermotropic behavior of the reverse mapped atomistic simulations was characterized by changes in four different structural properties: normalized lipid area (NLA), thickness of the three-bilayer membrane, nematic order parameter (S2), and the carbon-hydrogen order parameter (S_{CH}).

The NLA is the sum of each lipid tail type in a given leaflet weighted by a normalizing factor, which accounts for their different cross-sectional areas (i.e., 1 for CER NS acyl, sphingosine, and FFA tails, and 1.9 for CHOL) divided by the total lateral area of the simulation box (34). The membrane thickness is the distance along the bilayer normal (z axis) between the water interfaces identified by the positions at which the bulk water density profile value drops by half.

The nematic order parameter (S2) is a global measure of the directional ordering of lipid tails, where a value of 1 describes a system in which all tails in a given leaflet point in the same direction and values close to zero describe systems with tails pointing in different directions (i.e., fully disordered) (24). For lamellar systems, S2 values between 0.8 and 1 indicate a well-ordered system (e.g., a hexagonal or orthorhombic phase), between 0.3 and 0.8 indicate a fluid (i.e., liquid crystalline membrane) phase, and less than 0.3 indicates an isotropic system. In this analysis, S2 was calculated by taking the largest eigenvector of the nematic tensor as described in the supporting information of Wilson (57) for a 12-carbon section of the tails (Fig. S1), selected to avoid artifacts associated with the interdigitated region between two leaflets and orientation of the tail near the headgroup.

The carbon-hydrogen order parameter (S_{CH}) was calculated as (24,58):

$$S_{CH} = \frac{1}{2}\langle 3\cos^2\theta - 1\rangle,$$

where θ is the angle between the C-H bond vector of carbon atoms in the individual lipid tails and the bilayer normal (i.e., the z axis). Values of S_{CH} range from −0.5, representing a lipid chain with the C-H bonds oriented perpendicular to the bilayer normal, to 1, indicating that C-H bonds are parallel to the bilayer normal (59).

RESULTS AND DISCUSSION

Influence of CHOL molar ratio on the lamellar organization of the lipid systems

The molar ratio of CHOL/CER NS was systematically increased from 0 to 1 while maintaining an equimolar mixture of CER NS and FFA C24. Fig. 2 shows the SAXD profiles of the six lipid models and in Table 1 the phases are described. In the absence of CHOL (Fig. 2 *A*), the SAXD profile shows two broad diffraction peaks (at q = 1.08 and 0.63 nm^{-1})

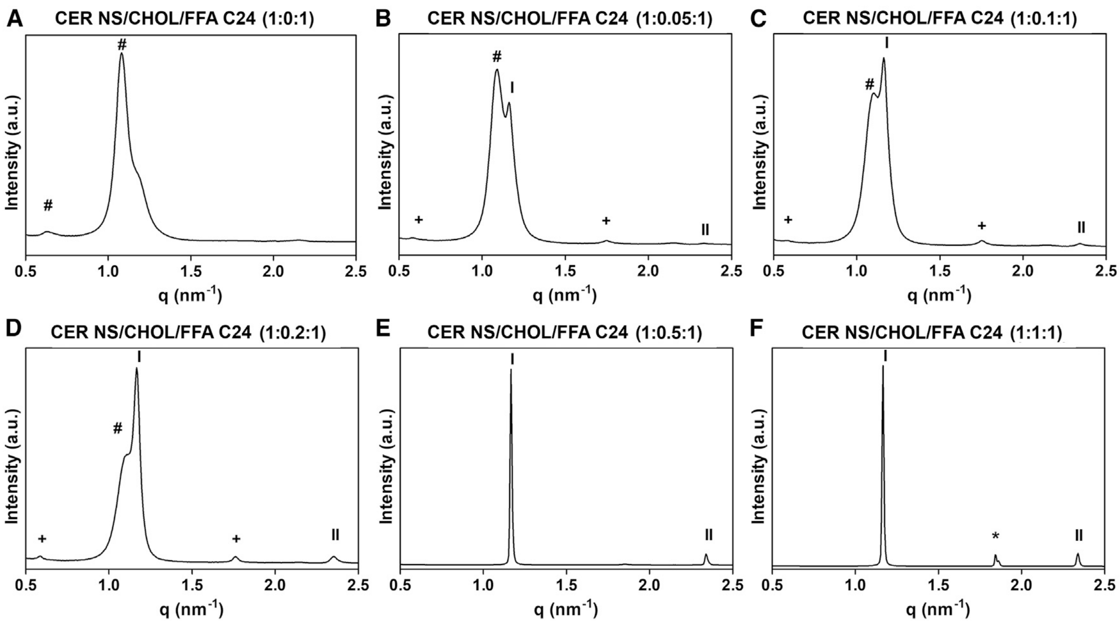

FIGURE 2 SAXD profiles of six lipid mixtures. The plot titles describe the composition (molar ratio) of each lipid system. The Roman numerals (I, II) indicate the diffraction orders attributed to the SPP; asterisk (*), crystalline CHOL with a reflection at q = 1.8 nm^{-1}; plus (+), the first and third diffraction order of a lamellar phase that has double the repeat distance of the SPP (~10.8 nm); hash (#), the peaks corresponding to unknown phases. The phases are described in Table 1.

TABLE 1 Studied lipid model compositions and the observed repeat distances of lamellar phases and spacings of unknown phases

Molar ratio of CER NS/CHOL/FFAC24	Phases formed	Repeat distances (nm)	Spacing (nm)
1:0:1	unknown phases	–	9.9, 5.8[b]
1:0.05:1	SPP, medium lamellar phase, and an unknown phase	10.5[a], 5.4	5.8[b]
1:0.1:1	SPP, medium lamellar phase, and an unknown phase	10.7[a], 5.4	5.7[b]
1:0.2:1	SPP, medium lamellar phase, and an unknown phase	10.8[a], 5.4	5.7[b]
1:0.5:1	SPP	5.4	
1:1:1	SPP and phase-separated CHOL	5.4	3.4[c]

Several phases have a spacing that does not correspond with the SPP repeat distance of 5.4 nm.
[a]Lamellar phase with a repeat distance twice that of the SPP.
[b]Diffraction peak of an unknown coexisting phase.
[c]Phase-separated crystalline CHOL.

corresponding to unknown phases with spacings of 5.8 and 9.9 nm, respectively. The models with the CER NS/CHOL/FFA 24 ratios of 1:0.05:1, 1:0.1:1, and 1:0.2:1 are characterized by sharp peaks with broad shoulders. The latter correspond to the spacings of 5.8 nm (1:0.05:1) and 5.7 nm (1:0.1:1 and 1:0.2:1). Apart from these shoulders, the first and second diffraction order of the SPP with the repeat distance of 5.4 nm can be observed in each of these models (Fig. 2, *B–D*, indicated by Roman numerals). When the CHOL concentration is increased to 0.5 (1:0.5:1 model, Fig. 2 *E*), the SPP is exclusively formed (repeat distance of 5.4 nm) and the SAXD plot displays two sharp peaks for the first and second diffraction orders of this lamellar phase. The 5.4 nm SPP (peak positions indicated by I and II in Fig. 2 *F*) and phase-separated crystalline CHOL (peak position at $q = 1.8\ nm^{-1}$, spacing 3.4 nm), is also formed in the equimolar ratio lipid model.

The models with a CHOL molar ratio between 0.05 and 0.2 formed another lamellar phase apart from the SPP, with a repeat distance of ∼10.8 nm, which is approximately double the length of a normal SPP (Fig. 2, *B–D*). The first and third diffraction orders of this phase are indicated in the figures by plus (+) signs (at $q \sim 0.58$ and $\sim 1.75\ nm^{-1}$, respectively), while the second and fourth diffraction order are overlapping with the SPP first and second order. This lamellar phase was reported previously as a "medium-lamellar phase" in lipid models that included CER NS C24 or CER NH C24 together or alone, in combination with CHOL, cholesterol sulfate, and FFA (60–63).

The SAXD results indicate that the molar ratio of CHOL in a lipid model has an important effect on the lamellar organization and the formation of the SPP. The SPP can be exclusively formed (apart from the crystalline CHOL phase) only at CHOL molar ratios of 0.5 and above (1:0.5:1 and 1:1:1 models), while at lower CHOL ratios (1:0.2:1, 1:0.1:1, 1:0.05:1, 1:0:1), there are coexisting phases formed. The results are similar to those found in a more complex SPP model studied by Mojumdar et al. (21), although the SPP was formed exclusively (without other coexisting phases) at CHOL molar ratios as low as 0.2.

Simulations of three-bilayer systems with compositions matching those in the SAXD experiments (1:0:1, 1:0.2:1, 1:0.5:1, 1:1:1 CER/CHOL/FFA) were found, with increasing CHOL, to exhibit a decrease in the average bilayer thickness of the central bilayer (which does not have direct contact with bulk water, see Fig. S2) (34). In previous work, this reduction in thickness was attributed to the end-to-end length of CHOL, which is shorter than the FFA and CER acyl chain; thus, increasing CHOL content enables increased interdigitation between opposing leaflets (34). In this study, we examined the localized thickness of the central bilayer to determine if domains of different thicknesses might be present. Specifically, the plane of the central bilayer was subdivided into 50×50 grid points (see methods), and the bilayer thickness calculated for the lipids that appear in a 0.8 nm radius about each grid point. This analysis did reveal lateral domains of different bilayer thicknesses within the same bilayer for the 1:0.2:1 and 1:0.5:1 CER/CHOL/FFA molar ratios, but not for the 1:0:1 and 1:1:1 molar ratios (Fig. 3). Fig. 3, *B* and *C* show clearly delineated areas of larger and smaller bilayer thickness akin to phase-separated domains for the 1:0.2:1 and 1:0.5:1 molar ratios, whereas mixtures at the 1:0:1 and 1:1:1 molar ratios (Fig. 3, *A* and *D*) each exhibit a more homogeneous thickness that is similar, respectively, to the larger (∼6 nm) and smaller (∼5 nm) domains observed in the systems with 1:0.2:1 and 1:0.5:1 molar ratios.

In Fig. 4, the frequencies of local bilayer thicknesses from the four independent simulations were aggregated and represented as a histogram for each composition. While the localized thickness distributions of the extreme compositions (CHOL/CER ratios of 0 and 1) are unimodal, bimodal distributions are observed for intermediate CHOL fractions (CHOL/CER ratios of 0.2 and 0.5). This is consistent with domain formation in the simulated system, in which each peak in the localized thickness distribution plot corresponds to a distinct domain. In the system with a CHOL/CER ratio of 0.5, a single sharp peak corresponding to SPP at 5.4 nm is observed in the SAXD experiments, indicating that there is little variation in the spacing. However, in the simulations the local bilayer thickness distribution in Fig. 3 *C* and the

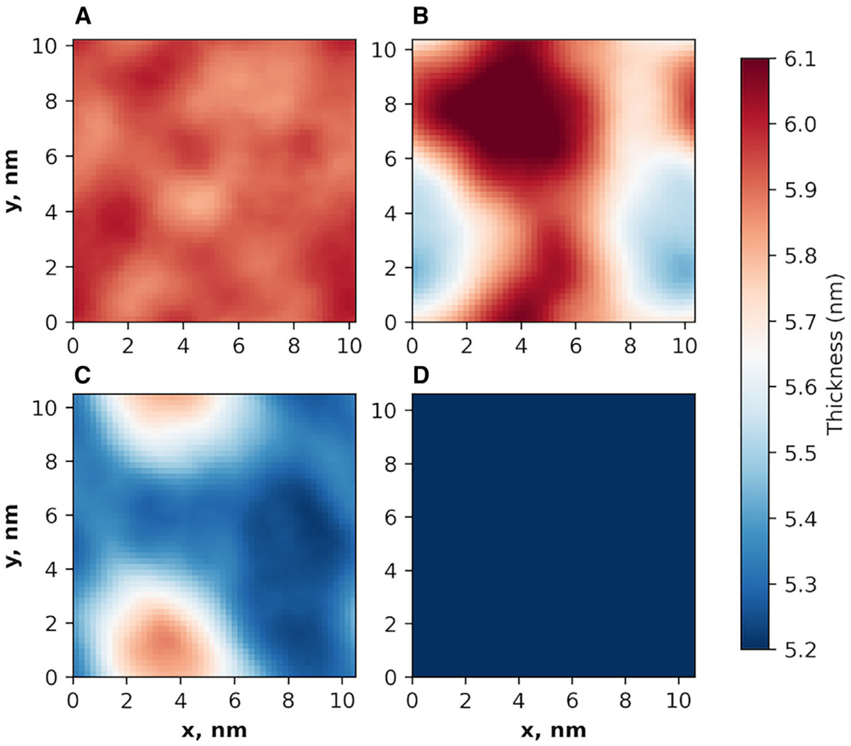

FIGURE 3 Plots of the local bilayer thickness distribution across the bilayer plane for the central bilayer from simulations of three stacked bilayers for the final frame of one simulation at each of four CER NS/CHOL/FFA molar ratios: (*A*) 1:0:1, (*B*) 1:0.2:1, (*C*) 1:0.5:1, and (*D*) 1:1:1.

bimodal peak in Fig. 4 indicate that two domains are present, although the domain with a 5.3 nm bilayer thickness dominates. In contrast, the 1:0.2:1 system, which was found to phase separate in SAXD experiments, has more equal peak heights in the thickness distribution plots, suggesting that the smaller and larger thickness domains are present in roughly similar amounts.

Interestingly, the system with a 1:1:1 molar ratio of CER, CHOL, and FFA has a narrower distribution of bilayer thicknesses than the system with no CHOL. A possible reason for this is that the 1:1:1 system has an equal number of long

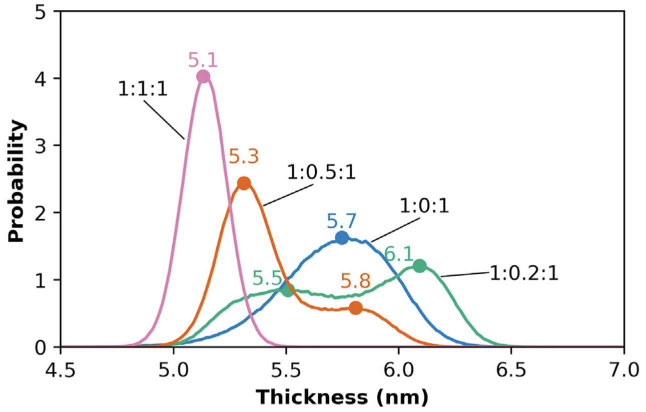

FIGURE 4 Histograms of the local bilayer thickness for the central bilayer of the three-bilayer systems with varying molar ratios of CHOL in an equimolar mixture of CER NS and FFA. The CER NS/CHOL/FFA molar ratios are indicated in black. Results are aggregated over four independent simulations at each composition.

(CER NS acyl and FFA) and short (CER NS sphingosine and CHOL) chains, which enables the short and long tails to organize across from each other and form a bilayer with a more uniform thickness over the bilayer plane. In the 1:0:1 system, however, there are twice as many long chains as short chains, meaning that long chains organize across from both short and long chains, which may cause the bilayer to have a more variable thickness over the bilayer plane.

The in-plane area of the CG simulations considered are small, only $\sim$10 $\times$ 10 nm, and may not be large enough to capture the domain sizes of phases observed in the experimental membranes. Furthermore, the repeat distances from x-ray diffraction are not the same as the central bilayer thickness from the three-bilayer simulated structures, which is not a repeating unit, although they should be comparable. Interestingly, despite these limitations, the peak locations for the simulations with the 0.2 and 0.5 CHOL/CER ratios are similar to the corresponding repeat distances of the main SAXD peaks: the respective shorter and longer domains are 5.3–5.5 and 5.8–6.1 nm from the simulations and 5.4 and 5.7–5.8 nm from SAXD.

We hypothesize that the multimodal distributions in bilayer thicknesses from the simulations are directly related to differences in composition, where domains with larger thicknesses have a higher concentration of long tail molecules (i.e., FFA and CER NS acyl) and those with smaller thicknesses have a higher concentration of short tail molecules (i.e., CHOL and CER NS sphingosine tails). Note that formation of these separate domains is only possible when at least some of the CER molecules are present in

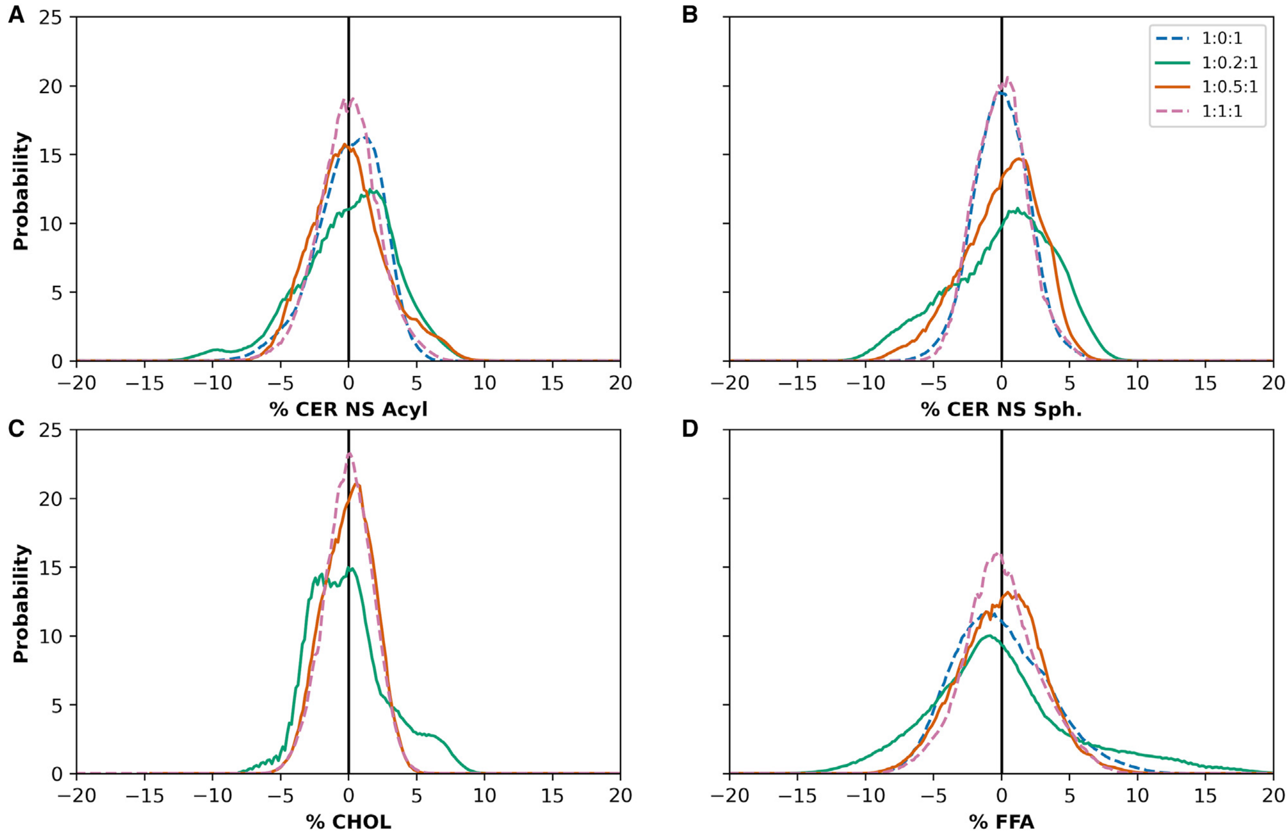

FIGURE 5 Histograms of the local excess fraction (positive number) or deficit fraction (negative number) of each tail type in the central bilayer of the three-bilayer system relative to the average fraction of each in lipid mixtures with equimolar CER NS and FFA and varying CHOL molar ratio: (*A*) CER NS acyl tails, (*B*) CER NS sphingosine tails, (*C*) CHOL, and (*D*) FFA. Acyl and sphingosine tails may have slightly different local fractions in the central bilayer because extended conformations of CER NS have only one tail in the central bilayer. The average tail fractions depend on the composition of the central bilayer. For example, the average fraction of each tail type is 0.25 in 1:1:1 CER/CHOL/FFA mixture, whereas for the 1:0.5:1 mixture, the average fraction of CHOL tails is 0.14, and 0.29 for each of the other tails. Results are aggregated over four independent simulations at each composition.

the extended conformation. The acyl and sphingosine chains of CERs in the hairpin position are forced to localize next to one another within a leaflet, thus prohibiting the formation of separate acyl or sphingosine rich domains. Approximately 35% of CERs in the central bilayer of the three-bilayer membrane are in the extended conformation (i.e., one CER tail in the central bilayer and the other in the adjacent leaflet) for the CER NS/CHOL/FFA mixtures with 1:0:1 and 1:0.5:1 molar ratios; slightly less (~32%) and slightly more (~38%) CERs are extended in the mixtures at 1:0.2:1 and 1:1:1, respectively (34). The effects of the hairpin CER conformation required in the outermost leaflets (those in contact with water), along with their compositions, which differ from the four inner leaflets, is unknown. Determination of the number of leaflets needed for the CER conformation, and the lipid composition and organization to become independent of the leaflet number is reserved for a future study.

To examine the hypothesis that local domains with different thicknesses have different composition, the local fraction of each tail type was measured in the same areas as the localized thickness analysis (see methods). Histograms of the local fraction in excess or deficit of the central bilayer composition for each lipid tail type reveal skewed or multimodal distributions for all tail types in the 1:0.2:1 system (Fig. 5). Also, the central bilayer compositions, provided in Table S2, differ slightly from the overall composition of the full system. This may indicate spatial segregation of domains with different concentrations. Histograms for the 1:0.5:1 system are also skewed, especially for the CER NS sphingosine tails, although to a lesser degree than the 1:0.2:1 mixture. This is consistent with results presented in Figs. 3 and 4, indicating that the 1:0.5:1 mixture exhibits less spatial segregation by bilayer thickness than the 1:0.2:1 system. The composition distributions for the 1:0:1 and 1:1:1 mixtures, however, adopt narrow, normal distributions, suggesting that the lipids are well-distributed throughout the bilayer (i.e., a single phase).

To quantify the relationship between local bilayer thickness and local composition of tail types in the central bilayer, Pearson correlation coefficients (r) for these quantities were calculated for each lipid tail type (Fig. 6), where $r = 0$ means no correlation, $|r| = 1$ means a perfect correlation, with the sign on r indicating either a negative

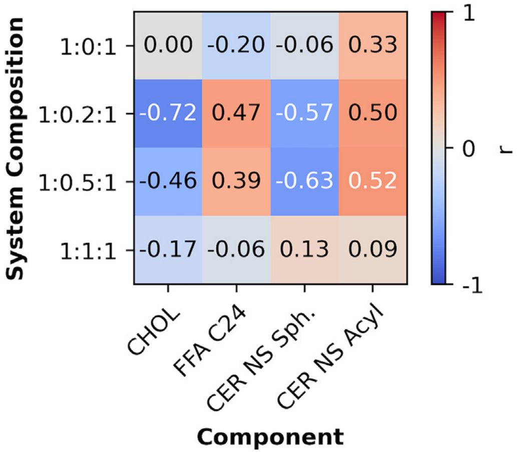

FIGURE 6 Pearson correlation coefficient (r) between the local fraction of each tail type and local bilayer thickness of the central bilayer in a three-bilayer system tabulated separately for CHOL, FFA C24, and the sphingosine and acyl tails of CER NS (*x* axis) for four mixtures at the indicated CER NS/CHOL/FFA molar ratios (*y* axis). Results are aggregated over four independent simulations at each composition. White text designates |r| > 0.5.

or positive correlation. Coefficients calculated for the Spearman correlation, which does not assume that a monotonic correlation is linear, are presented in Fig. S12; these are nearly identical to the Pearson correlation values. The correlations between the fraction of each tail type and the bilayer thickness are weak for the 1:0:1 and 1:1:1 ratios (|r| < 0.3 for all but the CER acyl tail of the 1:0:1 system), which is consistent with the absence of phase separation in the experiments. In contrast, systems with CHOL/CER ratios of 0.2 and 0.5 exhibit moderate correlations between the local fraction of each tail type and bilayer thickness (0.3 < |r| < 0.8). CHOL and CER NS sphingosine tail fractions correlate negatively with bilayer thickness, while FFA C24 and CER NS acyl tail fractions correlate positively. This indicates that the domain with the smaller bilayer thickness is, as hypothesized, associated with increased CHOL and sphingosine fractions, whereas the domain with the larger thickness is associated with increased FFA and acyl fractions. Note again that this is only possible because at least some of the CER molecules are present in the extended conformation.

The tail composition and the average bilayer thickness of domains with larger and smaller bilayer thicknesses were identified using the Gaussian mixture clustering algorithm (in Python) on scatterplots of the local tail composition versus the local bilayer thickness (Fig. S3). Table 2 lists the results for the central bilayer in the three-bilayer system with a 1:0.2:1 CER/CHOL/FFA molar ratio along with the average tail composition and bilayer thickness of the entire system. These results further support the conclusion from the Pearson correlation coefficient analysis that the domain with the smaller bilayer thickness is sphingosine and CHOL rich, whereas the domain with the larger bilayer thickness is acyl and FFA rich.

Discrepancies in the presence or absence of domains, their relative sizes, and their molar compositions in simulated and experimental structures may occur for several reasons, although the limited size of the simulated systems is likely an important contributor. While phase separation was not observed using SAXD of the 1:0.5:1 CER NS/CHOL/FFA mixture, the domain formation observed in the simulations of the same composition may not be unrealistic since domains of the size scale observed in the simulations are unlikely to be detected using SAXD. Unlike the experiments, phase-separated crystalline CHOL is not observed in simulations of the equimolar CER NS/CHOL/FFA mixture, nor are the ~10.7 nm phase and other unknown phases observed in small amounts in the SAXD profiles for equimolar CER and FFA with CHOL/CER molar ratios of 0.05–0.2. This may occur because the scale required for formation of more than one phase (e.g., crystalline CHOL and the SPP) exceeds, perhaps significantly, the ~10 × 10 nm in-plane area of the CG simulations, and/or because the quantity of the separate phase is small.

Differences in composition and the fraction of extended CERs may also contribute to discrepancies. The composition of the simulated structures can differ from those observed in experiments whenever separate phases that are detected in more than small amounts in the experiments are missing from simulations with the same nominal composition. For example, CHOL in the crystalline CHOL phase in the experiments would not be part of the 5.4 nm lamellar phase attributed to the SPP. Hence, the actual compositions of the SPP in the experiments with phase-separated CHOL will differ from the nominal (overall) equimolar composition that was used in the simulations. As a result, the simulation of the equimolar mixture, which exhibited a single homogenous phase, would have an excess of CHOL compared with the

TABLE 2 Average percent of each tail type in domains with larger and smaller bilayer thicknesses compared with the mixture average for the system with a 1:0.2:1 nominal molar ratio of CER NS/CHOL/FFA

	Average tail composition (%)				
Domain thickness	CER NS acyl	CER NS sph.	CHOL	FFA C24	Average bilayer thickness (nm)
Larger	34	27	4	34	5.78
Smaller	28	36	9	26	5.16
Mixture average	31	31	6	31	5.47

Results are aggregated over four independent simulations. sph., sphingosine.

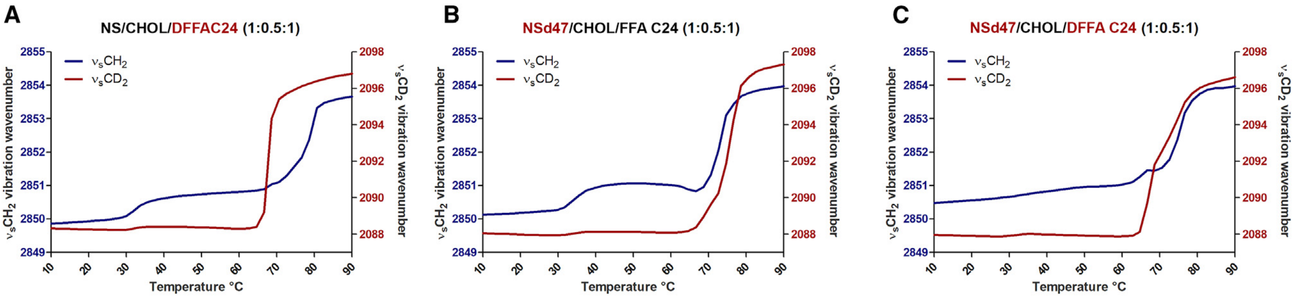

FIGURE 7 Thermotropic curves indicate the phase transitions of the lipids in the temperature interval 10–90°C for CER NS/CHOL/FFA at a 1:0.5:1 molar ratio with (*A*) perdeuterated FFA C24 chain (DFFA C24), (*B*) perdeuterated acyl chain of CER NS (NSd47), and (*C*) both CER NS acyl chain perdeuterated and the perdeuterated chain of the FFA C24. The wavenumber of the ν_sCH_2 peak position is shown on the left *y* axis (*blue*), the right *y* axis displays the wavenumber of the ν_sCD_2 peak position (*red*). The graphs represent an average of three measurements for each lipid model.

experimentally obtained SPP identified in the SAXD profile. This may explain the shorter bilayer thickness in the simulation of the equimolar mixture compared with the experimental repeat distance. With respect to the fraction of CERs in the extended conformation, ~35% of the CERs in the simulations (i.e., in the central bilayer of the three-bilayer system) are in the extended conformation (34), whereas in the experimental membranes CERs appear to be mostly extended (see Fig. 9 and the associated discussion below). Because formation of separate domains with larger and smaller bilayer thicknesses requires CERs to be in the extended conformation, this difference might also affect bilayer thickness.

Domain boundaries may also be affected by the edge of the small simulation box, altering the amount of each domain present in the system. Furthermore, domain formation may be sensitive to slight inaccuracies in the force field. Despite these issues, we demonstrate here that distinct domains are observed in the simulations of some lipid compositions, which might indicate that phase separation will occur in experiments of these compositions. If so, then the compositions of domains observed in the simulations might provide insight into the compositions of the phases observed in experiments, which are difficult to learn from experimental methods. Due to the short time to obtain results from simulations in comparison with sample preparation and measurements, simulations might be a useful tool to prescreen for possible phase separation or model phase boundaries across several compositions.

Thermotropic behavior of the SPP

The CER NS C24/CHOL/FFA C24 model with the molar ratio 1:0.5:1 was selected for further experimental analysis because this mixture exclusively forms the SPP, as concluded from the SAXD profiles (Fig. 2; Table 1). First, the conformational ordering of the lipids in the protiated model was examined. The ν_sCH_2 wavenumber at 10°C is 2848.8 ± 0.1 cm^{-1}, indicating a high conformational ordering of the lipids (Fig. S4 *B*). The wavenumber is constant until 32°C, when an increase to ~2850 cm^{-1} occurs, which demonstrates an increase in conformational disordering. The δCH_2 vibrations of this model are characterized by two sharp peaks at 10°C (Fig. S4 *A*). The δCH_2 peaks are fitted and the distance between the two peaks (δCH_2 peak splitting distance) amounts to 10.6 ± 0.05 cm^{-1}. The maximum splitting distance can be obtained for pure FFA C24 (10.7 cm^{-1}, not shown) which indicates large deuterated domains of ~100 lipid molecules (64,65). Therefore, at 10°C large orthorhombic lipid domains are present, and the transition observed in the ν_sCH_2 curves is the orthorhombic to hexagonal phase transition.

The lipid mixing behavior of the FFA C24 and CER NS C24 was studied using FTIR measurements with deuterated moieties included in the lipid models: the deuterated CER NS with a perdeuterated acyl chain (NSd47), the perdeuterated chain of the FFA C24 (DFFA C24), and a combination of both. The CH_2 and CD_2 lipid chain melting behaviors were examined by monitoring the peak position of the stretching vibrations (ν_sCH_2 and ν_sCD_2, Fig. 7 *A–C*). The ν_sCH_2 and ν_sCD_2 vibrations indicate that, between 10 and 32°C, the lipid chains of the CER NS/CHOL/DFFA C24 model (Fig. 7 *A*) were organized in an ordered phase (at 10°C: ν_sCH_2 wavenumber: 2849.9 ± 0.04 cm^{-1}; ν_sCD_2 wavenumber: 2088.3 ± 0.05 cm^{-1}), while between 32 and 36°C, an increase of the ν_sCH_2 peak wavenumber with ~0.7 cm^{-1} was observed, indicating an increase in conformational disordering. This is a transition from an orthorhombic to a hexagonal packing as indicated by the scissoring vibrations of the protiated mixture (Fig. S4 *B*). At higher temperatures, a sharp transition to a disordered liquid phase can be seen for the deuterated lipid chains. In this mixture DFFA C24 starts melting at a lower temperature range than the pure DFFA C24 (65°C compared with 84°C, unpublished data), suggesting that the DFFA chains interact with the protiated lipids (either CER NS or CHOL, or both). This is also indicated by the splitting of the CD_2 scissoring peaks of this model, which will be discussed below.

The thermotropic behavior of the CER NSd47/CHOL/FFA24 model (Fig. 7 *B*) is characterized by a conformational ordering of the lipids at 10°C (ν_sCH_2 wavenumber at 10°C: 2850.1 ± 0.1 cm^{-1}), followed by a transition characterized

by an increase in conformational disordering when the temperature reaches 32°C. This is caused by the orthorhombic to hexagonal phase transition. A reorganization of the protiated lipids occurs at 65°C (indicated by the decrease of the νCH_2 wavenumber by 0.5 cm^{-1}). An ordered-disorder transition starts at 67°C for both the protiated lipids and deuterated CER NS acyl chains, indicating melting over the same temperature range.

In Fig. 7 *C* the transitions based on the conformational disordering of the lipid system CER NSd47/CHOL/DFFAC24 are presented. At 10°C the deuterated lipid chains (acyl chain of CER NS and FFA) and the protiated lipids (sphingosine chain of CER NS and CHOL) are organized in an ordered phase, but the $\nu_s CH_2$ wavenumber at 10°C (2850.5 ± 0.04 cm^{-1}) is higher in comparison with the other models, indicating a higher conformational disordering of the protiated lipids in the CER NSd47/CHOL/DFFAC24 model. Moreover, there is no increase in conformational disordering at around 32°C for the protiated chains (Fig. 7 *C*, blue line), indicating that the sphingosine chains of CER NS and CHOL exhibit hexagonal packing. However, intermolecular coupling between the lipid chains can also increase the $\nu_s CH_2$ wavenumber in partly deuterated samples, but will not lead to a disappearance of the transition at around 32°C (66). A sharp increase in wavenumber of the deuterated lipids occurred between 66 and 79°C. The increase in wavenumber of the protiated chains occurs in the same temperature region, but the protiated chains first go through a small reordering, starting just after a small increase in wavenumber at 62°C.

Previous studies of the conformational ordering of CER NS C24/CHOL/FFA C24 system have indicated an extended conformation of CER NS C24 with a preference for its acyl tail to neighbor the FFA (67–69). More recently, using CER NS with either a perdeuterated sphingosine or acyl chain, Engberg et al. observed a lower conformational ordering of the sphingosine chain compared with the acyl chain in both FTIR and solid-state ^2H NMR measurements (69). Based on simulations of the NMR spectra performed at physiological temperatures, Engberg et al. proposed that the acyl chains formed an orthorhombic phase, whereas the sphingosine chains had a more disordered organization with most chains exhibiting a conformational order consistent with a gel (i.e., hexagonal) phase and 1/4 to 1/3 of the chains described as a fluid phase (69). However, it is important to note that the "fluid" terminology used in ^2H NMR and FTIR are not completely aligned. In FTIR, a fluid phase represents a higher disordering of the lipid chains as indicated by increased stretching vibration wavenumbers ($\nu_s CH_2$ wavenumber ~2854 cm^{-1}; $\nu_s CD_2$ wavenumber >2096 cm^{-1}), which Engberg et al. did not observe in their FTIR measurements. Therefore, the conclusion of this study that the sphingosine chains have less conformational ordering than the acyl chains of CER NS and are primarily hexagonally packed is in agreement with the data reported by Engberg et al. (69).

Thermotropic behavior was also explored via simulation by inspecting structural properties of the 1:0.5:1 molar ratio mixture of CER NS C24/CHOL/FFA C24 as a function of temperature using an atomistically detailed model derived from the CG self-assembled structures. Atomistic simulations are necessary for modeling thermotropic behavior since the CG model was optimized only for simulations at physiological skin temperature (i.e., ~32°C) and was not designed to be temperature transferrable (i.e., accurate at other temperatures). To be consistent with the temperature range in the experimental measurements (Fig. 7), the simulation results in Fig. 8 are reported between 11.85 and 91.85°C (285–365 K), with the full range investigated (up to 126.85°C, 400 K) included in the supporting material (Figs. S5–S9).

Fig. 8 *A–C* show, respectively, the average S2 values of the 12-carbon section (Fig. S1) of the FFA and CER acyl and

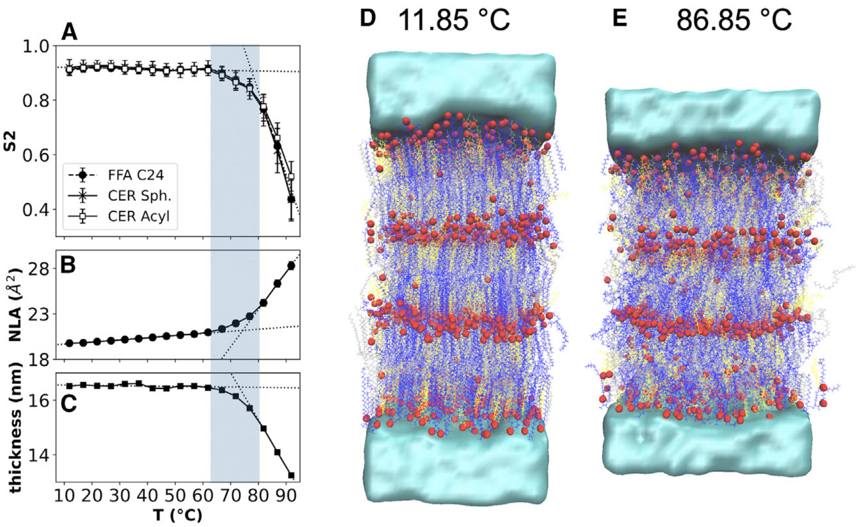

FIGURE 8 Temperature variation of structural properties calculated from atomistic simulations of the reverse mapped three-bilayer membrane containing CER NS C24/CHOL/FFA C24 with a 1:0.5:1 molar ratio: (*A*) nematic order parameter (S2) calculated separately for the FFA, and the acyl and sphingosine chains of CER NS, (*B*) normalized lipid area (NLA), and (*C*) total membrane thickness. The gray shading designates the estimated 65–80°C range of the hexagonal-fluid phase transition. Snapshots from simulations at temperatures below (*D*) and above (*E*) the hexagonal-fluid phase transition at the same scale. CER lipids are shown in silver, FFA in blue, and CHOL in yellow; oxygen atoms are rendered as red spheres to highlight layers; water is shown as a density isosurface. A movie constructed from simulation snapshots as a function of temperature is included in the supporting material (Video S1).

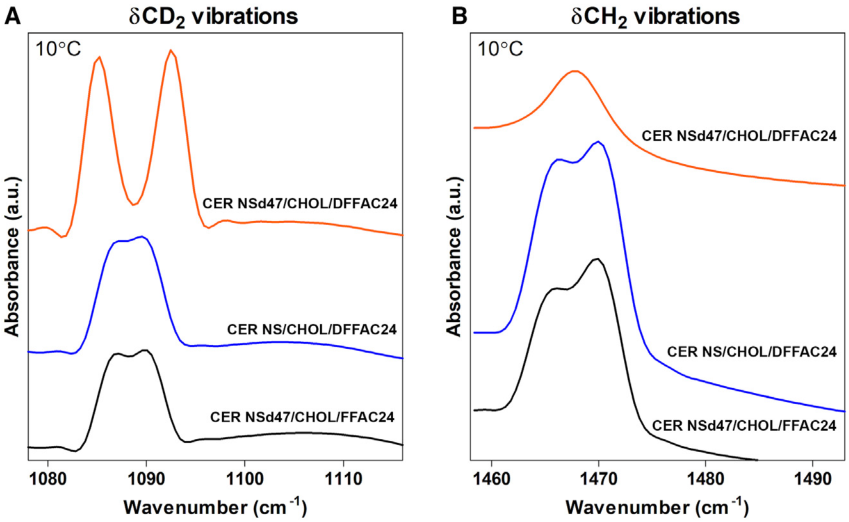

FIGURE 9 (*A*) δCD_2 and (*B*) δCH_2 vibrations for the CER NS/CHOL/FFA C24 (1:0.5:1) system with: deuterated NS acyl chain (*black bottom line*), deuterated FFA chains (*blue middle line*), and both deuterated NS acyl and FFA chains (*orange top line*). The vibrations shown are measured at 10°C. The peak splitting indicates the protiated and deuterated lipid chain interactions. The data presented are the average of three measurements for each model.

sphingosine chains in the four inner leaflets (i.e., all leaflets that are not in direct contact with bulk water), the NLA of the central bilayer, and the thickness of the entire three-bilayer membrane plotted as a function of temperature. Lines of best fit are included in each plot to highlight the two regimes observed in each of these metrics. The temperature changes in S2 clearly indicates a transition from a well-ordered phase at low temperature ($T < 65°C$, $S2 \sim 0.91$) to a fluid phase at $T \geq 80°C$ (where $S2 < 0.8$). Similar evidence of a hexagonal-to-fluid phase transition are also apparent in the behavior of the NLA and the membrane thickness. Both of these show a weak temperature dependence at lower temperature, with a stronger temperature dependence when $T \geq 65°C$. For $T \geq 65°C$, the area afforded to each lipid significantly increases, as typically occurs in transitions from a hexagonal to fluid phase membranes. This increase in NLA corresponds with increased lipid interdigitation and a consequent reduction in the overall membrane thickness.

Consistent with the structural analyses, snapshots from simulations show a significant loss of directional order and reduction in membrane thickness at 86.85°C compared with 11.85°C. Additional details of the thermotropic analyses are included in the supporting material, along with the calculated S_{CH} order parameter (Figs. S6–S9), which also support the observed hexagonal-fluid phase transition. All of the simulation results show little change in the structure between 11 and 65°C and a clear transition between roughly 65 and 80°C, which is consistent with the experimental results reported in Fig. 7. Our simulations and analysis suggest that this is the hexagonal-fluid phase transition observed in the experiments. In contrast with these results, bilayer simulations of the CER NS C24/CHOL/FFA C24 1:1:1 molar ratio mixture between water layers did not detect a phase transition even at temperatures as high as 400 K (126.85°C) (70), perhaps because the lipid-water interface stabilizes the single-bilayer structures. Additional analyses summarized in the supporting material (see Figs. S5–S9) suggest a further transition to an isotropic lipid phase occurs between approximately 95 and 105°C.

The experimentally observed orthorhombic to hexagonal phase transition between 32 and 36°C is not apparent in these structural properties, probably because these metrics are insufficiently sensitive to the small increase of the lipid packing in one direction and the rotational freedom around the axes of the lipid tails that distinguishes this phase transition (71).

Lipid chain interactions and lipid arrangement in the SPP

To further investigate the interactions of the lipid chains in the SPP forming an orthorhombic packing, the δCD_2 and δCH_2 frequencies were examined (Fig. 9). In partially deuterated lipid samples, the δCH_2 and δCD_2 vibrations of the hydrocarbon chains cannot directly interact due to the large energy difference between the δCH_2 and δCD_2 vibrations. As a result, when a deuterated chain is surrounded by protiated chains or vice versa, single peaks are observed in these models at $\delta CH_2 \sim 1470\ cm^{-1}$ and $\delta CD_2 \sim 1090\ cm^{-1}$. If the deuterated lipid chains are neighbors in large domains, the large number of CD_2-CD_2 interactions causes a δCD_2 peak splitting (i.e., there is distance between the two peaks). The maximum distance between the two δCD_2 peaks is around $7.4\ cm^{-1}$ (Fig. S11), which occurs when the lipid domains contain at least 100 chains (64,65); δCD_2 splitting distances less than $\sim 7.4\ cm^{-1}$ represent smaller deuterated lipid chain domain sizes. The intense peak splitting of the δCD_2 frequency in the CER NSd47/CHOL/DFFA C24 model (Fig. 9 *A*) therefore indicates strong CD_2-CD_2 interactions between the deuterated CER NS acyl and FFA lipid chains. Moreover, the distance between the two δCD_2 peaks corresponds to $7.4 \pm 0.05\ cm^{-1}$, which is also the peak split distance for pure DFFA C24 δCD_2 (data not shown),

indicating large domains of CD_2-CD_2 interactions. Thus, these results demonstrate that the FFA chains and the acyl chains of CER NS are neighboring in the SPP, while forming an orthorhombic phase. The deep dip between the two peaks demonstrates that there are almost no CD_2-CH_2 interactions, which would occur if there were interactions between the protonated CER sphingosine chains and either the deuterated CER acyl chains or the deuterated FFA chains.

If the CERs adopted a hairpin conformation, then the deuterated acyl chains would be neighboring the protiated sphingosine chains (Fig. S11) and no large δCD_2 peak splitting would occur. However, if almost all of the CERs adopt an extended conformation, then the deuterated acyl chains can be next to the deuterated FFA chains, which would produce a large δCD_2 peak splitting as observed. Therefore, the intense δCD_2 peak splitting in the CER NSd47/CHOL/DFFA C24 model can only occur if a large fraction of CER NS adopts an extended conformation, with the acyl and sphingosine chains on either side of the CER headgroup. The extended conformation of CER NS was previously reported in SPP models (67,69), as well as LPP models (72–74).

When both the acyl chain of CER NS and the FFA C24 are deuterated in the CER NSd47/CHOL/DFFA C24 model, the δCH_2 vibrations display a single peak (Fig. 9 *B*). As discussed above, the deuterated FFA C24 and acyl chain of CER NS form a large deuterated domain, therefore the single peak is caused by the protiated lipids (sphingosine chain of CER NS and CHOL) adopting a hexagonal packing, confirming the results of the CH_2 stretching vibration displayed in Fig. 7 *C*.

To study this further, either the deuterated acyl chain of CER NS or the deuterated FFA C24 was replaced with their protiated counterparts in the CER NSd47/CHOL/FFA C24 and the CER NS/CHOL/DFFA C24 models. In both models there is a reduction in the δCD_2 vibration peak splitting and a shallower dip between the peaks in comparison with the fully deuterated CER NSd47/CHOL/DFFA C24 model (Fig. 9 *A*). This indicates that the deuterated domain size is reduced and more CH_2-CD_2 chain interactions occur, as there are protiated chains (either the acyl chain of CER NS or FFAs, depending on the model) neighboring the deuterated moieties. The δCH_2 vibrations are also partially overlapping, similar to the δCD_2 vibrations showing some CD_2-CH_2 interactions, demonstrating the existence of orthorhombic packing (Fig. 9 *B*). These results confirm that the FFA C24 and acyl chains of CER NS are neighboring, as this is the reason for the reduction of the δCD_2 vibration peak splitting, and the CERs are in an extended conformation.

The extended conformation of CERs has several benefits for maintaining the SC barrier function. Compared with the hairpin CER conformation, the extended arrangement reduces the cross sectional area per lipid molecule, enabling a denser packing of the hydrocarbon lipid chains within the lamellar structure, which enhances the barrier function (73,75). An extended CER conformation also links adjacent lipid layers at the polar (headgroup) interface, which decreases the permeability although the SC lipid matrix by discouraging the swelling within the SPP during hydration (75,76).

While vibrational spectra are difficult to determine using molecular simulation, information on lipid organization can be deduced from the three-dimensional arrangement of the lipid positions that simulation provides. A simple metric for quantifying lipid neighbor preferences is the count of each of the possible neighboring lipid chain pairs. In this analysis, the extended and hairpin conformations of the acyl and sphingosine chains of CER NS are treated separately. Altogether, these 4 types of CER NS chains combined with the FFA chain and CHOL give 21 different neighboring lipid pairs. Table S3 lists the total number of each of these 21 neighboring lipid tail pairs present in the central bilayer of the three-bilayer stack of CER NS C24/CHOL/FFA C24 at a 1:0.5:1 molar ratio. However, using these numbers to assess neighbor preferences (e.g., to answer the question does the sphingosine tail of CER NS prefer CHOL over FFA?) is confounded by the different numbers of the lipid tail types. For example, even if the sphingosine chain of CER NS prefers to neighbor CHOL over FFA, it is likely to pair with CHOL less frequently than with FFA because the CHOL/FFA molar ratio is ~0.5.

To account for the lipid tail composition, normalized counts of neighboring tail pairs were calculated by dividing the relative occurrence of a given lipid tail pair (i.e., the ratio of the counts for a lipid tail pair to the total counts for all 21 lipid tail pairs from Table S3) by the relative probability of that pair's formation if the system was perfectly mixed (see Table 3). Because all the normalized values are equal to 1 in a perfectly mixed system, preferred neighbors have normalized counts greater than 1, and lipid tail pairs with larger normalized counts are more likely to neighbor one another than pairs with smaller normalized counts. In a perfectly mixed system, the probability that a pair of different lipid tail types (e.g., CHOL with FFA) will form is equal to the product of the number of the two tail types; for a pair of the same lipid tail type (e.g., CHOL with CHOL) the probability of formation is $N(N-1)/2$ where N is the number of that lipid tail type. The relative probability of a given lipid tail pair is the probability of its formation divided by the sum of the formation probabilities for all 21 lipid tail pairs.

A key difference between the simulations and experiments is the fraction of extended CERs. The experimental FTIR data suggest that CERs are predominantly in an extended conformation, while in the simulations only ~35% of the CERs in the central bilayer of the three-bilayer system are extended (i.e., one CER tail in the central bilayer and the other in the adjacent leaflet) (34). Hairpin CERs tend to favor CER NS acyl-CER NS sphingosine neighbors

TABLE 3 A sorted list of the normalized counts of neighboring lipid tails for the central bilayer of a 1:0.5:1 CER NS C24/CHOL/FFA C24 three-bilayer system

Tail 1	Tail 2	Normalized count
FFA C24	CER NS sph. hairpin	0.74 ± 0.03
FFA C24	CER NS acyl hairpin	0.74 ± 0.05
CER NS acyl hairpin	CER NS acyl hairpin	0.74 ± 0.06
CER NS sph. hairpin	CER NS sph. hairpin	0.78 ± 0.04
CER NS acyl extended	CER NS sph. hairpin	0.84 ± 0.07
FFA C24	CER NS sph. extended	0.88 ± 0.05
CER NS sph. extended	CER NS sph. hairpin	0.91 ± 0.04
FFA C24	CER NS acyl extended	0.94 ± 0.04
CER NS acyl extended	CER NS acyl hairpin	0.98 ± 0.04
CHOL	CER NS acyl hairpin	0.98 ± 0.04
CER NS acyl hairpin	CER NS sph. extended	0.99 ± 0.06
CER NS acyl extended	CER NS sph. extended	1.00 ± 0.10
CHOL	CER NS acyl extended	1.02 ± 0.07
CHOL	CHOL	1.07 ± 0.07
CHOL	CER NS sph. hairpin	1.08 ± 0.06
CER NS sph. extended	CER NS sph. extended	1.12 ± 0.12
CHOL	CER NS sph. extended	1.14 ± 0.09
FFA C24	FFA C24	1.21 ± 0.06
CHOL	FFA C24	1.28 ± 0.03
CER NS acyl extended	CER NS acyl extended	1.30 ± 0.07
CER NS acyl hairpin	CER NS sph. hairpin	1.56 ± 0.06

The normalized values are the ratio of the observed relative occurrence of a given pair to the relative probability that the pair forms if the system was perfectly mixed. Preferred neighbors have normalized counts greater than 1, and lipid tail pairs with larger normalized counts are more likely to neighbor each other than pairs with smaller normalized counts. Values are presented as the mean and standard deviation of the average over the final 200 ns (2000 frames) of 4 replicate simulations. sph., sphingosine.

relative to other lipid pairs with the CER tails (normalized count = 1.56). The requirement that the acyl and sphingosine tails of hairpin CERs must neighbor one another may skew the counts for other molecules neighboring with hairpin CERs, such as FFA, since an FFA molecule neighboring a hairpin CER NS acyl would often be forced to also neighbor the sphingosine tail of the same molecule. This is the case for FFA molecules, which neighbor acyl and sphingosine tails of hairpin CERs equally (normalized counts = 0.74 for both).

In contrast, we can see that there are more acyl-acyl neighbors and FFA-acyl neighbors and fewer sphingosine-acyl neighbors for extended CERs compared with hairpin CERs. Thus, neighbor preferences of the extended CERs in the simulations behave more like the experimental results. For both hairpin and extended CERs, CHOL molecules are found to favor CER NS sphingosine tails over acyl tails, as observed experimentally. In the simulations, CHOL prefers to neighbor FFA tails over all others. This is the opposite of some previous experimental observations using NMR (69), which, like the experiments presented here, concluded that most CER NS molecules are in the extended conformation. In addition, from the S_{CH} order parameters in Figs. S6–S9, the acyl and sphingosine chains of CER exhibit similar order parameters and phase transition temperatures. This contrasts with findings from the NMR experiments, which showed lower ordering and phase transition temperatures for the sphingosine chains (69). The preferences in the simulation for more CHOL-FFA pairs than CHOL-CHOL or FFA-FFA pairs, and also for an equal number of FFA-acyl and FFA-sphingosine pairs, may be artifacts of the larger fraction of CER molecules in the hairpin configuration compared with experiments. In a mixture of mostly extended CERs, the more closely matched lengths of the CER sphingosine tail (C18) with CHOL and of the CER acyl tails (C24) with FFA C24 might drive a sphingosine-CHOL and acyl-FFA pairing, which would not occur in a system with mostly hairpin CERs.

Lipid organization within a bilayer can also be assessed using the coordination number for each of the six lipid tail types with the designated reference lipid tail (e.g., the coordination numbers for the extended and hairpin acyl and sphingosine tails of CER NS, CHOL, and FFA with CHOL as the reference lipid). The results of a coordination number analysis (normalized for lipid tail composition), provided in the supporting material (Tables S5 and S6), are in close agreement with those from the normalized lipid pair count (Table S7).

The CG simulations may favor hairpin CERs over extended CERs since the CG model of CER NS was optimized to primarily reproduce target structures that contained only hairpin CERs (except for the bulk fluid states) (29,33,35). Also, it is possible that the hairpin conformation required in the outer leaflets may influence to some degree the lipid composition and CER conformation in the inner leaflets. As a result, the fraction of hairpin CERs in the simulated CG system may be unrealistically high and cause discrepancies between the simulated and experimental lipid arrangements. Adjustments to the CG model may be necessary to obtain more realistic fractions of extended CERs. Nonetheless, the nearest neighbor analysis presented here demonstrates that extended CERs play a crucial role in dictating the lipid arrangement, and the effect of extended CERs must be examined more closely in future studies.

CONCLUSIONS

In this work, we investigated the structural and thermotropic behavior of CER NS C24/CHOL/FFA C24 mixtures using both experimental and simulation techniques. First, using SAXD, an increasing presence of a 5.8 nm phase and decreasing presence of the SPP (5.4 nm) was observed experimentally as CHOL content decreases. An analysis of local bilayer thickness across the central bilayer of a simulated three-bilayer stack revealed domains with smaller (~5.3 nm) and larger (~5.8 nm) thickness. The similarities in the simulated domain formation and experimental phase separation for these compositions suggest that domain formation in simulated membranes could be indicators of larger-scale phase separation in experimental systems.

Thermal phase behavior was also investigated for CER NS C24/CHOL/FFA C24 at a molar ratio of 1:0.5:1 using FTIR combined with simulations of reverse mapped multilayer systems. Both the FTIR and the central bilayer of the simulated system showed changes consistent with a hexagonal-to-fluid phase transition between ∼65 and 80°C. The phase transition temperature for the simulated multilayer systems presented in this work shows much closer agreement to the experimental results compared with those obtained from single-bilayer simulations in previous work (70). This is likely due to the influence of the water-lipid interface in single-bilayer systems, which stabilizes the lipid organization. Thus, analysis of multilayer structures, rather than single bilayers, is essential for obtaining accurate thermotropic phase behavior in simulations. Furthermore, the thermotropic behavior of multilayer systems could be used to predict the effect of temperature change on various lipid compositions and arrangements, including identification of transitions at physiologically relevant temperatures.

FTIR spectroscopy of the 1:0.5:1 system with deuterated CER NS and FFA was also used to investigate lipid in-plane morphology. Based on the lipid tail interactions observed from the δCD_2 vibrations in the FTIR spectra, FFA and CER NS acyl tails form large deuterated domains with predominantly orthorhombic packing, and the absence of CHOL and CER NS sphingosine tails. Based on this, CERs are likely to be in an extended conformation since this would be necessary to separate the CER tails into adjacent leaflets containing either more CHOL or more FFA molecules. From a nearest neighbor analysis of the simulations, CER acyl tails appear to favor FFA neighbors, with an increased preference by the acyl tails of extended CERs compared with hairpin CERs. The smaller fraction of extended CERs in the CG simulations (only 35% extended) compared with the FTIR experiments is likely the cause of discrepancies in the simulated and experimental in-plane arrangement. Further investigation into the role of extended CERs and their effect on the membrane structure is necessary to better understand the lipid arrangement in the SPP.

The combined experimental and simulation approach provides a more complete description of the membrane structure and behavior at both the micro- and nanoscales. Experimental and simulation methods alone provide limited structural characteristics. Experimental methods such as SAXD and FTIR provide accurate measures of repeat distances or peak splitting but are unable to provide a detailed and mechanistic representation of the lipid arrangement. In contrast, simulations provide structures with a molecular-level resolution, but are unable to feasibly capture events that occur on large time and length scales, such as phase separation, due to the computational costs. However, the combination of simulations and experiments enables insights into the experimental results and the nanoscale structure obtained from simulations, which cannot be gained from either alone. The collaboration between simulation and experiment produces a synergy that will help accelerate research into the structure and barrier properties of SC lipids.

SUPPORTING MATERIAL

Supporting material can be found online at https://doi.org/10.1016/j.bpj.2024.07.018.

AUTHOR CONTRIBUTIONS

C.M., J.A.B., G.S.G., C.R.I., and A.L.B. designed the research. P.S., A.N., G.S.G., and C.R.I. performed the research. All authors analyzed data and wrote the article.

ACKNOWLEDGMENTS

This work was supported by the National Institute of Arthritis and Musculoskeletal and Skin Diseases grant no. R01AR072679.

DECLARATION OF INTERESTS

The authors declare no competing interests.

SUPPORTING REFERENCES

References (77–80) appear in the supporting material.

REFERENCES

1. Weerheim, A., and M. Ponec. 2001. Determination of stratum corneum lipid profile by tape stripping in combination with high-performance thin-layer chromatography. *Arch. Dermatol. Res.* 293:191–199.
2. Kawana, M., M. Miyamoto, ..., A. Kihara. 2020. Comparative profiling and comprehensive quantification of stratum corneum ceramides in humans and mice by LC/MS/MS. *J. Lipid Res.* 61:884–895.
3. Bouwstra, J. A., G. S. Gooris, ..., M. Ponec. 1998. Role of ceramide 1 in the molecular organization of the stratum corneum lipids. *J. Lipid Res.* 39:186–196.
4. Bouwstra, J. A., G. S. Gooris, ..., M. Ponec. 2001. Phase behavior of lipid mixtures based on human ceramides: coexistence of crystalline and liquid phases. *J. Lipid Res.* 42:1759–1770.
5. Schreiner, V., S. Pfeiffer, ..., J. Bouwstra. 2000. Barrier Characteristics of Different Human Skin Types Investigated with X-Ray Diffraction, Lipid Analysis, and Electron Microscopy Imaging. *J. Invest. Dermatol.* 114:654–660.
6. Caussin, J., G. S. Gooris, ..., J. A. Bouwstra. 2008. Lipid organization in human and porcine stratum corneum differs widely, while lipid mixtures with porcine ceramides model human stratum corneum lipid organization very closely. *Biochim. Biophys. Acta.* 1778:1472–1482.
7. Bouwstra, J. A., G. S. Gooris, ..., W. Bras. 1992. Structure of human stratum corneum as a function of temperature and hydration: A wide-angle X-ray diffraction study. *Int. J. Pharm. X.* 84:205–216.
8. Yagi, N., K. Aoyama, and N. Ohta. 2020. Microbeam X-ray diffraction study of lipid structure in stratum corneum of human skin. *PLoS One.* 15:e0233131.
9. Hatta, I., N. Ohta, ..., N. Yagi. 2006. Coexistence of two domains in intercellular lipid matrix of stratum corneum. *Biochim. Biophys. Acta.* 1758:1830–1836.

10. Narangifard, A., C. L. Wennberg, …, L. Norlén. 2021. Molecular Reorganization during the Formation of the Human Skin Barrier Studied In Situ. *J. Invest. Dermatol.* 141:1243–1253.e6.
11. Bouwstra, J. A., A. Nădăban, …, G. S. Gooris. 2023. The skin barrier: An extraordinary interface with an exceptional lipid organization. *Prog. Lipid Res.* 92:101252.
12. Bouwstra, J., G. Gooris, and M. Ponec. 2002. The Lipid Organisation of the Skin Barrier: Liquid and Crystalline Domains Coexist in Lamellar Phases. *J. Biol. Phys.* 28:211–223.
13. Mendelsohn, R., M. E. Rerek, and D. J. Moore. 2000. Infrared spectroscopy and microscopic imaging of stratum corneum models and skin. *Phys. Chem. Chem. Phys.* 2:4651–4657.
14. Boncheva, M., F. Damien, and V. Normand. 2008. Molecular organization of the lipid matrix in intact Stratum corneum using ATR-FTIR spectroscopy. *Biochim. Biophys. Acta.* 1778:1344–1355.
15. Van Smeden, J., M. Janssens, …, J. A. Bouwstra. 2014. The important role of stratum corneum lipids for the cutaneous barrier function. *Biochim. Biophys. Acta.* 1841:295–313.
16. Bouwstra, J. A., G. S. Gooris, …, M. Ponec. 1996. Phase behavior of isolated skin lipids. *J. Lipid Res.* 37:999–1011.
17. Bouwstra, J. A., G. S. Gooris, …, M. Ponec. 2002. Phase behavior of stratum corneum lipid mixtures based on human ceramides: the role of natural and synthetic ceramide 1. *J. Invest. Dermatol.* 118:606–617.
18. de Jager, M. W., G. S. Gooris, …, J. A. Bouwstra. 2004. Modelling the stratum corneum lipid organisation with synthetic lipid mixtures: the importance of synthetic ceramide composition. *Biochim. Biophys. Acta.* 1664:132–140.
19. Groen, D., G. S. Gooris, and J. A. Bouwstra. 2009. New insights into the stratum corneum lipid organization by X-ray diffraction analysis. *Biophys. J.* 97:2242–2249.
20. Uche, L. E., G. S. Gooris, …, C. M. Beddoes. 2021. Increased levels of short-chain ceramides modify the lipid organization and reduce the lipid barrier of skin model membranes. *Langmuir.* 37:9478–9489.
21. Mojumdar, E. H., G. S. Gooris, and J. A. Bouwstra. 2015. Phase behavior of skin lipid mixtures: The effect of cholesterol on lipid organization. *Soft Matter.* 11:4326–4336.
22. Zbytovská, J., M. A. Kiselev, …, R. Neubert. 2008. Influence of cholesterol on the structure of stratum corneum lipid model membrane. *Colloids Surf. A Physicochem. Eng. Asp.* 328:90–99.
23. Sochorová, M., P. Audrlická, …, K. Vávrová. 2019. Permeability and microstructure of cholesterol-depleted skin lipid membranes and human stratum corneum. *J. Colloid Interface Sci.* 535:227–238.
24. Shamaprasad, P., C. O. Frame, …, C. McCabe. 2022. Using molecular simulation to understand the skin barrier. *Prog. Lipid Res.* 88:101184.
25. Das, C., M. G. Noro, and P. D. Olmsted. 2009. Simulation studies of stratum corneum lipid mixtures. *Biophys. J.* 97:1941–1951.
26. Moore, T. C., R. Hartkamp, …, C. McCabe. 2018. Effect of ceramide tail length on the structure of model stratum corneum lipid bilayers. *Biophys. J.* 114:113–125.
27. Wang, E., and J. B. Klauda. 2018. Simulations of pure ceramide and ternary lipid mixtures as simple interior stratum corneum models. *J. Phys. Chem. B.* 122:2757–2768.
28. Sovová, Ž., K. Berka, …, P. Jurečka. 2015. Coarse-grain simulations of skin ceramide NS with newly derived parameters clarify structure of melted phase. *J. Phys. Chem. B.* 119:3988–3998.
29. Moore, T. C., C. R. Iacovella, …, C. McCabe. 2018. Molecular dynamics simulations of stratum corneum lipid mixtures: A multiscale perspective. *Biochem. Biophys. Res. Commun.* 498:313–318.
30. Podewitz, M., Y. Wang, …, Z. Cournia. 2018. Phase diagram of a stratum corneum lipid mixture. *J. Phys. Chem. B.* 122:10505–10521.
31. MacDermaid, C. M., K. W. Hall, …, G. Fiorin. 2020. Coexistence of lipid phases stabilizes interstitial water in the outer layer of mammalian skin. *Biophys. J.* 118:1588–1601.
32. Hadley, K. R., and C. McCabe. 2012. A simulation study of the self-assembly of coarse-grained skin lipids. *Soft Matter.* 8:4802–4814.
33. Moore, T. C., C. R. Iacovella, …, C. McCabe. 2016. A coarse-grained model of stratum corneum lipids: free fatty acids and ceramide NS. *J. Phys. Chem. B.* 120:9944–9958.
34. Shamaprasad, P., T. C. Moore, …, C. McCabe. 2022. Multiscale simulation of ternary stratum corneum lipid mixtures: effects of cholesterol composition. *Langmuir.* 38:7496–7511.
35. Moore, T. C., C. R. Iacovella, and C. McCabe. 2016. Development of a coarse-grained water forcefield via multistate iterative Boltzmann inversion. *In* Foundations of Molecular Modeling and Simulation. Molecular Modeling and Simulation. R. Snurr, C. Adjiman, and D. Kofke, eds Springer, Singapore, pp. 37–52.
36. Moore, T. C., C. R. Iacovella, and C. McCabe. 2014. Derivation of coarse-grained potentials via multistate iterative Boltzmann inversion. *J. Chem. Phys.* 140:224104.
37. Gupta, R., B. S. Dwadasi, and B. Rai. 2016. Molecular dynamics simulation of skin lipids: effect of ceramide chain lengths on bilayer properties. *J. Phys. Chem. B.* 120:12536–12546.
38. Wang, E., and J. B. Klauda. 2019. Molecular structure of the long periodicity phase in the stratum corneum. *J. Am. Chem. Soc.* 141:16930–16943.
39. Wang, E., and J. B. Klauda. 2018. Models for the stratum corneum lipid matrix: effects of ceramide concentration, ceramide hydroxylation, and free fatty acid protonation. *J. Phys. Chem. B.* 122:11996–12008.
40. Notman, R., W. K. den Otter, …, J. Anwar. 2007. The permeability enhancing mechanism of DMSO in ceramide bilayers simulated by molecular dynamics. *Biophys. J.* 93:2056–2068.
41. Guo, S., T. C. Moore, …, C. McCabe. 2013. Simulation study of the structure and phase behavior of ceramide bilayers and the role of lipid headgroup chemistry. *J. Chem. Theor. Comput.* 9:5116–5126.
42. Schmitt, T., R. Gupta, …, R. H. H. Neubert. 2018. Impact of the ceramide subspecies on the nanostructure of stratum corneum lipids using neutron scattering and molecular dynamics simulations. Part I: impact of CER[NS]. *Chem. Phys. Lipids.* 214:58–68.
43. Badhe, Y., T. Schmitt, …, R. H. H. Neubert. 2022. Investigating the nanostructure of a CER[NP]/CER[AP]-based stratum corneum lipid matrix model: A combined neutron diffraction & molecular dynamics simulations approach. *Biochim. Biophys. Acta Biomembr.* 1864:184007.
44. Lundborg, M., A. Narangifard, …, L. Norlén. 2018. Human skin barrier structure and function analyzed by cryo-EM and molecular dynamics simulation. *J. Struct. Biol.* 203:149–161.
45. Glaser, J., T. D. Nguyen, …, S. C. Glotzer. 2015. Strong scaling of general-purpose molecular dynamics simulations on GPUs. *Comput. Phys. Commun.* 192:97–107.
46. Abraham, M. J., T. Murtola, …, E. Lindahl. 2015. GROMACS: High performance molecular simulations through multi-level parallelism from laptops to supercomputers. *SoftwareX.* 1–2:19–25.
47. Klauda, J. B., R. M. Venable, …, R. W. Pastor. 2010. Update of the CHARMM all-atom additive force field for lipids: validation on six lipid types. *J. Phys. Chem. B.* 114:7830–7843.
48. Hoover, W. G. 1985. Canonical dynamics: Equilibrium phase-space distributions. *Phys. Rev.* 31:1695–1697.
49. Parrinello, M., and A. Rahman. 1981. Polymorphic transitions in single crystals: A new molecular dynamics method. *J. Appl. Phys.* 52:7182–7190.
50. Noid, W. G., J.-W. Chu, …, H. C. Andersen. 2008. The multiscale coarse-graining method. I. A rigorous bridge between atomistic and coarse-grained models. *J. Chem. Phys.* 128:244114.
51. McGibbon, R. T., K. A. Beauchamp, …, V. S. Pande. 2015. MDTraj: a modern open library for the analysis of molecular dynamics trajectories. *Biophys. J.* 109:1528–1532.
52. Ramasubramani, V., B. D. Dice, …, S. C. Glotzer. 2020. freud: A software suite for high throughput analysis of particle simulation data. *Comput. Phys. Commun.* 254:107275.
53. Harris, C. R., K. J. Millman, …, T. E. Oliphant. 2020. Array programming with NumPy. *Nature.* 585:357–362.
54. The pandas development team. 2020. Pandas-Dev/Pandas: Pandas.

55. Hunter, J. D. 2007. Matplotlib: A 2D Graphics Environment. *Comput. Sci. Eng.* 9:90–95.
56. Virtanen, P., R. Gommers; …, SciPy 1.0 Contributors. 2020. SciPy 1.0: fundamental algorithms for scientific computing in Python. *Nat. Methods.* 17:261–272.
57. Wilson, M. R. 1996. Determination of order parameters in realistic atom-based models of liquid crystal systems. *J. Mol. Liq.* 68:23–31.
58. Piggot, T. J., J. R. Allison, …, J. W. Essex. 2017. On the calculation of acyl chain order parameters from lipid simulations. *J. Chem. Theor. Comput.* 13:5683–5696.
59. Vermeer, L. S., B. L. de Groot, …, J. Czaplicki. 2007. Acyl chain order parameter profiles in phospholipid bilayers: computation from molecular dynamics simulations and comparison with 2H NMR experiments. *Eur. Biophys. J.* 36:919–931.
60. Pullmannová, P., E. Ermakova, …, K. Vávrová. 2019. Long and very long lamellar phases in model stratum corneum lipid membranes. *J. Lipid Res.* 60:963–971.
61. Kováčik, A., A. Vogel, …, D. Huster. 2018. Probing the role of ceramide hydroxylation in skin barrier lipid models by 2H solid-state NMR spectroscopy and X-ray powder diffraction. *Biochim. Biophys. Acta Biomembr.* 1860:1162–1170.
62. Kováčik, A., M. Šilarová, …, K. Vávrová. 2017. Effects of 6-hydroxyceramides on the thermotropic phase behavior and permeability of model skin lipid membranes. *Langmuir.* 33:2890–2899.
63. Pullmannová, P., B. A. Čuříková-Kindlová, …, K. Vávrová. 2023. Polymorphism, Nanostructures, and Barrier Properties of Ceramide-Based Lipid Films. *ACS Omega.* 8:422–435.
64. Moore, D. J., M. E. Rerek, and R. Mendelsohn. 1997. Lipid Domains and Orthorhombic Phases in Model Stratum Corneum: Evidence from Fourier Transform Infrared Spectroscopy Studies. *Biochem. Biophys. Res. Commun.* 231:797–801.
65. Mendelsohn, R., and D. J. Moore. 1998. Vibrational spectroscopic studies of lipid domains in biomembranes and model systems. *Chem. Phys. Lipids.* 96:141–157.
66. Kodati, V. R., R. El-Jastimi, and M. Lafleur. 1994. Contribution of the Intermolecular Coupling and Librotorsional Mobility in the Methylene Stretching Modes in the Infrared Spectra of Acyl Chains. *J. Phys. Chem.* 98:12191–12197.
67. Školová, B., K. Hudská, …, K. Vávrová. 2014. Different phase behavior and packing of ceramides with long (C16) and very long (C24) acyls in model membranes: infrared spectroscopy using deuterated lipids. *J. Phys. Chem. B.* 118:10460–10470.
68. Pullmannová, P., L. Pavlíková, …, K. Vávrová. 2017. Permeability and microstructure of model stratum corneum lipid membranes containing ceramides with long (C16) and very long (C24) acyl chains. *Biophys. Chem.* 224:20–31.
69. Engberg, O., A. Kováčik, …, K. Vávrová. 2020. The sphingosine and acyl chains of ceramide [NS] show very different structure and dynamics that challenge our understanding of the skin barrier. *Angew. Chem. Int. Ed.* 59:17383–17387.
70. Paloncýová, M., K. Vávrová, …, K. Berka. 2015. Structural changes in ceramide bilayers rationalize increased permeation through stratum corneum models with shorter acyl tails. *J. Phys. Chem. B.* 119:9811–9819.
71. Pilgram, G. S. K., D. C. J. Vissers, …, J. A. Bouwstra. 2001. Aberrant lipid organization in stratum corneum of patients with atopic dermatitis and lamellar ichthyosis. *J. Invest. Dermatol.* 117:710–717.
72. Beddoes, C. M., G. S. Gooris, and J. A. Bouwstra. 2018. Preferential arrangement of lipids in the long-periodicity phase of a stratum corneum matrix model. *J. Lipid Res.* 59:2329–2338.
73. Beddoes, C. M., G. S. Gooris, …, J. A. Bouwstra. 2020. Arrangement of ceramides in the skin: sphingosine chains localize at a single position in stratum corneum lipid matrix models. *Langmuir.* 36:10270–10278.
74. Nădăban, A., G. S. Gooris, …, J. A. Bouwstra. 2022. Phytosphingosine ceramide mainly localizes in the central layer of the unique lamellar phase of skin lipid model systems. *J. Lipid Res.* 63:100258.
75. Vávrová, K., A. Kováčik, and L. Opálka. 2017. Ceramides in the skin barrier. *Eur. Pharmaceut. J.* 64:28–35.
76. Paz Ramos, A., G. Gooris, …, M. Lafleur. 2018. Evidence of hydrocarbon nanodrops in highly ordered stratum corneum model membranes. *J. Lipid Res.* 59:137–143.
77. Martyna, G. J., D. J. Tobias, and M. L. Klein. 1994. Constant pressure molecular dynamics algorithms. *J. Chem. Phys.* 101:4177–4189.
78. Cao, J., and G. J. Martyna. 1996. Adiabatic path integral molecular dynamics methods. II. Algorithms. *J. Chem. Phys.* 104:2028–2035.
79. Tuckerman, M. E., J. Alejandre, …, G. J. Martyna. 2006. A Liouville-operator derived measure-preserving integrator for molecular dynamics simulations in the isothermal–isobaric ensemble. *J. Phys. Math. Gen.* 39:5629–5651.
80. Yu, T.-Q., J. Alejandre, …, M. E. Tuckerman. 2010. Measure-preserving integrators for molecular dynamics in the isothermal–isobaric ensemble derived from the Liouville operator. *Chem. Phys.* 370:294–305.

Biophysical Journal, Volume *123*

Supplemental information

The phase behavior of skin-barrier lipids: A combined approach of experiments and simulations

Parashara Shamaprasad, Andreea Nădăban, Christopher R. Iacovella, Gerrit S. Gooris, Annette L. Bunge, Joke A. Bouwstra, and Clare McCabe

Supplemental Material

The phase behavior of skin-barrier lipids: a combined approach of experiments and simulations

Parashara Shamaprasad[a], Andreea Nădăban[b], Christopher R. Iacovella[a], Gerrit S. Gooris[b], Annette L. Bunge[c], Joke A. Bouwstra[b], Clare McCabe[a,d].

[a] Department of Chemical and Biomolecular Engineering, Vanderbilt University, Nashville, TN 37235-1604, United States of America
[b] Division of BioTherapeutics, Leiden Academic Centre for Drug Research, Leiden University, 2333 CC Leiden, the Netherlands
[c] Department of Chemical and Biological Engineering, Colorado School of Mines, Golden, CO 80401, United States of America
[d] School of Engineering and Physical Science, Heriot-Watt University, Edinburgh, United Kingdom

Corresponding Author: Clare M[c]Cabe
Corresponding Author Email: c.mccabe@hw.ac.uk

Contents:

Description of coarse-grained simulation methodology

Figure S1. Atomic schematics (carbons in green, oxygens in red, nitrogen in blue, and hydrogens in gray) of A) FFA C24 and B) CER NS C24 molecules showing the 12-carbon section in each chain (circled) that were used in the S2 calculations (Figures 8A and S5).

Figure S2. Snapshots from CG simulations of the 3 bilayer (6 leaflet) membranes of CER NS C24:CHOL:FFA C24 with molar ratios of (A) 1:0:1, (B) 1:0.2:1, (C) 1:0.5:1, (D) and 1:1:1.

Figure S3. Scatter plots of the local bilayer thickness versus local composition of each lipid chain in the central bilayer of 3-bilayer membranes containing a 1:0.2:1 molar ratio mixture of CER NS C24:CHOL:FFA C24.

Figure S4. The δCH_2 vibration at 10°C (A), and the thermotropic behavior (B) of the CER NS:CHOL: FFA24 (1:0.5:1) protiated model.

Figure S5. The density of the 3-bilayer membrane and the nematic order parameter (S2) for FFA C24, and the sphingosine and acyl chains of CER NS C24 in the central bilayer of a 3-bilayer membrane plotted as a function of temperature to show the hexagonal-to-fluid phase transition between 65°C and 80°C and the fluid-to-isotropic phase transition between 95°C and 105°C.

Figure S6. Average change in the S_{CH} order parameters for all carbons on the FFA C24 chain, and the sphingosine and acyl chains of CER NS C24 in the four inner leaflets of the 3-bilayer membrane at the specified temperature (T) relative to the lowest temperature simulated (285 K, 11.85ºC).

Figure S7. S_{CH} order parameter calculated for the FFA molecules in the four inner leaflets of the 3-bilayer membrane (i.e., all leaflets that are not in direct contact with bulk water).

Figure S8. S_{CH} order parameter calculated for the sphingosine chain of CER NS molecules in the four inner leaflets of the 3-bilayer membrane.

Figure S9. S_{CH} order parameter calculated for the acyl chain of CER NS molecules in the four inner leaflets of the 3-bilayer membrane.

Figure S10. Temperature variation of structural properties calculated from atomistic simulations of the reverse-mapped 3-bilayer membrane containing CER NS C24:CHOL:FFA C24 with a 1:0.5:1 molar ratio

Figure S11. Explanation of the FTIR CD_2 scissoring vibrations and a schematic representation of the CERs conformation.

Figure S12. Spearman correlation coefficient between the local fraction of each tail type and local bilayer thickness of the central bilayer in a 3-bilayer system

Table S1. Systems studied by composition and method used.

Table S2. Comparison of the molar ratios for each lipid tail type (normalized by the average number of CER NS sphingosine tails) and the total number of lipid tails in the central bilayer and two outer bilayers of a 3-bilayer membrane containing a 1:0.5:1 molar ratio mixture of CER NS C24:CHOL:FFA C24.

Table S3. Raw count of neighboring pairs of lipid tails for the central bilayer of a 3-bilayer membrane containing a 1:0.5:1 molar ratio mixture of CER NS C24:CHOL:FFA C24.

Table S4. Number of each lipid tail type in the central bilayer of a 3-bilayer membrane containing a 1:0.5:1 molar ratio mixture of CER NS C24:CHOL:FFA C24.

Table S5. Raw values of the coordination number representing the number of each lipid tail type (listed by column) that neighbor the reference lipid tail type (listed in the first column) in the central bilayer of a 3-bilayer membrane containing a 1:0.5:1 molar ratio mixture of CER NS C24:CHOL:FFA C24.

Table S6. Normalized coordination numbers representing the number of each of the six lipid tail types (listed by column) that neighbor the reference lipid tail type (listed in the first column) in the central bilayer of a 3-bilayer membrane containing a 1:0.5:1 molar ratio mixture of CER NS C24:CHOL:FFA C24.

Table S7. Normalized lipid tail pair count ratio from the nearest neighbor analysis (column 3), listed in descending order, compared with the fraction of each lipid tail type that neighbors the reference lipid tail calculated from the normalized coordination numbers.

Description of the coarse-grained simulation methodology

The coarse-grained simulation trajectories of the 3-bilayer simulations of CER NS C24:CHOL:FFA C24 in 1:0:1, 1:0.2:1, 1:0.5:1, and 1:1:1 molar ratios analyzed in this work were generated as described in Shamaprasad et al. (1). Simulations were initialized with a box of 2000 lipids with a density of 0.8 g/mL surrounded in the positive and negative z-dimensions by boxes of 10000 water beads with a density of 1.0 g/mL (20000 water beads in total). This is equivalent to 40 water molecules per lipid since the CG model maps 4 water molecules to each water bead.

All coarse-grained simulations used a 10 fs timestep and were run using the HOOMD-Blue 2.9.7 simulation engine (2). The coarse-grained force field was optimized using the multistate iterative Boltzmann inversion (MS IBI) method (3), in which both bulk fluid and lamellar states were used as targets for optimization (1, 4–6). The systems were first relaxed with a brief 20 ps simulation using the microcanonical (NVE) ensemble with a 0.1 Å limit on the displacement of each bead at every timestep to remove high energy overlaps. Next, a 10 ns simulation is performed in the isothermal-isobaric (NPT) ensemble at 305 K and 1 atm with an isotropic barostat to equilibrate the system density while keeping the aspect ratio of the box constant. The systems then undergo the shape annealing procedure at 305 K in the canonical (NVT) ensemble, in which the lateral area is expanded to double the expected final lateral area over 200 ns, compressed to the expected final lateral area over another 200 ns, and held constant for an additional 200 ns. The expected final lateral area for each system is set based on the expected average area per lipid, which is a function of the lipid composition. The simulations are then equilibrated using the NPT ensemble for 300 ns. To eliminate any defects in the structures, an additional temperature annealing step in the NVT ensemble is conducted, where the box dimensions are held constant and the temperature is increased from 305 K to 375 K over 50 ns, held at 375 K for 100 ns, and cooled to 305 K over 50 ns. The system is then equilibrated in the NPT ensemble at 305 K and 1 atm for 500 ns. In the last 200 ns of this final simulation, the trajectory is recorded every 0.1 ns to produce 2000 frames that were used in the data analyses of this paper. All simulations in the NVT ensemble use the Nosé-Hoover thermostat (7, 8) with a temperature coupling constant of 1 ps, and simulations in the NPT ensemble use the Martyna-Tobias-Klein barostat-thermostat (9–12) with temperature and pressure coupling constants of 1 ps and 10 ps, respectively.

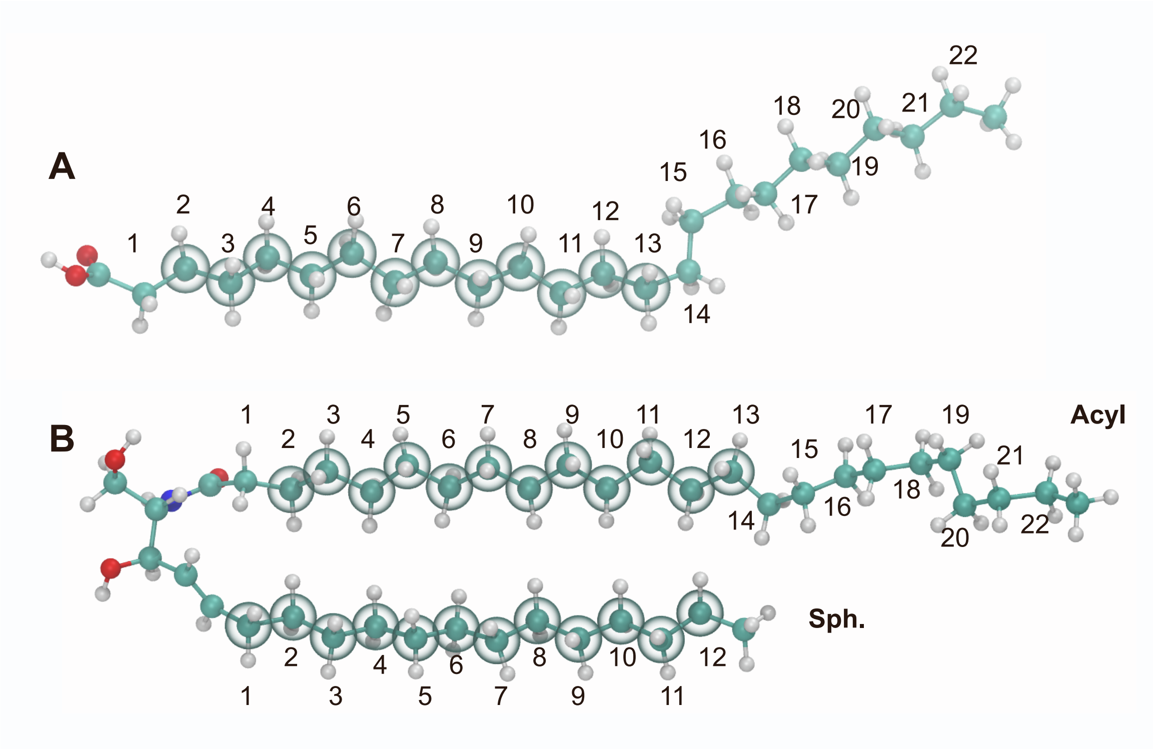

Figure S1. Atomic schematics (carbons in green, oxygens in red, nitrogen in blue, and hydrogens in gray) of A) FFA C24 and B) CER NS C24 molecules showing the 12-carbon section in each chain (circled) that were used in the S2 calculations (Figures 8A and S5) so that the director is calculated within regions that are well-ordered at low temperature (i.e., the ends of the chains, which tend to be disordered in the interdigitated regime, even at low temperature, are excluded). Numbers listed on each molecule correspond to the carbon numbers in plots of the S_{CH} shown in Figures S7 – S9.

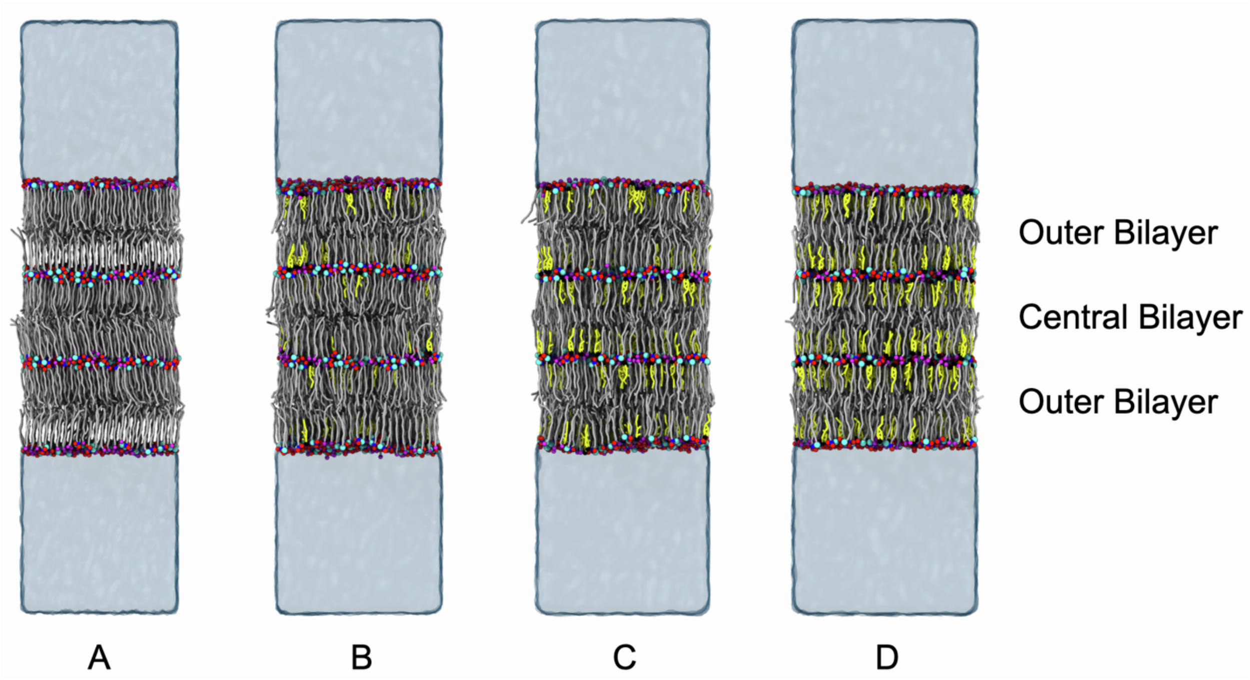

Figure S2. Snapshots from CG simulations of the 3 bilayer (6 leaflet) membranes of CER NS C24:CHOL:FFA C24 with molar ratios of (A) 1:0:1, (B) 1:0.2:1, (C) 1:0.5:1, (D) and 1:1:1. The central bilayer (consisting of the two innermost leaflets) is sandwiched between the two outer bilayers. Lipid backbones are represented as a stick model (CER and FFA tails in gray, and CHOL in yellow), with headgroup CG beads (as depicted in Figure 1 of the paper) rendered as spheres (MHEAD2, cyan; AMIDE, blue; OH1 and OH2, red; FHEAD, purple; and CHEAD, black). Reprinted with permission from Shamaprasad, et al. (1).

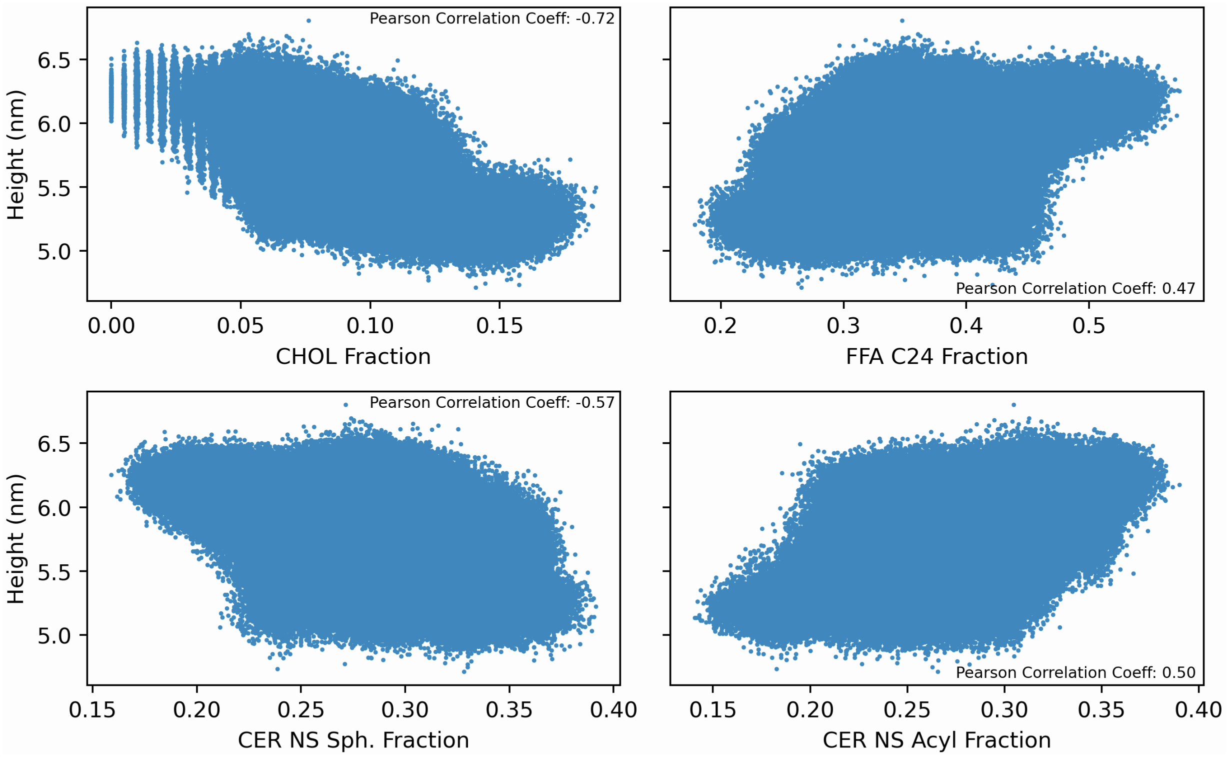

Figure S3. Scatter plots of the local bilayer thickness versus local composition of each lipid tail in the central bilayer of 3-bilayer membranes containing a 1:0.2:1 molar ratio mixture of CER NS C24:CHOL:FFA C24.

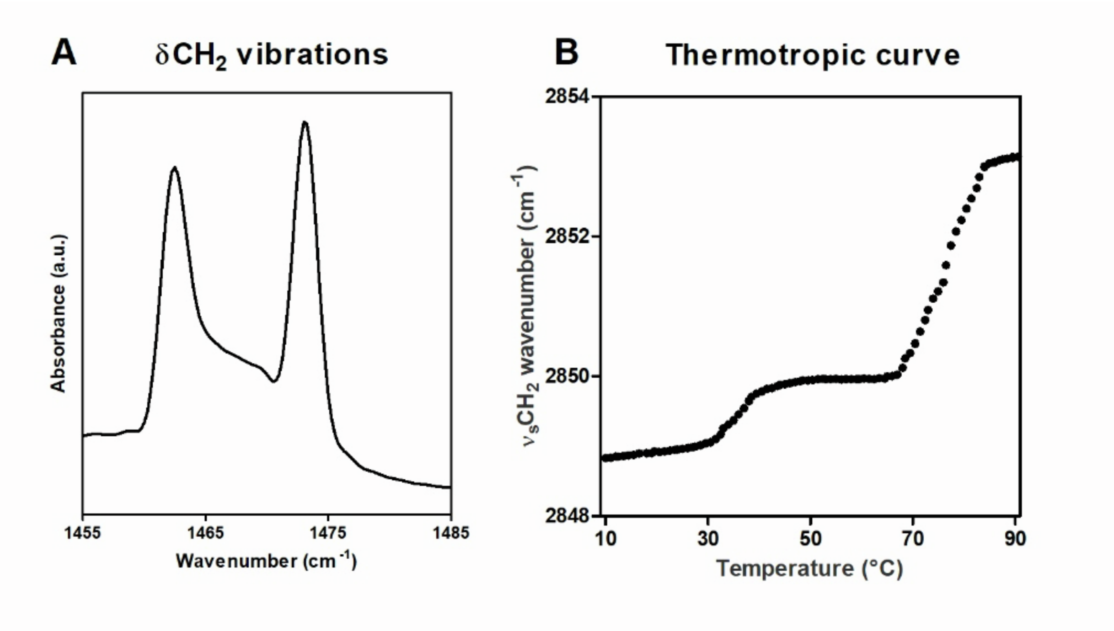

Figure S4. The δCH_2 vibration at 10°C (A), and the thermotropic behavior (B) of the CER NS:CHOL: FFA24 (1:0.5:1) protiated model. The thermotropic curve shows the $\nu_s CH_2$ wavenumbers as a function of temperature in the range 10-90°C.

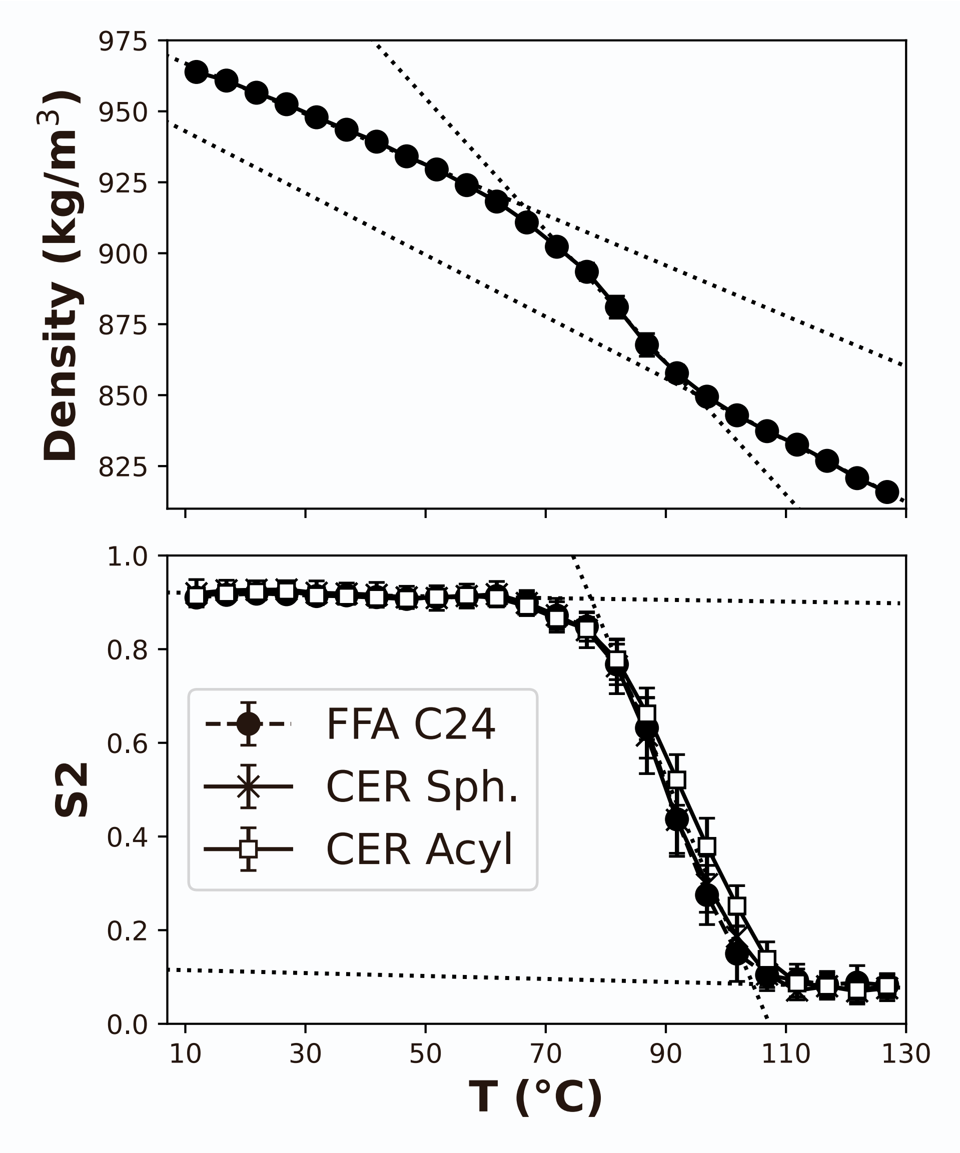

Figure S5. The density of the 3-bilayer membrane and the nematic order parameter (S2) for FFA C24, and the sphingosine and acyl chains of CER NS C24 in the central bilayer of a 3-bilayer membrane plotted as a function of temperature to show the hexagonal-to-fluid phase transition between 65°C and 80°C and the fluid-to-isotropic phase transition between 95°C and 105°C. Results presented here were calculated as described for those presented in Figure 8. Best-fit lines are included for the three regimes representing the hexagonal, fluid and isotropic phases. S2 values were calculated for the 12 carbon atoms designated in Figure S1 and reported as the mean and standard deviation of the average over the final 200 ns (2000 frames) of one simulation.

Descriptions of Figures S6 – S9

Figure S6 shows the average change in the S_{CH} order parameters (calculated as described in the Methods section) for all carbons on the FFA C24 chain and for sphingosine and acyl chains of CER NS C24 in the four central leaflets of the 3-bilayer membrane (i.e., all leaflets that are not in direct contact with bulk water) at the specified temperature relative to the lowest temperature simulated (285 K, 11.85ºC). Plots of the S_{CH} order parameters for each carbon in the FFA chain and in the sphingosine and acyl chains of the four central leaflets are presented in Figures S7, S8 and S9, respectively. Carbon numbers in Figures S7-S9 correspond to those shown in Figure S1. Values of S_{CH} range from –0.5 representing a lipid chain with the C-H bonds oriented perpendicular to the bilayer normal and a value of S_{CH} = 1 indicating C-H bonds are parallel to the bilayer normal (13).

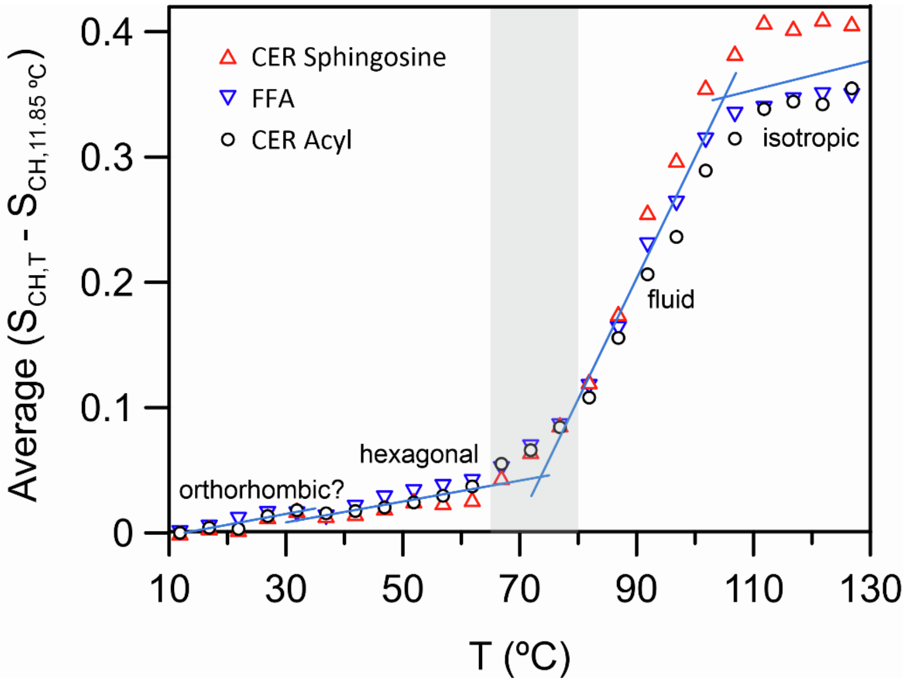

Figure S6. Average change in the S_{CH} order parameters for all carbons on the FFA C24 chain, and the sphingosine and acyl chains of CER NS C24 in the four inner leaflets of the 3-bilayer membrane at the specified temperature (T) relative to the lowest temperature simulated (285 K, 11.85ºC). The gray shading designates the estimated 65°C to 80°C range of the hexagonal-fluid phase transition.

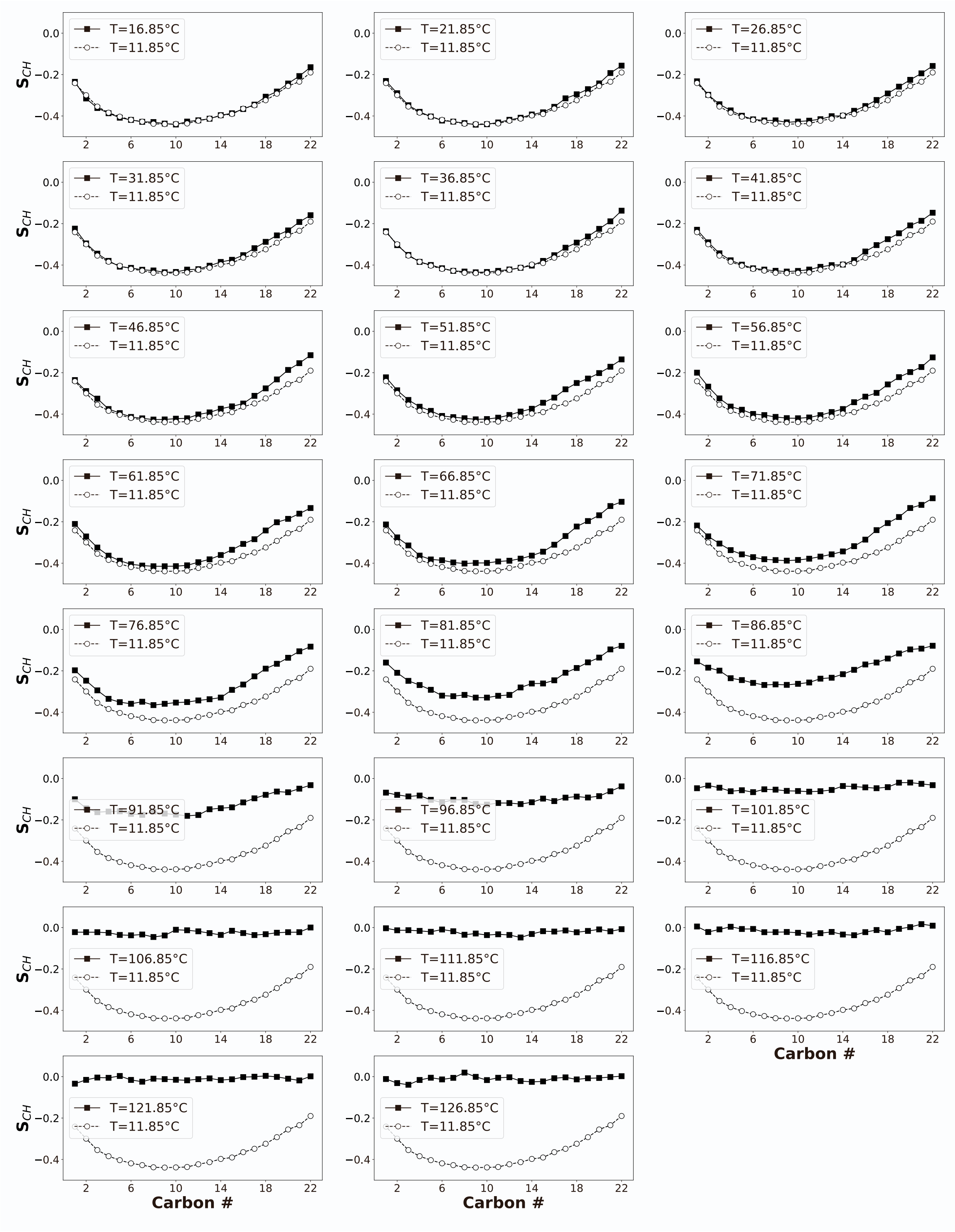

Figure S7. S_{CH} order parameter calculated for the FFA molecules in the four inner leaflets of the 3-bilayer membrane (i.e., all leaflets that are not in direct contact with bulk water) calculated at the designated temperatures as described in the Methods section. Carbon numbers correspond to those shown in Figure S1.

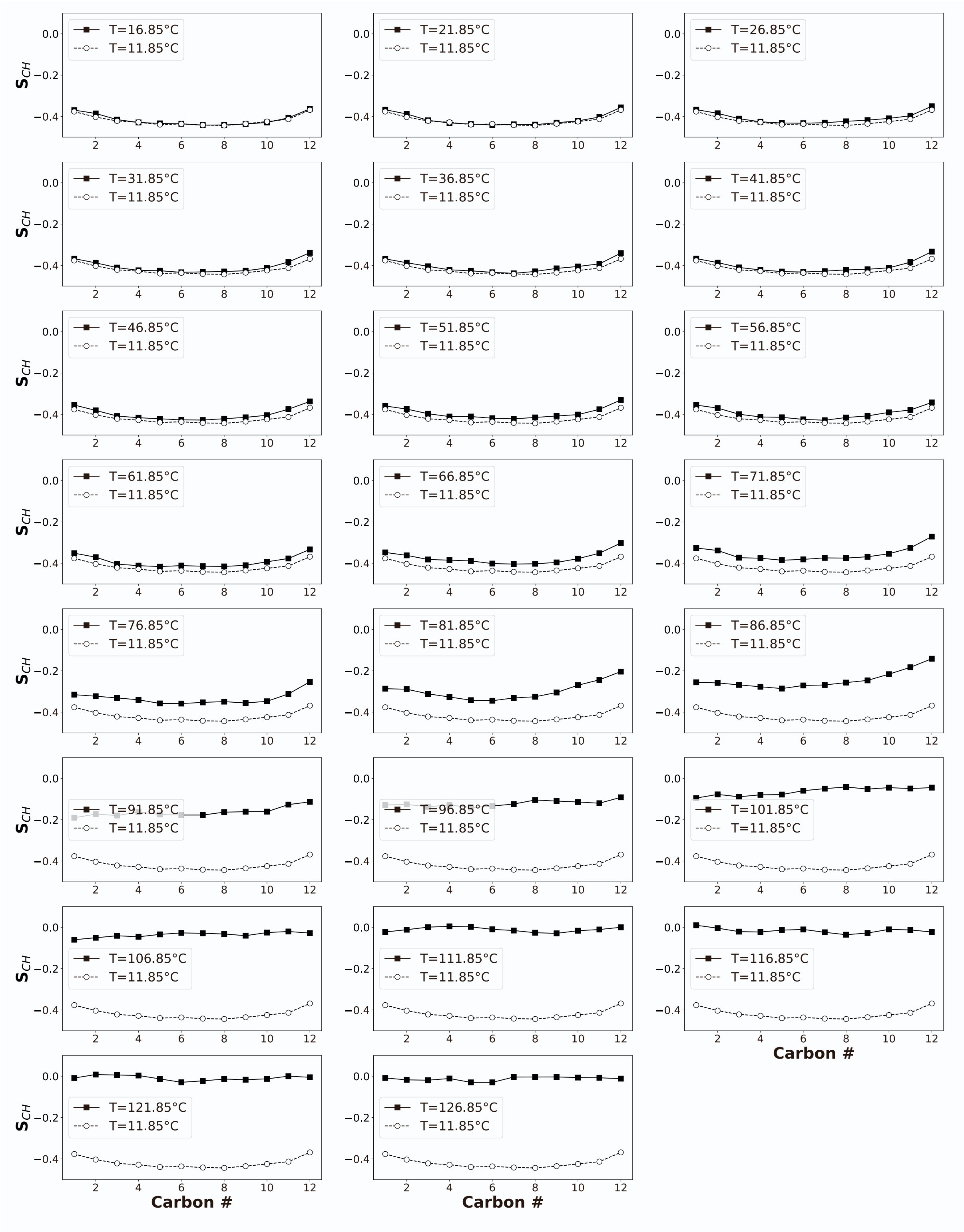

Figure S8. S_{CH} order parameter calculated for the sphingosine chain of CER NS molecules in the four inner leaflets of the 3-bilayer membrane (i.e., all leaflets that are not in direct contact with bulk water) calculated at the designated temperatures as described in the Methods section. Carbon numbers correspond to those shown in Figure S1.

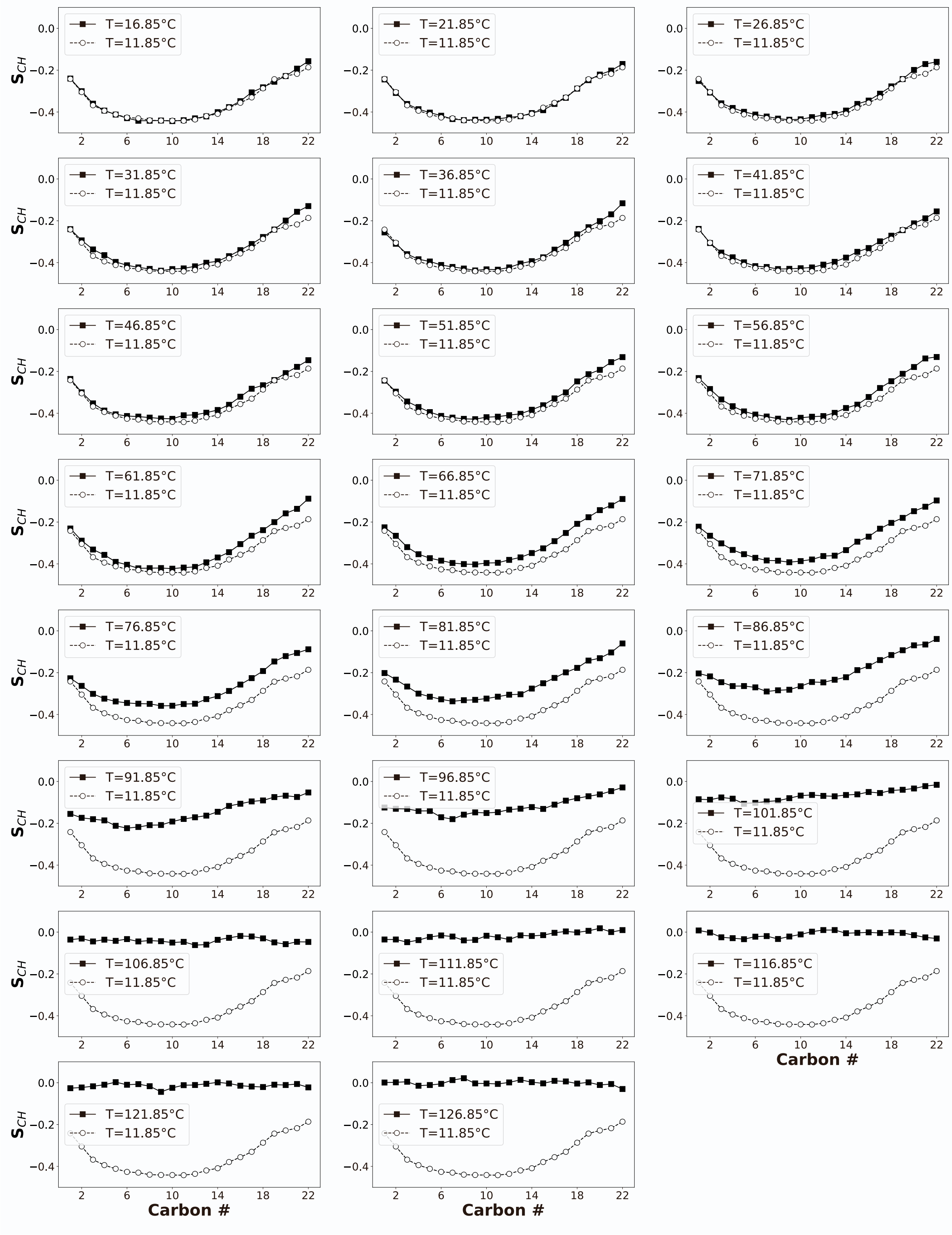

Figure S9. S_{CH} order parameter calculated for the acyl chain of CER NS molecules in the four inner leaflets of the 3-bilayer membrane (i.e., all leaflets that are not in direct contact with bulk water) calculated at the designated temperatures as described in the Methods section. Carbon numbers correspond to those shown in Figure S1.

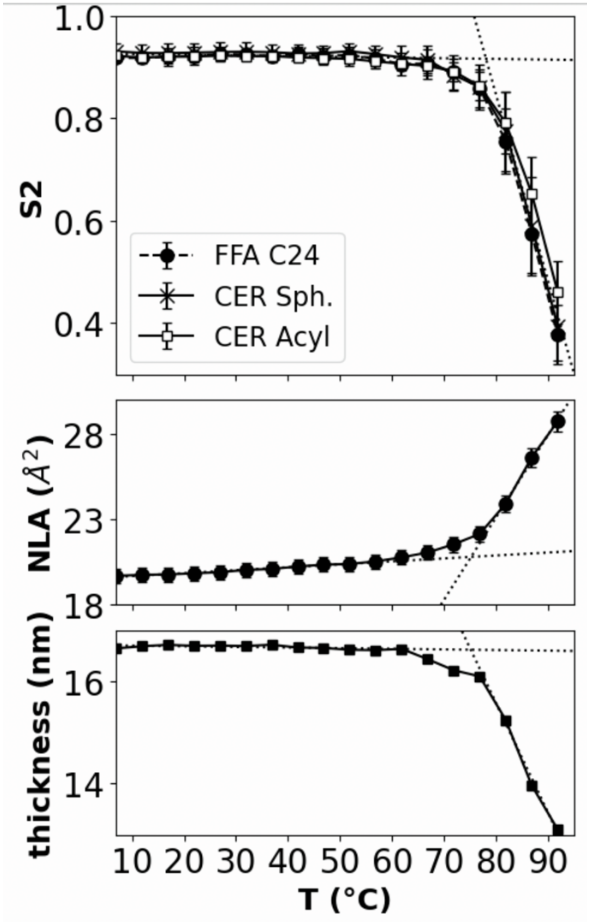

Figure S10. Temperature variation of structural properties calculated from atomistic simulations of the reverse-mapped 3-bilayer membrane containing CER NS C24:CHOL:FFA C24 with a 1:0.5:1 molar ratio heated at a rate of 2.5 °C per ns from 280K. Structural properties obtained using this procedure have very similar transition temperatures and overall behavior as is observed in Figure 8 in the main text, which combines independent heating (32-127ºC) and cooling (32-7ºC) simulations.

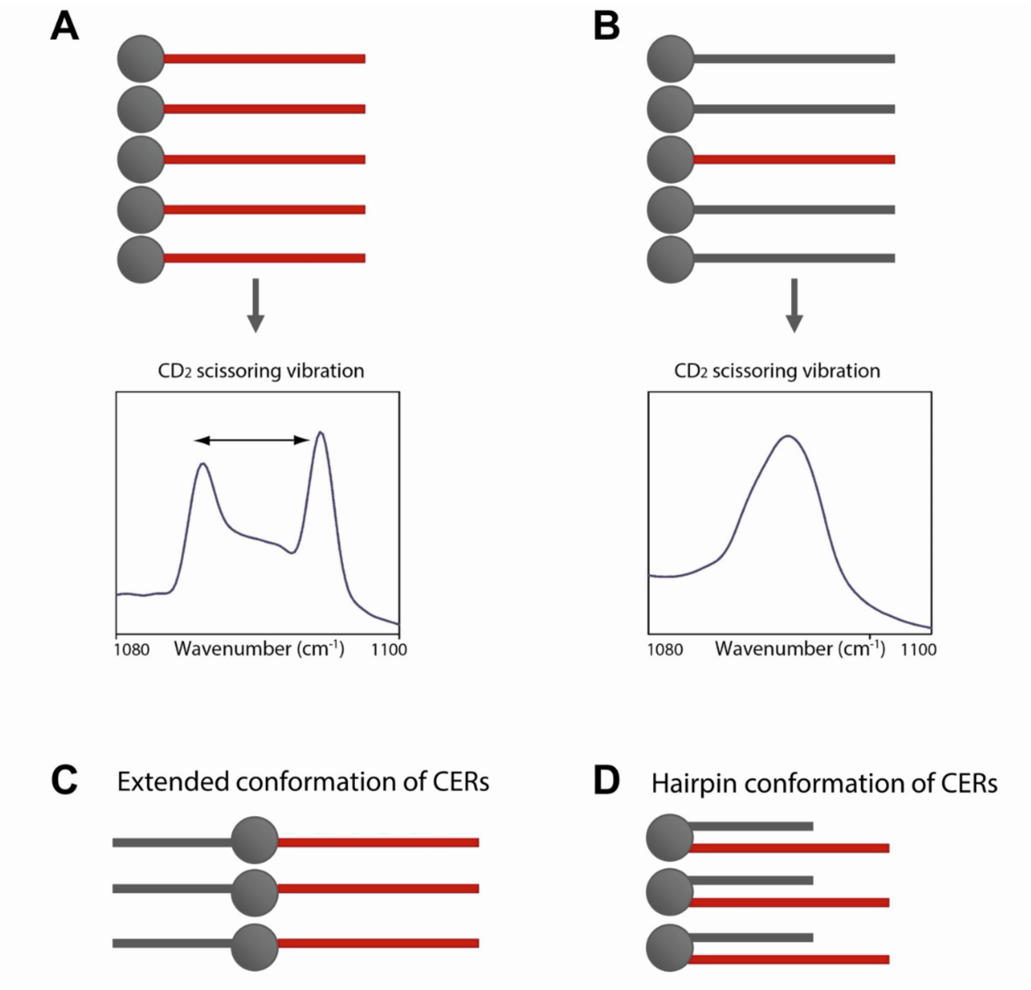

Figure S11. (A) Schematic drawing of the deuterated lipid chains (red) neighboring each other forming large deuterated domains; the large number of CD_2-CD_2 interactions causes two peaks in the FTIR spectrum for the δCD_2 vibrations. (B) When an isolated deuterated lipid chain (red) is present in a protiated lipid chain environment (grey), a single peak occurs in the FTIR spectrum for the δCD_2 vibrations, as there are no CD_2-CD_2 chain interactions. (C) A schematic representation of the extended conformation of CERs, with the acyl chain deuterated (red) and the sphingoid base protiated (grey) located on either side of the CER headgroup. (D) A schematic representation of the CER hairpin conformation, with the two chains on the same side of the headgroup.

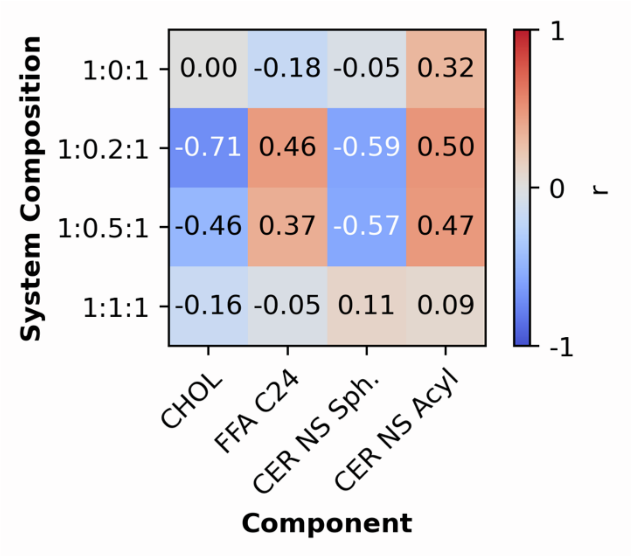

Figure S12. Spearman correlation coefficient between the local fraction of each tail type and local bilayer thickness of the central bilayer in a 3-bilayer system tabulated separately for CHOL, FFA C24, and the sphingosine and acyl tails of CER NS (x axis) for four mixtures at the indicated CER NS:CHOL:FFA molar ratios (y axis). Results are aggregated over four independent simulations at each composition. White text designates |r| > 0.5.

Table S1. Systems studied by composition and method used.

System Composition (CER NS C24: CHOL: FFA C24)	Experiment		Simulation			
	SAXD	FTIR	CG			Reverse-mapped all atom
	Repeat distances and presence of lamellar phases	Phase transition temperatures and tail interactions	Local bilayer thickness and composition	Domain composition using Gaussian mixture clustering	Nearest neighbor analysis	Phase transition temperature
1:0:1	X		X			
1:0.05:1	X					
1:0.1:1	X					
1:0.2:1	X		X	X		
1:0.5:1	X	X	X		X	X
1:1:1	X		X			

Table S2. Comparison of the molar ratios for each lipid tail type (normalized by the average number of CER NS sphingosine tails) and the total number of lipid tails in the central bilayer and two outer bilayers of a 3-bilayer membrane containing a 1:0.5:1 molar ratio mixture of CER NS C24:CHOL:FFA C24. Acyl and sphingosine chains of CER NS are treated separately. Values listed are the mean and standard deviation of the average over the final 200 ns (2000 frames) of 4 replicate simulations.

CER:CHOL:FFA molar ratio	Bilayer(s)	CER NS Acyl	CER NS Sphingosine	CHOL	FFA C24	Total Number of Lipid Tails
1:0:1	Central	0.96 ± 0.07	1.00 ± 0.04		0.85 ± 0.11	942 ± 23
	Outer	1.02 ± 0.07	1.00 ± 0.08		1.05 ± 0.16	2037 ± 26
1:0.2:1	Central	0.99 ± 0.09	1.00 ± 0.10	0.28 ± 0.05	1.23 ± 0.15	941 ± 10
	Outer	1.00 ± 0.18	1.00 ± 0.19	0.20 ± 0.05	0.89 ± 0.23	1954 ± 9
1:0.5:1	Central	0.96 ± 0.04	1.00 ± 0.07	0.49 ± 0.05	0.91 ± 0.05	923 ± 4
	Outer	1.02 ± 0.04	1.00 ± 0.04	0.50 ± 0.03	1.05 ± 0.07	1874 ± 6
1:1:1	Central	1.05 ± 0.06	1.00 ± 0.05	1.14 ± 0.03	1.11 ± 0.05	878 ± 10
	Outer	0.98 ± 0.10	1.00 ± 0.10	0.94 ± 0.05	0.95 ± 0.13	1789 ± 10

Table S3. Raw count of neighboring pairs of lipid tails for the central bilayer of a 3-bilayer membrane containing a 1:0.5:1 molar ratio mixture of CER NS C24:CHOL:FFA C24. Acyl and sphingosine chains of CER NS in the hairpin and extended conformations are treated separately. Values listed are the mean and standard deviation of the average over the final 200 ns (2000 frames) of 4 replicate simulations.

	CER NS Acyl Hairpin	CER NS Acyl Extended	CER NS Acyl All	CER NS Sph Hairpin	CER NS Sph Extended	CER NS Sph All	CHOL	FFA C24
**CER NS Acyl Hairpin**	77.8 ± 4.8	104.3 ± 7.4		328.6 ± 8.1	118.3 ± 6.9		159.8 ± 10.3	223.2 ± 24.7
**CER NS Acyl Extended**	104.3 ± 7.4	35.6 ± 8.4		89.5 ± 8.9	60.7 ± 5.7		85.5 ± 12.8	144.5 ± 16.7
**CER NS Acyl All**			217.7 ± 18.4			597.0 ± 10.7	245.3 ± 13.8	367.7 ± 19.4
**CER NS Sph Hairpin**	328.6 ± 8.1	89.5 ± 8.9		81.8 ± 4.7	109.2 ± 10.3		176.4 ± 10.4	222.4 ± 14.8
**CER NS Sph Extended**	118.3 ± 6.9	60.7 ± 5.7		109.2 ± 10.3	38.0 ± 5.2		107.5 ± 18.4	152.2 ± 17.0
**CER NS Sph All**			597.0 ± 10.7			229.0 ± 14.2	283.9 ± 19.2	374.7 ± 27.3
**CHOL**	159.8 ± 10.3	85.5 ± 12.8	245.3 ± 13.8	176.4 ± 10.4	107.5 ± 18.4	283.9 ± 19.2	66.8 ± 4.0	299.4 ± 8.5
**FFA C24**	223.2 ± 24.7	144.5 ± 16.7	367.7 ± 19.4	222.4 ± 14.8	152.2 ± 17.0	374.7 ± 27.3	299.4 ± 8.5	259.4 ± 21.0

Table S4. Number of each lipid tail type in the central bilayer of a 3-bilayer membrane containing a 1:0.5:1 molar ratio mixture of CER NS C24:CHOL:FFA C24. Acyl and sphingosine tails of CER NS in the hairpin and extended conformations are treated separately. Values listed are the mean and standard deviation of the averages over the final 200 ns (2000 frames) of 4 replicate simulations. These values were used in the calculation of the normalized lipid counts in Table 3 of the paper.

Tail	Count
CER NS Acyl Hairpin	174.50 ± 3.57
CER NS Acyl Extended	89.00 ± 9.51
CER NS Acyl	263.50 ± 7.16
CER NS Sph. Hairpin	174.50 ± 3.57
CER NS Sph. Extended	99.75 ± 6.94
CER NS Sph.	274.25 ± 7.63
CHOL	135.25 ± 5.07
FFA C24	250.00 ± 10.89

Table S5. Raw values of the coordination number representing the number of each lipid tail type (listed by column) that neighbor the reference lipid tail type (listed in the first column) in the central bilayer of a 3-bilayer membrane containing a 1:0.5:1 molar ratio mixture of CER NS C24:CHOL:FFA C24.[a] Acyl and sphingosine chains of CER NS in the hairpin and extended conformations are treated separately. The sum of each row is equal to the total number of neighbors: 7 for CHOL and 6 for all other lipid tails. Values listed are the mean of the average over the final 200 ns (2000 frames) of 4 replicate simulations.

	Raw Mean Number of Neighbors (Coordination Number)						
**Reference Lipid Tail**	**CER NS Acyl Extended**	**CER NS Acyl Hairpin**	**CER NS Sph. Extended**	**CER NS Sph. Hairpin**	**CHOL**	**FFA**	**Sum**
**CER NS Acyl Extended**	0.812	1.073	0.679	0.976	0.861	1.600	6
**CER NS Acyl Hairpin**	0.610	0.950	0.635	1.855	0.709	1.241	6
**CER NS sph. Extended**	0.676	1.059	0.888	1.000	0.947	1.429	6
**CER NS sph. Hairpin**	0.553	1.767	0.567	0.817	0.953	1.343	6
**CHOL**	0.671	1.039	0.789	1.355	0.917	2.228	7
**FFA**	0.607	0.814	0.555	0.92	1.059	2.045	6

[a] Coordination number is calculated by averaging the number of each type of neighboring lipid tail for each reference lipid tail, where a neighbor is defined as one of the 6 nearest neighbors (7 for CHOL), by distance, to the reference tail. This analysis was done using MDTraj (14), Freud (15), and the Numpy (16) Python packages.

Table S6. Normalized coordination numbers representing the number of each of the six lipid tail types (listed by column) that neighbor the reference lipid tail type (listed in the first column) in the central bilayer of a 3-bilayer membrane containing a 1:0.5:1 molar ratio mixture of CER NS C24:CHOL:FFA C24. Acyl and sphingosine chains of CER NS in the hairpin and extended conformations are treated separately. The values listed account for differences in the amounts of each lipid tail type. These were calculated by dividing the raw mean coordination numbers listed in Table S5 by the mole fraction of that lipid tail type (listed in the last row of this table), and then normalizing the resulting numbers so that the sum in each row is equal to the total number of neighbors: 7 for CHOL and 6 for all other lipid tails. For a given row, columns with larger normalized values are more preferred as neighbors to the reference lipid tail listed in the first column. Values listed are the mean of the average over the final 200 ns (2000 frames) of 4 replicate simulations.

	**Normalized Mean Number of Neighbors (Coordination Number)**						
**Reference Lipid Tail**	**CER NS Acyl Extended**	**CER NS Acyl Hairpin**	**CER NS Sph. Extended**	**CER NS Sph. Hairpin**	**CHOL**	**FFA**	**Sum**
**CER NS Acyl Extended**	1.297	0.923	1.084	0.839	0.962	0.894	6
**CER NS Acyl Hairpin**	0.993	0.833	1.033	1.626	0.808	0.707	6
**CER NS Sph. Extended**	1.058	0.892	1.389	0.842	1.037	0.782	6
**CER NS Sph. Hairpin**	0.910	1.564	0.932	0.723	1.097	0.773	6
**CHOL**	1.126	0.939	1.325	1.225	1.077	1.309	7
**FFA**	1.025	0.740	0.937	0.837	1.252	1.209	6
**Composition (mole fraction)**	0.599	1.115	0.599	1.115	0.857	1.714	6

Table S7. Normalized lipid tail pair count ratio from the nearest neighbor analysis (column 3), listed in descending order, compared with the fraction of each lipid tail type that neighbors the reference lipid tail calculated from the normalized coordination numbers. [a,b] Results are for the central bilayer of a 3-bilayer membrane containing a 1:0.5:1 molar ratio mixture of CER NS C24:CHOL:FFA C24. Acyl and sphingosine chains of CER NS in the hairpin (HP) and extended (Ext) conformations are treated separately. Values are for the central bilayer in the 3-bilayer stack presented as the mean of the average over the final 200 ns (2000 frames) of 4 replicate simulations. As expected, the normalized pair count ratio from the nearest neighbor analysis provides similar results to the normalized coordination number fraction.

**Lipid Tail 1:Lipid Tail 2 normalized pair count ratio**			**Normalized coordination number fraction of the 6 lipid tail types (listed as either lipid tail 1 or lipid tail 2) that neighbor the reference lipid tail type [b,c]**					
			**Reference lipid tail type**					
**Lipid Tail 1**	**Lipid Tail 2**	**Normalized pair count ratio [a]**	**CER NS Acyl (HP)**	**CER Acyl NS (Ext)**	**CER NS Sph. (HP)**	**CER NS Sph. (Ext)**	**CHOL**	**FFA**
**CER NS Sph. (HP)**	**FFA**	0.738			0.129			0.140
**CER NS Acyl (HP)**	**FFA**	0.740	0.118					0.123
**CER NS Acyl (HP)**	**CER NS Acyl (HP)**	0.744	0.139					
**CER NS Sph. (HP)**	**CER NS Sph. (HP)**	0.782			0.121			
**CER NS Acyl (Ext)**	**CER NS Sph. (HP)**	0.834		0.140	0.152			
**CER NS Sph. (Ext)**	**FFA**	0.883				0.130		0.156
**CER NS Sph. (HP)**	**CER NS Sph. (Ext)**	0.908			0.155	0.140		
**CER NS Acyl (Ext)**	**FFA**	0.940		0.149				***0.171***
**CER NS Acyl (HP)**	**CER NS Acyl (Ext)**	0.972	0.166	0.154				
**CER NS Acyl (HP)**	**CHOL**	0.980	0.135				0.134	
**CER NS Acyl (HP)**	**CER NS Sph. (Ext)**	0.983	***0.172***			0.149		
**CER NS Acyl (Ext)**	**CER NS Sph. (Ext)**	0.989		***0.181***		***0.176***		
**CER NS Acyl (Ext)**	**CHOL**	***1.028***		0.160			0.161	
**CHOL**	**CHOL**	***1.065***					0.154	

Lipid Tail 1:Lipid Tail 2 normalized pair count ratio			Normalized coordination number fraction of the 6 lipid tail types (listed as either lipid tail 1 or lipid tail 2) that neighbor the reference lipid tail type [b,c]					
			Reference lipid tail type					
Lipid Tail 1	Lipid Tail 2	Normalized pair count ratio [a]	CER NS Acyl (HP)	CER Acyl NS (Ext)	CER NS Sph. (HP)	CER NS Sph. (Ext)	CHOL	FFA
CER NS Sph. (HP)	CHOL	***1.081***			***0.183***		***0.175***	
CER NS Sph. (Ext)	CER NS Sph. (Ext)	***1.116***				***0.232***		
CER NS Sph. (Ext)	CHOL	***1.153***				***0.173***	***0.189***	
FFA	FFA	***1.206***						***0.202***
CHOL	FFA	***1.281***					***0.187***	***0.209***
CER NS Acyl (Ext)	CER NS Acyl (Ext)	***1.315***		***0.216***				
CER NS Acyl (HP)	CER NS Sph. (HP)	***1.561***	***0.271***		***0.261***			

[a] The normalized pair count ratio (column 3) indicates the observed occurrence of a lipid tail pair relative to the probability that the lipid tail pair forms based on the numbers of the two lipid tails that form the pair. The numbers listed are the same as those listed in Table 3 of the paper. Values < 1 indicate the lipid chain pair formed less frequently than expected (the lipid tail pair was preferred less); values > 1 (denoted in bold italics) indicate the lipid tail pair formed more frequently than expected (the lipid chain pair was preferred). For example, the most preferred chain pair is the sphingosine and acyl chains of CER NS in the hairpin conformation (normalized pair count ratio = 1.561). The least preferred chain pairs were FFA with either the sphingosine chain or acyl chain of CER NS in the hairpin conformation (normalized pair ratio = 0.738 and 0.740, respectively).

[b] The normalized coordination number fraction of the six lipid tail types that neighbor the reference lipid tail indicates whether a lipid tail type is a neighbor to the reference tail type less often (values < 1/6 = 0.167) or more often (values > 0.167) than would be expected based on the numbers of the lipid tail types. For example, for the acyl chain of CER NS in the hairpin conformation, the sphingosine chain of CER NS in the hairpin conformation is the most preferred neighbor (normalized coordination number fraction = 0.271) and FFA is the least preferred neighbor (normalized coordination number fraction = 0.118). For the FFA chain, CHOL is the most preferred neighbor followed closely by FFA (normalized coordination number fraction = 0.209 and 0.202, respectively), and the acyl chain of CER NS is the least preferred neighbor (normalized coordination number fraction = 0.123)

[c] The normalized mean coordination number fractions of the six lipid tail types that neighbor the reference lipid tail are calculated from the normalized coordination numbers listed in Table S6 divided by the total number of neighbors (7 for CHOL and 6 for all other lipid tails) for (a) lipid tail 2 when tail 1 is the reference lipid tail type (listed under the column for lipid tail 1), and (b) lipid tail 1 when lipid tail 2 is the reference lipid tail type (listed under the column for lipid tail 2). For example, for CHOL as lipid tail 1 and FFA as lipid tail 2, the normalized coordination number fraction listed for CHOL is the normalized coordination number for FFA when CHOL is the reference lipid tail (1.309) divided by 7 (= 0.187); the normalized coordination number listed for FFA is the normalized coordination number for CHOL when FFA is the reference lipid tail (1.252) divided by 6 (= 0.209). When CHOL is both lipid tail 1 and lipid tail 2, then the number listed for CHOL is 1.077 divided 7 (0.154).

References

1. Shamaprasad, P., T.C. Moore, D. Xia, C.R. Iacovella, A.L. Bunge, and C. McCabe. 2022. Multiscale simulation of ternary stratum corneum lipid mixtures: effects of cholesterol composition. *Langmuir*. 38:7496–7511.

2. Glaser, J., T.D. Nguyen, J.A. Anderson, P. Lui, F. Spiga, J.A. Millan, D.C. Morse, and S.C. Glotzer. 2015. Strong scaling of general-purpose molecular dynamics simulations on GPUs. *Comput. Phys. Commun.* 192:97–107.

3. Moore, T.C., C.R. Iacovella, and C. McCabe. 2014. Derivation of coarse-grained potentials via multistate iterative Boltzmann inversion. *J. Chem. Phys.* 140:224104.

4. Moore, T.C., C.R. Iacovella, and C. McCabe. 2016. Development of a coarse-grained water forcefield via multistate iterative Boltzmann inversion. In: Snurr R, C Adjiman, D Kofke, editors. Foundations of Molecular Modeling and Simulation. Molecular Modeling and Simulation. Singapore: Springer. pp. 37–52.

5. Moore, T.C., C.R. Iacovella, R. Hartkamp, A.L. Bunge, and C. McCabe. 2016. A coarse-grained model of stratum corneum lipids: free fatty acids and ceramide NS. *J. Phys. Chem. B*. 120:9944–9958.

6. Moore, T.C., C.R. Iacovella, A.C. Leonhard, A.L. Bunge, and C. McCabe. 2018. Molecular dynamics simulations of stratum corneum lipid mixtures: A multiscale perspective. *Biochem. Biophys. Res. Commun.* 498:313–318.

7. Hoover, W.G. 1985. Canonical dynamics: Equilibrium phase-space distributions. *Phys. Rev. A*. 31:1695–1697.

8. Parrinello, M., and A. Rahman. 1981. Polymorphic transitions in single crystals: A new molecular dynamics method. *J. Appl. Phys.* 52:7182–7190.

9. Martyna, G.J., D.J. Tobias, and M.L. Klein. 1994. Constant pressure molecular dynamics algorithms. *J. Chem. Phys.* 101:4177–4189.

10. Cao, J., and G.J. Martyna. 1996. Adiabatic path integral molecular dynamics methods. II. Algorithms. *J. Chem. Phys.* 104:2028–2035.

11. Tuckerman, M.E., J. Alejandre, R. López-Rendón, A.L. Jochim, and G.J. Martyna. 2006. A Liouville-operator derived measure-preserving integrator for molecular dynamics simulations in the isothermal–isobaric ensemble. *J. Phys. Math. Gen.* 39:5629–5651.

12. Yu, T.-Q., J. Alejandre, R. López-Rendón, G.J. Martyna, and M.E. Tuckerman. 2010. Measure-preserving integrators for molecular dynamics in the isothermal–isobaric ensemble derived from the Liouville operator. *Chem. Phys.* 370:294–305.

13. Vermeer, L.S., B.L. de Groot, V. Réat, A. Milon, and J. Czaplicki. 2007. Acyl chain order parameter profiles in phospholipid bilayers: computation from molecular dynamics simulations and comparison with 2H NMR experiments. *Eur. Biophys. J.* 36:919–931.

14. McGibbon, R.T., K.A. Beauchamp, M.P. Harrigan, C. Klein, J.M. Swails, C.X. Hernández, C.R. Schwantes, L.-P. Wang, T.J. Lane, and V.S. Pande. 2015. MDTraj: a modern open library for the analysis of molecular dynamics trajectories. *Biophys. J.* 109:1528–1532.

15. Ramasubramani, V., B.D. Dice, E.S. Harper, M.P. Spellings, J.A. Anderson, and S.C. Glotzer. 2020. freud: A software suite for high throughput analysis of particle simulation data. *Comput. Phys. Commun.* 254:107275.

16. Harris, C.R., K.J. Millman, S.J. van der Walt, R. Gommers, P. Virtanen, D. Cournapeau, E. Wieser, J. Taylor, S. Berg, N.J. Smith, R. Kern, M. Picus, S. Hoyer, M.H. van Kerkwijk, M. Brett, A. Haldane, J.F. del Río, M. Wiebe, P. Peterson, P. Gérard-Marchant, K. Sheppard, T. Reddy, W. Weckesser, H. Abbasi, C. Gohlke, and T.E. Oliphant. 2020. Array programming with NumPy. *Nature*. 585:357–362.
